# Exergaming for dementia and mild cognitive impairment

**DOI:** 10.1002/14651858.CD013853.pub2

**Published:** 2024-09-25

**Authors:** Alexandra Voinescu, Themis Papaioannou, Karin Petrini, Danaë Stanton Fraser

**Affiliations:** Department of PsychologyUniversity of BathBathUK; Centre for the Analysis of Motion, Entertainment Research and ApplicationsUniversity of BathBathUK

**Keywords:** Aged, Aged, 80 and over, Humans, Activities of Daily Living, Bias, Cognition, Cognitive Dysfunction, Cognitive Dysfunction/therapy, Dementia, Dementia/psychology, Dementia/therapy, Exercise Therapy, Exercise Therapy/methods, Exergaming, Quality of Life, Randomized Controlled Trials as Topic

## Abstract

**Background:**

Dementia and mild cognitive impairment are significant contributors to disability and dependency in older adults. Current treatments for managing these conditions are limited. Exergaming, a novel technology‐driven intervention combining physical exercise with cognitive tasks, is a potential therapeutic approach.

**Objectives:**

To assess the effects of exergaming interventions on physical and cognitive outcomes, and activities of daily living, in people with dementia and mild cognitive impairment.

**Search methods:**

On 22 December 2023, we searched the Cochrane Dementia and Cognitive Improvement Group's register, MEDLINE (Ovid SP), Embase (Ovid SP), PsycINFO (Ovid SP), CINAHL (EBSCOhost), Web of Science Core Collection (Clarivate), LILACS (BIREME), ClinicalTrials.gov, and the WHO (World Health Organization) meta‐register the International Clinical Trials Registry Portal.

**Selection criteria:**

We included randomised controlled trials (RCTs) that recruited individuals diagnosed with dementia or mild cognitive impairment (MCI). Exergaming interventions involved participants being engaged in physical activity of at least moderate intensity, and used immersive and non‐immersive virtual reality (VR) technology and real‐time interaction. We planned to classify comparators as inactive control group (e.g. no treatment, waiting list), active control group (e.g. standard treatment, non‐specific active control), or alternative treatment (e.g. physical activity, computerised cognitive training). Outcomes were to be measured using validated instruments.

**Data collection and analysis:**

Two review authors independently selected studies for inclusion, extracted data, assessed the risk of bias using the Cochrane risk of bias tool RoB 2, and assessed the certainty of the evidence using GRADE. We consulted a third author if required. Where possible, we pooled outcome data using a fixed‐effect or random‐effects model. We expressed treatment effects as standardised mean differences (SMDs) for continuous outcomes and as risk ratios (RRs) for dichotomous outcomes, along with 95% confidence intervals (CIs). When data could not be pooled, we presented a narrative synthesis.

**Main results:**

We included 11 studies published between 2014 and 2023. Six of these studies were pre‐registered. Seven studies involved 308 participants with mild cognitive impairment, and five studies included 228 individuals with dementia. One of the studies presented data for both MCI and dementia separately. Most comparisons exhibited a high risk or some concerns of bias. We have only low or very low certainty about all the results presented below.

**Effects of exergaming interventions for people with dementia**

**Compared to a control group**

Exergaming may improve global cognitive functioning at the end of treatment, but the evidence is very uncertain (SMD 1.47, 95% 1.04 to 1.90; 2 studies, 113 participants). The evidence is very uncertain about the effects of exergaming at the end of treatment on global physical functioning (SMD −0.20, 95% −0.57 to 0.17; 2 studies, 113 participants) or activities of daily living (ADL) (SMD −0.28, 95% −0.65 to 0.09; 2 studies, 113 participants).

The evidence is very uncertain about adverse effects due to the small sample size and no events. Findings are based on two studies (113 participants), but data could not be pooled; both studies reported no adverse reactions linked to the intervention or control group.

**Compared to an alternative treatment group**

At the end of treatment, the evidence is very uncertain about the effects of exergaming on global physical functioning (SMD 0.14, 95% −0.30 to 0.58; 2 studies, 85 participants) or global cognitive functioning (SMD 0.11, 95% −0.33 to 0.55; 2 studies, 85 participants). For ADL, only one study was available (n = 67), which provided low‐certainty evidence of little to no difference between exergaming and exercise.

The evidence is very uncertain about adverse effects of exergaming compared with alternative treatment (RR 7.50, 95% CI 0.41 to 136.52; 2 studies, 2/85 participants).

**Effects of exergaming interventions for people with mild cognitive impairment (MCI)**

**Compared to a controlgroup**

Exergaming may improve global cognitive functioning at the end of treatment for people with MCI, but the evidence is very uncertain, (SMD 0.79, 95% 0.05 to 1.53; 2 studies, 34 participants). The evidence is very uncertain about the effects of exergaming at the end of treatment on global physical functioning (SMD 0.27, 95% −0.41 to 0.94; 2 studies, 34 participants) and ADL (SMD 0.51, 95% −0.01 to 1.03; 2 studies, 60 participants).

The evidence is very uncertain about the effects of exergaming on adverse effects due to a small sample size and no events (0/14 participants). Findings are based on one study.

**Compared to an alternative treatment group**

The evidence is very uncertain about global physical functioning at the end of treatment. Only one study was included (n = 45). For global cognitive functioning, we included four studies (n = 235 participants), but due to considerable heterogeneity (I² = 96%), we could not pool results. The evidence is very uncertain about the effects of exergaming on global cognitive functioning. No study evaluated ADL outcomes.

The evidence is very uncertain about adverse effects of exergaming due to the small sample size and no events (n = 123 participants). Findings are based on one study.

**Authors' conclusions:**

Overall, the evidence is very uncertain about the effects of exergaming on global physical and cognitive functioning, and ADL. There may be an improvement in global cognitive functioning at the end of treatment for both people with dementia and people with MCI, but the evidence is very uncertain. The potential benefit is observed only when exergaming is compared with a control intervention (e.g. usual care, listening to music, health education), and not when compared with an alternative treatment with a specific effect, such as physical activity (e.g. standing and sitting exercises or cycling).

The evidence is very uncertain about the effects of exergaming on adverse effects. All sessions took place in a controlled and supervised environment. Therefore, we do not know if exergaming can be safely used in a home environment, unsupervised.

## Plain language summary

**What are the benefits and risks of exergaming for people with dementia or mild cognitive impairment?**

**Key messages**

• Playing video games that involve physical activity ('exergaming') may help people with dementia and mild cognitive impairment to improve thinking skills such as learning and remembering items, but we are not totally sure about it.

• There is little evidence at present to suggest exergaming might help improve the ability of people with dementia and mild cognitive impairment to walk, keep their balance, or handle daily tasks like paying bills or going shopping.

**What are dementia and mild cognitive impairment?**

Dementia is a condition where the brain changes in a way that affects a person's memory, thinking, and ability to perform daily tasks. It can make it hard for someone to remember things, plan, focus, or communicate. These changes usually get worse over time and can eventually interfere with everyday life.

Mild cognitive impairment means having some trouble with memory and thinking, but not as much as someone with dementia. However, it could lead to dementia later on. Even though people with mild cognitive impairment might struggle with remembering things or thinking clearly, they can still handle everyday tasks like paying bills, going shopping, and keeping their home clean. Essentially, it is like having some memory and thinking difficulties that are not as severe as dementia, but could possibly get worse over time.

**What is exergaming?**

Currently, there are not many treatments that can slow down diseases like dementia. But we do know that staying active, both physically and mentally, throughout life can lower the risk of getting dementia. Some experts think that encouraging people to stay active in both these ways might help lessen dementia symptoms and the effects of mild cognitive impairment. With new technology, we can now use virtual reality and interactive games, called exergames, to provide physical and mental training. Exergames are seen as a good option because they are relatively easy for older adults to use at home, and they can boost motivation.

**What did we want to find out?**

We wanted to know whether playing exergames is more effective than no active treatment or conventional treatments, for improving physical abilities, thinking skills, and the ability to complete daily tasks, in individuals with dementia and mild cognitive impairment.

We also wanted to check if playing exergames has any side effects.

**What did we do?**

We searched through medical research to find studies that compared people who received an exergaming intervention with people who took part in other treatments. In some cases, people using exergaming were compared with people who did not get any special treatment (e.g. they just relaxed), or got treatments that studies have shown to have a positive effect, like aerobic exercise or brain training. We added the results of the studies together where possible, and we judged the quality of these studies, based on factors such as what methods were used and how many people with mild cognitive impairment or dementia were tested.

**What did we find?**

We found 11 studies. They were published between 2014 and 2023. Seven studies involved 308 people with mild cognitive impairment, and five studies involved 228 participants with dementia. One of the studies involved people with mild cognitive impairment and people with dementia.

We found that playing exergames might help improve thinking skills for people with dementia and mild cognitive impairment when compared to people that received usual care or interventions that were not very active, but not when compared to people receiving active treatments that we know have an effect.

We did not find that playing exergames improved physical abilities, such as walking or balancing, or the ability to do normal daily activities, such as shopping, for people with dementia and mild cognitive impairment.

In general, it appears that engaging in exergames may be safe for individuals with dementia and mild cognitive impairment when carried out in a controlled and supervised environment with help from healthcare professionals. We have no evidence about the safety of exergaming for people with dementia or MCI in a home environment without supervision.

**What are the limitations of the evidence?**

We looked at how well these studies were carried out, and we found that there were some problems with them. This means we need to be careful when interpreting the results mentioned above. One reason for this is that the studies did not include many participants.

**How up to date is this evidence?**

The evidence is up to date to 22 December 2023.

## Summary of findings

**Summary of findings 1:** Summary of findings table: exergaming compared to control for people with dementia at the end of treatment

**Exergaming compared to control for people with dementia at the end of treatment**						
**Patient or population:** people with dementia at the end of treatment **Setting:** clinics (e.g. memory) and long‐term care facilities **Intervention:** exergaming **Comparison:** control						
**Outcomes**	Anticipated absolute effects^*^ (95% CI)		**Relative effect (95% CI)**	**№ of participants (studies)**	**Certainty of the evidence (GRADE)**	**Comments**
	**Risk with control**	**Risk with exergaming**				
Global physical functioning assessed with: 10‐Meter Walk test, Timed Up & Go Test, Five Times Sit‐to‐Stand Test, Gait Speed Follow‐up: range 12 to 24 weeks	‐	SMD **0.2 SD lower** (0.57 lower to 0.17 higher)	‐	113 (2 RCTs)	⊕⊝⊝⊝ Very low^a,b,c,d^	The evidence is very uncertain about the effect of exergaming vs control interventions on global physical functioning. Higher scores reflect better physical functioning.
Global cognitive functioning assessed with: Trail Making Test Part A & B, Stroop, Wechsler Adult Intelligence Scale‐III Digit Span & Spatial Span, Location Learning Test, Letter Fluency, Rule Shift Card Test, Montreal Cognitive Assessment Follow‐up: range 12 to 24 weeks	‐	SMD **1.47 SD higher** (1.04 higher to 1.9 higher)	‐	113 (2 RCTs)	⊕⊝⊝⊝ Very low^b,c,e^	Exergaming may improve global cognitive functioning compared with contol interventions, but the evidence is very uncertain. Higher scores reflect better cognitive functioning.
ADL assessed with: Katz Index Follow‐up: range 12 to 24 weeks	‐	SMD **0.28 SD lower** (0.65 lower to 0.09 higher)	‐	113 (2 RCTs)	⊕⊝⊝⊝ Very low^a,c,d^	The evidence is very uncertain about the effect of exergaming vs control interventions on ADL. Higher scores reflect better ADL.
Adverse effects Follow‐up: median 12 weeks	2 RCTs reported no adverse reactions linked to the intervention and control groups		‐	113 (2 RCTs)	⊕⊝⊝⊝ Very low^c,e,f^	The evidence is very uncertain about the effects of exergaming on adverse effects because the sample size is small and there were no events in either group. Higher scores reflect the occurrence of adverse effects.
*The risk in the intervention group (and its 95% confidence interval) is based on the assumed risk in the comparison group and the **relative effect** of the intervention (and its 95% CI). **ADL:** activities of daily living; **CI:** confidence interval; **RCT:** randomised controlled trial; **SMD:** standardised mean difference; **vs:** versus						
**GRADE Working Group grades of evidence** **High certainty:** we are very confident that the true effect lies close to that of the estimate of the effect. **Moderate certainty:** we are moderately confident in the effect estimate: the true effect is likely to be close to the estimate of the effect, but there is a possibility that it is substantially different. **Low certainty:** our confidence in the effect estimate is limited: the true effect may be substantially different from the estimate of the effect. **Very low certainty:** we have very little confidence in the effect estimate: the true effect is likely to be substantially different from the estimate of effect.						
See interactive version of this table: https://gdt.gradepro.org/presentations/#/isof/isof_question_revman_web_436640451052753607.						

^a^Downgraded one level as 1 study was at high risk of bias and 1 study had some concerns ^b^Downgraded one level for inconsistency as substantial heterogeneity was present ^c^Downgraded one level for imprecision as fewer than 400 participants ^d^Downgraded one level for imprecision as 95% CIs included both a clinically important and a negligible effect ^e^Downgraded one level as 2 studies had some concerns of risk of bias ^f^Downgraded one level for imprecision as there were no events in either group

**Summary of findings 2:** Summary of findings table: exergaming compared to alternative treatment for people with dementia at the end of treatment

**Exergaming compared to alternative treatment for people with dementia at the end of treatment**						
**Patient or population:** people with dementia at the end of treatment **Setting:** memory clinics and long‐term care facilities **Intervention:** exergaming **Comparison:** alternative treatment						
**Outcomes**	Anticipated absolute effects^*^ (95% CI)		**Relative effect (95% CI)**	**№ of participants (studies)**	**Certainty of the evidence (GRADE)**	**Comments**
	**Risk with alternative treatment**	**Risk with exergaming**				
Global physical functioning assessed with: 10‐Meter Walk Test, Timed Up & Go Test, Five Times Sit‐to‐Stand Test, One Minute Sit to Stand Test Follow‐up: range 12 to 36 weeks	‐	SMD **0.14 SD higher** (0.3 lower to 0.58 higher)	‐	85 (2 RCTs)	⊕⊝⊝⊝ Very low^a,b,c^	The evidence is very uncertain about the effect of exergaming vs alternative treatment interventions on global physical functioning. Higher scores reflect better physical functioning.
Global cognitive functioning assessed with: Trail Making Test part A & B, Stroop, Wechsler Adult Intelligence Scale‐III Digit Span & Spatial Span, Location Learning Test, Letter Fluency, Rule Shift Card Test, Mini‐Mental State Exam Follow‐up: range 12 to 24 weeks	‐	SMD **0.11 SD higher** (0.33 lower to 0.55 higher)	‐	85 (2 RCTs)	⊕⊝⊝⊝ Very low^b,c,d,e^	The evidence is very uncertain about the effect of exergaming vs alternative treatment interventions on global cognitive functioning. Higher scores reflect better cognitive functioning.
ADL assessed with: Katz Index Follow‐up: median 12 weeks	1 RCT found little to no difference between groups			67 (1 RCT)	⊕⊕⊝⊝ Low^b,f^	There may be little to no difference in ADL compared with alternative treatment. Higher scores reflect better ADL.
Adverse effects Follow‐up: range 12 to 36 weeks	45 per 1000	**341 per 1000** (19 to 1000)	**RR 7.50** (0.41 to 136.52)	85 (2 RCTs)	⊕⊝⊝⊝ Very low^b,g,h^	The evidence is very uncertain about the effects of exergaming on adverse effects. Higher scores reflect the occurrence of adverse effects.
*The risk in the intervention group (and its 95% confidence interval) is based on the assumed risk in the comparison group and the **relative effect** of the intervention (and its 95% CI). **ADL:** activities of daily living; **CI:** confidence interval; **RR:** risk ratio; **RCT:** randomised controlled trial; **SMD:** standardised mean difference; **vs:** versus						
**GRADE Working Group grades of evidence** **High certainty:** we are very confident that the true effect lies close to that of the estimate of the effect. **Moderate certainty:** we are moderately confident in the effect estimate: the true effect is likely to be close to the estimate of the effect, but there is a possibility that it is substantially different. **Low certainty:** our confidence in the effect estimate is limited: the true effect may be substantially different from the estimate of the effect. **Very low certainty:** we have very little confidence in the effect estimate: the true effect is likely to be substantially different from the estimate of effect.						
See interactive version of this table: https://gdt.gradepro.org/presentations/#/isof/isof_question_revman_web_436640827411060029.						

^a^Downgraded one level as 2 studies were at high risk of bias ^b^Downgraded one level for imprecision as fewer than 400 participants ^c^Downgraded one level for imprecision as 95% CIs included both a clinically important and a negligible effect ^d^Downgraded one level as 1 study was at high risk of bias and 1 study had some concerns ^e^The direction of the effect was inconsistent across studies. ^f^Downgraded one level as 1 study was at high risk of bias ^g^Downgraded one level as 2 studies had some concerns of risk of bias ^h^The confidence interval includes both appreciable harm and appreciable benefit (i.e. 95% CI spans 1); very wide 95% CI indicating uncertain results.

**Summary of findings 3:** Summary of findings table: exergaming compared to control for people with MCI at the end of treatment

**Exergaming compared to control for people with MCI at the end of treatment**						
**Patient or population:** people with MCI at the end of treatment **Setting:** clinics (memory) and community **Intervention:** exergaming **Comparison:** control						
**Outcomes**	Anticipated absolute effects^*^ (95% CI)		**Relative effect (95% CI)**	**№ of participants (studies)**	**Certainty of the evidence (GRADE)**	**Comments**
	**Risk with control**	**Risk with exergaming**				
Global physical functioning assessed with: 6‐Meter Walk Test, Walk Speed, Stride Duration and Length, Stance Phase Duration, Swing Phase Duration, Single Support time, Double Support Time Follow‐up: range 14 to 24 weeks	‐	SMD **0.27 SD higher** (0.41 lower to 0.94 higher)	‐	34 (2 RCTs)	⊕⊝⊝⊝ Very low^a,b,c^	The evidence is very uncertain about the effect of exergaming vs control interventions on global physical functioning. Higher scores reflect better physical functioning.
Global cognitive functioning assessed with: Computer Assessment of Mild Cognitive Impairment, Digit Span Forward & Backward, Trail Making Test part A & B, Test of Attentional Performance, Subtest Logical Memory Wechsler Memory Scale‐IV, HOTAP, Mental Rotation Task Follow‐up: range 14 to 24 weeks	‐	SMD **0.79 SD higher** (0.05 higher to 1.53 higher)	‐	34 (2 RCTs)	⊕⊝⊝⊝ Very low^a,b,d^	Exergaming may improve global cognitive functioning compared with control interventions, but the evidence is very uncertain. Higher scores reflect better cognitive functioning.
ADL assessed with: Timed Instrumental Activities of Daily Living, Seoul‐Instrumental Activities of Daily Living Follow‐up: range 12 to 24 weeks	‐	SMD **0.51 higher** (0.01 lower to 1.03 higher)	‐	60 (2 RCTs)	⊕⊝⊝⊝ Very low^a,b,c^	The evidence is very uncertain about the effect of exergaming vs control interventions on ADL. Higher scores reflect better ADL.
Adverse effects Follow‐up: median 14 weeks	1 RCT reported no adverse reactions linked to the intervention and control group		‐	14 (1 RCT)	⊕⊝⊝⊝ Very low^b,e,f^	The evidence is very uncertain about the effects of exergaming on adverse effects because the sample size is small and there were no events in either group. Higher scores reflect the occurrence of adverse effects.
*The risk in the intervention group (and its 95% confidence interval) is based on the assumed risk in the comparison group and the **relative effect** of the intervention (and its 95% CI). **ADL:** activities of daily living; **CI:** confidence interval; **MCI:** mild cognitive impairment; **RCT:** randomised controlled trial; **SMD:** standardised mean difference; **vs:** versus						
**GRADE Working Group grades of evidence** **High certainty:** we are very confident that the true effect lies close to that of the estimate of the effect. **Moderate certainty:** we are moderately confident in the effect estimate: the true effect is likely to be close to the estimate of the effect, but there is a possibility that it is substantially different. **Low certainty:** our confidence in the effect estimate is limited: the true effect may be substantially different from the estimate of the effect. **Very low certainty:** we have very little confidence in the effect estimate: the true effect is likely to be substantially different from the estimate of effect.						
See interactive version of this table: https://gdt.gradepro.org/presentations/#/isof/isof_question_revman_web_436645333180682382.						

^a^Downgraded one level as 2 studies were at high risk of bias ^b^Downgraded one level for imprecision as fewer than 400 participants ^c^Downgraded one level for imprecision as 95% CIs included both a clinically important and a negligible effect ^d^Downgraded one level for inconsistency as moderate heterogeneity was present ^e^Downgraded one level as 1 study had some concerns of risk of bias ^f^Downgraded one level for imprecision as there were no events in either group

**Summary of findings 4:** Summary of findings table: exergaming compared to alternative treatment for people with MCI at the end of treatment

**Exergaming compared to alternative treatment for people with MCI at the end of treatment**						
**Patient or population:** people with MCI at the end of treatment **Setting:** rehabilitation centres, retirement homes, clinics and community **Intervention:** exergaming **Comparison:** alternative treatment						
**Outcomes**	Anticipated absolute effects^*^ (95% CI)		**Relative effect (95% CI)**	**№ of participants (studies)**	**Certainty of the evidence (GRADE)**	**Comments**
	**Risk with alternative treatment**	**Risk with exergaming**				
Global physical functioning assessed with: 6‐Minute Walk Test, Timed Up & Go Test, 10‐Meter Walk Test, Functional Reach Test Follow‐up: median 12 weeks	1 RCT found little or no difference between groups		‐	45 (1 RCT)	⊕⊕⊝⊝ Low^a,b^	The evidence suggests that exergaming vs alternative treatment results in little to no difference in global physical functioning. Higher scores reflect better physical functioning.
Global cognitive outcomes assessed with: Mini‐Mental State Exam, Montreal Cognitive Assessment, Trail Making Test part A & B, Verbal Fluency Test, Digit Span Forward & Backward, Rey Auditory Verbal Learning Test, Rey–Osterrieth Complex FigureTest, Wechsler Adult Intelligence Scale‐Block Design Test, Stroop, N‐Back, Loewenstein Occupational Therapy Cognitive Assessment‐Geriatric Follow‐up: range 6 to 12 weeks	2 RCTs (n = 92) reported beneficial effects of exergaming vs alternative treatment, 1 RCT (n = 78) reported mixed or unclear findings, and 1 RCT (n = 45) found little or no difference between groups		‐	235 (4 RCTs)	⊕⊝⊝⊝ Very low^b,c,d^	The evidence is very uncertain about the effect of exergaming on global cognitive functioning when compared with alternative treatment. Small studies reported either benefits or no difference between groups. Larger studies reported mixed findings or benefits of exergaming. Higher scores reflect better cognitive functioning.
ADL	‐	‐	‐	‐	‐	Not reported
Adverse effects follow‐up: range 10 to 12 weeks	2 RCTs reported no adverse reactions linked to the intervention and control groups		‐	123 (2 RCTs)	⊕⊝⊝⊝ Very low^b,e,f^	The evidence is very uncertain about the effects of exergaming on adverse effects because the sample size is small and there were no events in either group. Higher scores reflect the occurrence of adverse effects.
*The risk in the intervention group (and its 95% confidence interval) is based on the assumed risk in the comparison group and the **relative effect** of the intervention (and its 95% CI). **CI:** confidence interval; **MCI:** mild cognitive impairment; **RCT:** randomised controlled trial; **SMD:** standardised mean difference; **vs:** versus						
**GRADE Working Group grades of evidence** **High certainty:** we are very confident that the true effect lies close to that of the estimate of the effect. **Moderate certainty:** we are moderately confident in the effect estimate: the true effect is likely to be close to the estimate of the effect, but there is a possibility that it is substantially different. **Low certainty:** our confidence in the effect estimate is limited: the true effect may be substantially different from the estimate of the effect. **Very low certainty:** we have very little confidence in the effect estimate: the true effect is likely to be substantially different from the estimate of effect.						
See interactive version of this table: https://gdt.gradepro.org/presentations/#/isof/isof_question_revman_web_436645842468542893.						

^a^Downgraded one level as 1 study was at high risk of bias ^b^Downgraded one level for imprecision as fewer than 400 participants ^c^Downgraded one level as 4 studies were at high risk of bias or some concerns ^d^Downgraded one level for inconsistency due to wide variation in the effects ^e^Downgraded one level as 2 studies had some concerns of risk of bias ^f^Downgraded one level for imprecision as there were no events in either group

## Background

### Description of the condition

Dementia is a major cause of disability and dependency in the older population, and has an extremely negative impact (e.g. emotional and financial burden) on family, caregivers, community, and residential care services (Shah 2016; Wortmann 2012). In 2015, dementia was estimated to affect about 5% of the older population (defined as those aged 60 years and older), around 46.8 million people worldwide, with this figure forecast to rise to approximately 74.7 million by 2030 (World Alzheimer Report 2015).

Dementia is an umbrella term for a syndrome caused by several diseases, usually of a chronic or progressive nature. It is characterised by significant cognitive decline in one or more cognitive domains (e.g. attention, executive functioning, learning and memory, language, perceptual‐motor function, and social cognition). This decline negatively impacts and significantly affects independence (e.g. personal activities of daily living, such as housework, managing money, shopping, washing, dressing, personal hygiene, feeding (APA 2013)). Alzheimer’s disease is the most common form of dementia, accounting for approximately 70% of dementia cases (APA 2013). Vascular dementia, dementia with Lewy bodies, dementia in Parkinson’s disease, and frontotemporal dementia are other forms of dementia (APA 2013). Mixed pathologies are common.

Mild cognitive impairment (MCI) is also a syndrome of cognitive decline, but it is less severe than dementia. The main criteria for MCI are a subjective concern about decline in cognitive functioning, and an objective alteration of cognitive functions (e.g. memory, executive functioning, attention, language, visuospatial abilities), measured using neuropsychological tests. The key criterion that distinguishes MCI from dementia is the preservation of independence in daily activities, albeit with a decline in efficiency (Langa 2014). MCI has a prevalence of 10% to 20% of the older adult population (defined as 65 years and older) (Langa 2014). A meta‐analysis identified that 32% of people with MCI developed dementia after an average of 4.57 years of follow‐up (Mitchell 2009). Conversion rates from MCI to dementia depend largely on the type of population being studied. For example, participants with MCI from clinical settings have different conversion rates than people with MCI from community settings. After an average of 4.57 years, the community samples converted to dementia at a rate of 21.9%, while 39.2% of those in the clinical samples progressed to dementia (Mitchell 2009).

Besides impairments in cognitive functioning, people with dementia and MCI often experience balance and gait problems, are at high risk of falls, and are more sedentary than healthy people (Allan 2005; Eriksson 2008; Hartman 2018; Taylor 2013).

There are currently limited options for either treating MCI or dementia, or for delaying progression from MCI to dementia. For dementia, approved drugs (e.g. acetylcholinesterase inhibitors, memantine) show small effects on cognition and activities of daily living in the short term (Owens 2020; Winblad 2016). In the case of MCI, there is no evidence that drug therapy (e.g. donepezil or memantine) improves general cognitive functioning (Owens 2020). Non‐pharmacological interventions, such as regular physical exercise and cognitive training, have been identified as potentially effective interventions for reducing the likelihood of developing dementia or MCI, reducing symptoms, or slowing down cognitive decline (Bahar‐Fuchs 2019; Forbes 2015; Gallaway 2017; Heyn 2004; Kane 2017; Lam 2018; Sallis 2016; Selkoe 2012; Song 2018; Wang 2019). However, the extent of their effectiveness remains unclear, as there is a lack of consensus regarding the magnitude of the effects, and the quality of the evidence.

### Description of the intervention

There is growing evidence that combining physical and cognitive exercise training significantly improves global cognitive function. A meta‐analysis, which included data from 10 randomised controlled trials (RCTs) with 742 participants, reported small to medium improvements in global cognitive function with combined cognitive and physical interventions, and medium to large improvements in activities of daily living (ADL) for people with MCI and dementia, compared to control groups (Karssemeijer 2017). However, people with dementia and MCI are more sedentary and have more balance and gait problems, which can result in more falls compared with cognitively healthy older adults (Allan 2005; Eriksson 2008; Hartman 2018; Taylor 2013), it might therefore be more difficult for them to engage in physical activities outside the house. Deterioration in emotional control, social behaviour, or motivation (APA 2013; WHO 2019), which can also appear in dementia, might result in lack of initiative and interest in physical and cognitive activities (Clarke 2008; Crombie 2004). Other barriers that may reduce older adults’ participation in physical activity are the availability and cost of programmes, the reliability and affordability of public transportation, the weather, and concerns about neighbourhood safety (Belza 2004).

A new intervention, which focuses on physical exercise, can incorporate cognitive elements, and can be carried out in the comfort and security of a person’s own home, is virtual reality (VR) exercise training, also known as exergaming (Van Santen 2018). Using various technology devices and software (e.g. personal computers, gaming consoles, TV screens or projectors, head‐mounted displays (HMD), controllers, motion tracking sensors), a game‐like environment (e.g. virtual football court or ski slope), or a more familiar environment (e.g. a virtual city familiar to the participant) is generated. The person is immersed in this virtual environment via motion‐tracking sensors or infrared cameras that capture and project their image and movements in real time on the HMD or on a screen. Using controllers and motion‐tracking technology that records their movement, the person interacts with, and controls the computer‐generated environment, by performing various tasks, usually following rules, keeping score, etc. For example, they may virtually ski down the slope; in order to do this, they have to reproduce body movements that are typical for a skiing session.

Exergaming can facilitate the combination of physical exercise with cognitive tasks. An example of an exergame that combines both physical and cognitive elements is cycle training. Participants cycle in a virtual environment (VE) while performing activities known to target specific cognitive functions (e.g. working memory), such as learning a list of neighbourhood errand locations (e.g. doctor, pharmacy, grocery), and having to follow the correct route to reach that location (Anderson‐Hanley 2017; Anderson‐Hanley 2018).

It has been proposed that exergames can improve and stimulate physical activity amongst people with dementia with better adherence rates than traditional physical activities, with no adverse effects (Karssemeijer 2019a; Karssemeijer 2019b; Van Santen 2018; Wiloth 2018). Exergames were found to produce similar energy expenditure, heart rate, and oxygen consumption levels to traditional physical activities, which makes them potentially effective as a form of light to moderate intensity physical activity (Peng 2011). Technology‐driven tasks, using exergames, require substantial cognitive resources to accomplish tasks, such as navigation, and they offer both cognitive and physical stimulation (Green 2006; Green 2008; Neguţ 2016). High adherence rates, relatively low cost compared to other training programmes, such as gyms, and a safe training environment (e.g. at home) can increase accessibility, especially amongst vulnerable populations, such as individuals with dementia and MCI.

Clinical trials have reported conflicting results on the effects of exergaming on both physical (e.g. Karssemeijer 2019b; Hughes 2014; Padala 2012; Padala 2017), and cognitive outcomes in people with dementia and MCI (e.g. Amjad 2019; Hughes 2014; Karssemeijer 2019a; Padala 2012; Padala 2017; Park 2018).

There are challenges related to the successful implementation of novel technologies to support people with dementia or MCI. Common contributors to hesitancy may be safety and risks from use such as falls and accidents or fatigue. Co‐designing with people with dementia and healthcare practitioners, assessing user experience and usability, measuring cost‐effectiveness, preparing strategies for the implementation of assistive technologies in different care settings and working alongside healthcare professionals may help reduce barriers to implementation (Meiland 2017; Sauchelli 2023).

### How the intervention might work

Meta‐analytical studies have shown that physical exercise has a positive effect on cognitive functions and ADL in people with dementia and MCI, but the evidence that supports this is low quality when assessed by approaches such as GRADE (Forbes 2015; Song 2018; Wang 2019). Multiple pathways have been proposed to explain the facilitating effect of physical activity on cognition: mainly, a reduction of risk factors associated with cardiovascular disease, insulin resistance, obesity, hypertension, and inflammation (Bamidis 2014; Sofi 2011). A second protective mechanism is the neurotrophic effect of physical exercise, which leads to increased neural growth due to a release of neurotrophins, and growth of synapses and dendritic receptors (Bamidis 2014; Gómez‐Pinilla 1998; Sofi 2011).

Various interventions designated to enhance cognition have been developed (e.g. cognitive stimulation, cognitive rehabilitation, and cognitive training). Several meta‐analyses found that cognitive stimulation and training improved general cognition in people with dementia, but the quality of the evidence was low (Aguirre 2013; Bahar‐Fuchs 2019; Woods 2012). A recent Cochrane review reported moderate‐quality evidence showing improvements in cognition for people with dementia after cognitive stimulation (Woods 2023). Some randomised controlled trials have found that cognitive rehabilitation for people with dementia improved global performance, everyday functioning, and satisfaction (Clare 2010; Clare 2019; Regan 2017). A recent Cochrane review showed that cognitive rehabilitation is helpful in enabling people with mild or moderate dementia to enhance their ability to manage daily activities (Kudlicka 2023). The underlying mechanisms proposed to account for positive effects of training or stimulation on cognition are mostly related to brain plasticity, i.e. to the brain’s capacity to modify its structure and function, even at an older age, through several mechanisms: neurogenesis, synaptogenesis, and angiogenesis (Hill 2011).

It has been hypothesised that combining physical and cognitive training could facilitate greater changes in brain plasticity (Bamidis 2014; Fissler 2013). According to Fissler 2013, combining the two forms of training might lead to synergistic effects. A 2017 meta‐analysis showed that combining physical and cognitive training improved cognitive functioning, ADL, and mood among people with MCI and dementia, with low to moderate effect sizes, when compared to control groups (Karssemeijer 2017).

There are several characteristics of exergames that may make them suitable for training and rehabilitating both physical and cognitive abilities.

First, exergames can offer realistic experiences, thus aligning with rehabilitation principles (Levin 2015). For example, learning improves if the tasks are meaningful, specific, and repetitive, and if the task difficulty is increased over time (Kleim 2008; Levin 2015). The technical and software capabilities of exergames allow the number of stimuli and the difficulty of tasks to be adjusted according to the needs and abilities of the player, while maintaining stimulus control and consistency (Mrakic‐Sposta 2018; Mura 2018). Feedback is a key component for motor learning, and facilitates quick self‐correction (Bortone 2018; Da Fonseca 2017; Ferreira 2018), and exergame platform systems can provide real‐time, strategic, and goal‐directed feedback (Mrakic‐Sposta 2018; Rizzo 2019).

Second, exergames can provide environmental enrichment by offering a more engaging and stimulating experience compared to traditional methods. Previous research in animal and human studies pointed out the positive effect of enriched environments on motor and cognitive performance (Clemenson 2015a; Freund 2013; Gelfo 2011; Harvey 2009; Kondo 2008; Maguire 1998; Morgan 2013; Nozari 2014; Zhao 2014). Other studies have also linked performance of various tasks (e.g. navigation) in enriched environments with morphological changes in the brain (e.g. greater cerebral weight and length; (Bayne 2018; Clemenson 2015b; Harvey 2009; Maguire 1998)), and enhanced brain plasticity (Fares 2013).

Third, because exergaming incorporates gaming elements, it has the potential to increase motivation of patients to participate in treatment (Howard 2017; Rizzo 2005). Exergaming may offer more gratifying experiences than traditional rehabilitation interventions (Howard 2017) and may increase willingness to accept the treatment plan (Rose 2018).

### Why it is important to do this review

Evidence is accumulating for the effectiveness of exergaming for improving physical or cognitive outcomes (or both) in older adults (Larsen 2013; Tahmosybayat 2017), people with Parkinson’s disease (Garcia‐Agundez 2019; Harris 2015), and people with neurological disabilities (Mura 2018; Rosly 2017). However, the effect of exergaming on physical, cognitive outcomes, and ADL in people with dementia and MCI remains unclear. The results of RCTs are contradictory. Also, due to the heterogeneity of measures used for physical and cognitive domains, synthesising the results using a meta‐analytical approach will enable us to obtain a clearer picture of the effectiveness of exergaming on global physical, cognitive, and ADL outcomes.

## Objectives

To assess the effects of exergaming interventions on physical and cognitive outcomes, and activities of daily living, in people with dementia and mild cognitive impairment.

## Methods

### Criteria for considering studies for this review

#### Types of studies

We included randomised controlled trials (RCTs) that compared an exergame intervention with a control condition or an alternative treatment. Control conditions could be waiting list, treatment as usual, or a non‐specific intervention that controlled for experimenter contact (e.g. same amount of interaction with the experimenter in both groups), but was not expected to have an effect on key outcomes. Alternative treatments could be traditional physical, cognitive, or combined interventions, etc. Individual‐ or cluster‐randomised trials were eligible for inclusion in the review.

If the exergame intervention was used in addition to usual treatment (e.g. medication), the control and comparison groups had to receive the same usual treatment.

We planned to extract only first‐period data from trials with a cross‐over design; if this was not possible, we planned to exclude the study (Elbourne 2002).

#### Types of participants

Eligible participants were adults (18 years or older) diagnosed as having dementia or mild cognitive impairment (MCI).

Participants with dementia should have been diagnosed using internationally recognised criteria such as DSM‐IV or DSM‐5 (APA 1995; APA 2013), or ICD‐10 (WHO 1992).

There were no exclusions on the basis of subtype (e.g. Alzheimer's disease, vascular dementia, Lewy body dementia) or severity of dementia. To classify the severity of dementia, we planned to use internationally recognised classifications. For example, mild dementia might be a score of 1 on the Clinical Dementia Rating (CDR) scale (Morris 1993), between 21 and 26 on the Mini Mental State Examination (MMSE) (Folstein 1975), or between 18 and 25 on the Montreal Cognitive Assessment (MoCA) (Nasreddine 2005); and moderate dementia, a score of 2 on the CDR, between 10 and 20 on the MMSE, or between 10 and 17 on the MoCA. We did not expect to identify studies that included people with severe dementia due to difficulties in implementing the intervention with this group.

For MCI, we accepted a diagnosis provided by the study authors, based on criteria proposed by Petersen 1999, the National Institute on Aging‐Alzheimer’s Association (Albert 2011), International Working Group on Mild Cognitive Impairment (Winblad 2004), MCI Working Group of the European Consortium on Alzheimer’s Disease (Portet 2006), or similar criteria. The study authors should have described an attempt to exclude dementia.

If studies included a heterogeneous group of participants (e.g. including other neurological diseases, such as stroke or traumatic brain injury, or both dementia and MCI), we included these studies only if data for participants with dementia and MCI were reported separately, or if we could obtain these data from authors.

#### Types of interventions

##### Interventions

We were interested in exergame interventions designed to improve physical and cognitive outcomes and daily functioning (activities of daily living (ADL)) in people with MCI and dementia. Eligible exergaming interventions were those in which the participant engaged in physical activity of at least moderate intensity (Norton 2010), with or without an additional cognitive element, using an interactive, immersive or non‐immersive virtual reality (VR) platform. We included exergaming interventions of any frequency and duration, delivered in any setting (e.g. delivered in a clinic or at the participant’s home).

The World Health Organization (WHO) defines physical activity as all movement produced by skeletal muscles that requires energy expenditure (WHO 2020). Exergaming interventions included physical activity that substantially simulates a recognised sport or dance form, or that includes a structured set of physical activities, designed or chosen to improve some or all of: aerobic fitness, muscle strength, balance, and coordination.

The exergaming interventions inevitably involved some cognitive activity, although this could be non‐specific, e.g. interacting within the game, following rules, keeping score. For example, the Wii skiing game asks players to choose their route when they descend the slope, follow the route, and avoid obstacles and falling down. Based on their performance, they collect points. The player's performance is monitored, and they get instant feedback. We regard this as a form of cognitive stimulation. In addition, exergames may include more specific tasks, which would amount to cognitive rehabilitation, training, or both. For example, participants must learn a list of neighbourhood errand locations (e.g., doctor, pharmacy, grocery) and have to follow the correct route on a virtual bike to reach the locations (Anderson‐Hanley 2017; Anderson‐Hanley 2018). This targets working memory and the real‐life task of completing a shopping trip.

We included any interventions that used an immersive or non‐immersive VR‐based platform, using the definitions provided by Rizzo 2017. Immersive VR includes platforms, such as head‐mounted displays (HMD) and connected automated virtual environments (CAVE), which occlude the view of the outside world. Non‐immersive VR uses platforms that deliver content via standard TV screens, desktop monitors, projectors, etc. The key feature of non‐immersive systems is that the outside world is not occluded from the user’s view. Given this definition, interactive gaming platforms, such as Nintendo Wii, Microsoft Xbox, and Sony PlayStation are non‐immersive VR.

Interaction could be achieved via any type of technology that allows real‐time interaction, by capturing and projecting the participant's image, movements, or both, in real time on a screen. The interaction had to be “beyond what is typically afforded with standard mouse and keyboard interface devices” (Rizzo 2017). This could include controllers, motion tracking sensors, or infrared cameras. We only considered virtual environments (VE) in which the interaction was in real time (e.g. while walking on a treadmill, the speed is adjusted in line with the person’s own pace, and by considering the direction in which the participant is heading). We excluded VEs in which the user did not interact in real time with the VE (e.g. cycling in front of a TV set, with no changes in VE dictated by the user’s behaviour).

##### Comparators

- Inactive control: no intervention, such as a waiting list (participants in the control group receive the exergame intervention at the end of the study), or treatment as usual (participants received usual care only)
- Active control: intervention involves equivalent contact with the researchers, typically for an equivalent number of sessions or visits, but the intervention is not hypothesised to have any specific effect on the study outcomes, such as relaxation, or sessions spent watching documentaries or movies or listening to music
- Alternative treatment control: another treatment hypothesised to have a specific effect on the study outcomes, such as physical activity, cognitive stimulation therapy, cognitive training, arts therapies, multisensory stimulation, multimodal training, reminiscence therapy

#### Types of outcome measures

Our primary outcomes were global measures of physical and cognitive function, and ADL, measured by any validated instrument at the end of the treatment and at later follow‐up. If a global measure was not reported, then we created a composite of relevant outcome measures for each study. We considered a validated instrument to be any measure that was published or used in other studies and we excluded measures created by the research team within studies. Biomarker and economic outcomes were beyond the scope of this review.

##### Primary outcomes

- Global physical functioning: we combined all physical measures from each study
- Global cognitive functioning: we combined all cognitive measures from each study
- Global ADL performance: we combined all ADL outcomes from each study

##### Secondary outcomes

We treated the following as secondary outcomes and categorised them according to well established classifications (e.g. Laver 2017; Lezak 2012).

- Physical functioning outcomes, including the following.
  - Lower limb function, using measures, such as walk tests ‐ 2‐minute walk test (Butland 1982), Timed Up and Go Test (Podsiadlo 1991), Sit to Stand Test (Csuka 1985)
  - Upper limb function, using measures, such as the Fugl Meyer Assessment (Fugl‐Meyer 1975), Box and Block Test (Mathiowetz 1985)
  - Balance and postural control, including assessments, such as the Berg Balance Scale (Berg 1992)
  - Motor function, measured with the Motor Assessment Scale (Carr 1985)
- Cognitive functioning outcomes, including the following.
  - General cognition, using measures such as the Mini Mental State Examination (Folstein 1975), Montreal Cognitive Assessment Test (Nasreddine 2005)
  - Attention, processing speed, and working memory, using the Wechsler Adult Intelligence Scale (WAIS)‐digit span (Wechsler 2008), Corsi Block‐tapping Test (Corsi 1973), WAIS‐ digits backward (Wechsler 2008), N‐Back tasks (e.g. Kirchner 1958), Continuous Performance Test (e.g. Rosvold 1956)
  - Perception (e.g. visual inattention, visual scanning, visual recognition and organisation; auditory perception, inattention) measured using the Behavioral Inattention Test (Wilson 1988), Line Bisection Tests (e.g. Schenkenberg 1980), Cancellation tasks (e.g. Mesulam 2000), Judgement of Line Orientation (Benton 1978), Face Recognition (Benton 1983)
  - Memory (e.g. verbal, visual, incidental, and prospective memory), using measures such as the California Verbal Learning Test (Delis 1987), Rey‐Osterrieth Complex Figure Test (Rey 1941), Wechsler Memory Scale (Wechsler 1987), Rivermead Behavioural Memory Test (Wilson 1985)
  - Verbal functions and language skills (e.g. aphasia, auditory comprehension, naming, vocabulary and verbal comprehension), measured with the Boston Diagnostic Aphasia Examination (Goodglass 1983), Putney Auditory Comprehension Screening Test (Beaumont 1999), Boston Naming Test (Kaplan 1983), WAIS‐vocabulary (Wechsler 2008), Token Test (De Renzi 1978)
  - Reasoning, measured with the WAIS‐Similarities (Wechsler 2008), Category test (e.g. Halstead 1943), D‐KEFS Twenty Questions (Delis 2001)
  - Executive function (e.g. volition, planning and decision‐making, purposive action, verbal, letter, category fluency, perseveration), measured using tests such as the Patient Competency Rating Scale (Prigatano 1986), Self‐Ordered Pointing Test (Petrides 1982), Tower tests (e.g. Shallice 1982), Multiple Errands Test (Shallice 1991), Iowa Gambling Task (Bechara 1994), Wisconsin Card Sorting Test (Grant 1948), Trail Making Tests part B (Reitan 1955), Stroop Tests (e.g. Stroop 1935)
- ADL outcomes, including the following.
  - ADL measures (e.g. Barthel Index scale (Mahoney 1965), Functional Independence Measure (Granger 1989))
  - Instrumental activities of daily living (IADL) (e.g. Instrumental Activities of Daily Living Scale (Graf 2008))
- Quality of life (e.g. Quality of Life in Alzheimer’s Disease (Logsdon 1999))
- Physical activity (e.g. Physical Activity Scale for the Elderly (Washburn 1993))
- Frailty (e.g. Evaluative Frailty Index for Physical Activity (De Vries 2013))
- Adverse effects (e.g. motion sickness, disorientation, headaches, pain, fatigue, injury)
- Falls that occur during the use of the intervention
- Falls that occur outwith the use of the intervention
- Measures of enjoyment and satisfaction with the programme
- Feasibility and treatment adherence (number of dropouts)
- Caregiver outcomes (e.g. burden)

### Search methods for identification of studies

#### Electronic searches

We searched the Cochrane Dementia and Cognitive Improvement Group’s Specialised Register, initially on 28 January 2021, and again on 2 March 2022 and 22 December 2023. The Register is maintained by the Information Specialists of the Cochrane Dementia and Cognitive Improvement Group and contains studies in the areas of dementia (prevention and treatment), mild cognitive impairment and cognitive improvement. The studies are identified from the following.

- Monthly searches of several major healthcare databases: MEDLINE, Embase, CINAHL, PsycINFO and LILACS
- Monthly searches of trial registers: ClinicalTrials.gov and the WHO International Clinical Trials Registry Platform (which covers ClinicalTrials.gov, ISRCTN, the Chinese Clinical Trials Register, the German Clinical Trials Register, the Iranian Registry of Clinical Trials, and the Netherlands National Trials Register, plus others)
- Quarterly search of the Cochrane Library’s Central Register of Controlled Trials (CENTRAL)
- Six‐monthly searches of a number of grey literature sources from Web of Science Core Collection

Details of the search strategies used for the retrieval of reports of trials from the healthcare databases, CENTRAL and conference proceedings can be viewed in the ‘Methods used in reviews’ section within the editorial information about the Dementia and Cognitive Improvement Group: https://dementia.cochrane.org/our-trials-register. We performed additional searches in many of the sources listed above, to cover the timeframe from the last searches performed for the Register to ensure that the search for the review was as up‐to‐date and as comprehensive as possible. The most recent search was carried out on 22 December 2023.

The search strategies used are described in Appendix 1.

#### Searching other resources

We screened the reference lists of included studies for additional studies, as well as all identified review papers related to exergame interventions in people with MCI and dementia. We also contacted the corresponding authors of identified ongoing trials for additional references and unpublished data.

### Data collection and analysis

#### Selection of studies

We prepared a complete list of search results, with duplicate records removed. Two authors (AV and TP) independently screened all titles and abstracts identified from searches to determine which met the inclusion criteria. We retrieved in full text any papers identified as potentially relevant by at least one author. At this stage, we linked multiple reports of the same study. We discussed any disagreements on eligibility at this stage with a third review author (KP). Two review authors (AV and TP) independently screened full‐text articles against eligibility criteria, and resolved discrepancies by discussion with a third author to reach consensus (KP). If necessary, we contacted primary study authors to clarify study eligibility criteria. We listed as excluded studies, all potentially relevant papers that were excluded from the review at this stage, and provide reasons in the ‘Characteristics of excluded studies’ table.

We collated information from multiple reports of the same study (e.g. studies reported in more than one publication, such as study protocols, journal articles, conference abstracts) under a single study identifier; the study was the unit of interest (Li 2019). We report the screening and selection process using a PRISMA flow chart (Mohler 2009).

#### Data extraction and management

Two review authors (AV and TP) extracted data independently from included studies. They resolved any discrepancies by discussion until consensus was reached, or through consultation with a third author (KP), where necessary. We extracted data into a spreadsheet file, and later transferred and managed it in Comprehensive Meta‐Analysis 3 (Borenstein 2013) and Review Manager Web (RevMan Web 2020).

We extracted the following: study identification data (e.g. authors, year of publication, country of origin, sources of funding); details of the study (aim of intervention, study design, description of comparison group, study outcomes, settings); population (e.g. participant origin, diagnosis, age, sex/gender, education, dementia severity, and medication use); details about intervention and comparators (e.g. nature, intensity, frequency, and duration).

For dichotomous outcomes (e.g. adverse effects), we extracted the number of participants that had experienced adverse effects at each time point. For continuous outcomes, we extracted the number of participants for the intervention and control/alternative intervention groups, means, and standard deviations. We also extracted data regarding a priori moderators: type of exergame platform used: commercial versus customised; type of technology: VR‐based (e.g. head‐mounted display headsets, such as Oculus Rift or HTC Vive) versus monitor display (e.g. Wii Fit, Sports); intervention characteristics: physical activity versus physical activity with the addition of cognitive tasks; length of intervention.

One review author (AV) entered the extracted data into Comprehensive Meta‐Analysis 3 and Review Manager Web, and the second review author (TP), working independently, checked them for accuracy against the original data reported in primary studies.

We extracted the number of participants for whom the outcome was measured, as well as the mean and standard deviation (SD) for each outcome at each time point (e.g baseline, end of treatment, follow‐up) for each participant group.

As change scores were not reported, we manually calculated the change scores by subtracting the baseline measurement from the post‐intervention measurement (Higgins 2019b). We computed the standard deviations (SDs) of change scores on the assumption that the correlation between measurements at baseline and those at subsequent time points was r = 0.00, which overestimates the SD of change, but is more conservative (e.g. Bahar‐Fuchs 2019). We paid attention to the direction of the scales.

To compute change scores, we followed the procedure reported by another Cochrane review conducted on a similar topic (see Bahar‐Fuchs 2019). We first calculated change scores and SD change for all the contributing measures. Second, when two or more measures fell under the same category, we computed z‐scores for each measure by dividing the mean change by SD change. We then created a composite score. The composite score was calculated as the average of all relative z‐scores scores for each outcome of interest and SD was calculated using the =STDEV.P formula in Excel. We did this for the exergame group and then the same for the control group or groups. Then, we entered these into Review Manager Web. Additional details of measures contributing to the composite scores can be found in Table 5.

**1:** Measures used to compute composite scores for primary and secondary outcomes

**Study**	**Primary outcomes**	**Secondary outcomes domain: measures used to compute composite scores for secondary outcomes**
Arshad 2023	Global cognitive functioning	General cognition: Mini‐Mental State Exam and Montreal Cognitive Assessment
		Attention, processing speed, and working memory: Trail Making Test part A
		Executive functioning: Trail Making Test part B; Verbal Fluency Test Semantic and Phonemic
Hughes 2014	Global physical functioning	Lower limb function: Gait Speed 6‐Minute Walk Test (m/sec)
	Global cognitive functioning	General cognition: Computer Assessment of Mild Cognitive Impairment
		Attention, processing speed, and working memory: Traking task 1 (connections/sec); Traking task 2 (connections/sec)
	ADL performance	Instrumental activities of daily living: Timed Instrumental Activities of Daily Living
Karssemeijer 2019	Global physical functioning	Lower limb function: 10‐Meter Walk Test (m/sec); Timed Up & Go Test; Five Times Sit‐to‐Stand Test (sec)
		Balance: Frailty and Injuries Cooperative Studies of Interventions Technique Subtest 4
	Global cognitive functioning	Attention, processing speed, and working memory: Trail Making Test part A; Stroop Test word‐reading (sec); Stroop Test color‐naming (sec); Wechsler Adult Intelligence Scale‐III Digit Span; Wechsler Memory Scale‐III Spatial Span
		Memory: Location Learning Test Displacement score trial 1‐5; Location Learning Test Displacement score delayed recall
		Executive functioning: Trail Making Test part B; Stroop Test speed‐accuracy trade off scores; Stroop Test colour‐word interference card (sec); Stroop Test colour‐word Interference card (number of errors); Letter Fluency; Rule Shift Card Test
	ADL performance	ADL and Instrumental ADL: Katz index (Katz‐15)
Lee 2021	ADL performance	Instrumental activities of daily living: Seoul‐Instrumental Activities of Daily Living
Manser 2023	Global physical functioning	Lower limb function: Walking Speed (m/sec); Stride Duration and Length; Stance Phase Duration (% stride duration); Swing Phase Duration (% stride duration); Single Support Time (%); Double Support Time (%)
	Global cognitive functioning	General cognition: Quick Mild Cognitive Impairment Screen
		Attention, processing speed, and working memory: Digit Span PEBL forward total score; Digit Span PEBL forward max span; Digit Span PEBL backward total score; Digit Span PEBL backward max span; Trail Making Test part A—Completion Time; Trail Making Test part A—Number of Errors; Test of Attentional Performance Alertness (Condition A) (RT); Test of Attentional Performance Alertness (Condition B) (RT); Test of Attentional Performance Go‐NoGo—RT Subtest “Go‐NoGo”; Test of Attentional PerformanceGo‐NoGo—Number of Errors Subtest “Go‐NoGo”
		Memory: Subtest “logical memory” of Wechsler Memory Scale‐IV part 1 and 2 free recall; subtest “logical memory” of Wechsler Memory Scale part 2 recognition
		Executive functioning: HOTAP picture‐sorting test part A combi score; Trail Making Test part (completion time and number of errors)
		Construction and motor performance: PEBL Mental Rotation Task (RT and mental rotation task score)
Park 2018	Global cognitive functioning	Attention, processing speed, and working memory: Digit Span Forward; Digit Span Backward
		Memory: Memory‐Rey Auditory Verbal Learning Test; Memory‐Rey–Osterrieth Complex FigureTest
		Construction and motor performance: Visouspatial ability‐Wechsler Adult Intelligence Scale‐Block Design Test
		Executive functioning: Trail Making Test part B; Stroop Color‐Word Test
Ramnath 2021	Global physical functioning	Lower limb function: Timed Up & Go Test; 6‐Minute Walk Test (m)
		Balance: Dynamic Balance 10m Test (sec); Functional Reach Test (cm)
	Global cognitive functioning	General cognition: Mini‐Mental State Exam (MMSE)
		Attention, processing speed, and working memory: Stroop Total Correct Responses (%); Stroop Average RT of all Correct Responses (m.s); Stroop Correct Grey (Neutral) Words (%); Stroop Average RT of Correct Grey (Neutral) Words (m.s); Stroop Correct Colour (Incongruent) Words (%); Stroop Average RT of Correct Colour (Incongruent) Words (m.s); Stroop Average RT of total number of mistakes (m.s); Stroop Average RT of Incorrect Colour (Incongruent) Words (m.s); Stroop Average RT of Incorrect Grey (Neutral) Words (m.s); N‐back 0‐Back Score (%); N‐back 1‐Back Score (%); N‐back 2‐Back Score (%)
Robert 2021	Global cognitive functioning	General cognition: Mini‐Mental State Exam (MMSE)
Swinnen 2021	Global physical functioning	Lower limb function: Gait Speed (m/sec)
	Global cognitive functioning	General cognition: Montreal Cognitive Assessment
	ADL performance	ADL: Katz ADL index (Katz‐6)
Swinnen 2023	Global physical functioning	Lower limb function: 1‐Minute Sit‐to‐Stand Test
	Global cognitive functioning	General cognition: Mini‐Mental State Exam
Torpil 2020	Global cognitive functioning	General cognition: Loewenstein Occupational Therapy Cognitive Assessment‐Geriatric

ADL: activities of daily living; m: metres; sec: seconds; cm: centimetres; m.s: milliseconds; composite scores for primary outcomes for each study were calculated with the formula with standardised difference in means and standard error, with all subsequent secondary outcomes per each primary outcome domain; composite scores for secondary outcomes were calculated using the average of all relative z‐scores scores for each measure used to assess the secondary outcome domain; RT: reaction time

For the computation of the overall composite scores (physical, cognitive and ADL), we used a different formula, with standard difference in means and standard error, which was done on Comprehensive Meta‐Analysis 3. From here, we got one standardised mean difference (SMD) and one standard error (SE) for each group of each study at each assessment point (end of treatment; follow up), and these were entered into Review Manager Web using the Contrast level (GIV Generic Inverse Variance).

#### Assessment of risk of bias in included studies

Two review authors (AV and TP) independently assessed the risk of bias in included studies using the Cochrane Risk of bias 2 (RoB 2) tool (Higgins 2019a), and assessed the effect of assignment to intervention (the "intention to treat" effect). We used the RoB 2 Excel tool available from: www.riskofbias.info/welcome/rob-2-0-tool/current-version-of-rob-2to manage our risk of bias assessments (Higgins 2019a). We assessed RoB for primary outcomes and adverse effects at the end of treatment.

Risk of bias was assessed for five individual domains: bias arising from the randomisation process, bias due to deviations from intended interventions, bias due to missing outcome data, bias in measurement of the outcome, bias in selection of the reported result and an overall bias. We also included cluster‐RCTs. RoB 2 for cluster‐RCTs includes an additional domain of “bias related to timing and recruitment of participants”, which was also assessed in the current review. We rated the studies at low, high, or having some concerns at risk of bias in each domain. We resolved any disagreements by a discussion with a third review author (KP) to reach consensus.

We planned to assess the impact of risk of bias by conducting sensitivity analyses. For this, we attempted to exclude studies with high or unclear risk of bias, and compare effect estimates with analyses in which all studies were included.

We anticipated that participants and personnel would not be blinded in the studies because exergaming interventions are difficult to blind. Another Cochrane Review that investigated the effectiveness of virtual reality for physical rehabilitation of people with stroke stated that blinding of participants and personnel was more strongly related to the type and intrinsic characteristics of the intervention and less to the study quality (Laver 2017). We expected outcome assessors to be blinded to treatment allocation, but we did not exclude studies if they were at high or unclear risk in this domain.

#### Measures of treatment effect

For measures of treatment effect for continuous measures, we calculated the standardised mean difference (SMD) (the between‐group difference in mean values divided by the pooled SD) and 95% confidence intervals (CI) if different scales were used to measure the same outcome. We used change from baseline scores.

For dichotomous outcomes (e.g. adverse events, such as experience of severe symptoms of simulator sickness, or no experience of such symptoms), we expressed the treatment effect as a risk ratio (RR) with a 95% CI.

#### Unit of analysis issues

We anticipated the following unit of analysis issues: cross‐over trial designs, studies with multiple conditions (multi‐arm trials), repeated observations on participants, and multiple measures of the same outcome using different measurement scales. We planned to resolve the issues as follows.

For cross‐over trials, we planned to use data from the first treatment period only (before cross‐over), due to the risk of carry‐over effects (Higgins 2011).

For studies with multiple conditions, we planned to combine all relevant experimental groups into a single group, and all relevant control groups into a single comparator group (Higgins 2019b; Higgins 2019c).

For our primary outcomes (global physical functioning, cognitive functioning, and ADL performance), we planned to calculate global composite scores directly from all relevant measures or tests included in each study; this included, in most cases, more than one score derived from the same test. We used Comprehensive Meta‐Analysis 3 (CMA) software to derive one SMD and one SE for each study at each assessment point (e.g. at the end of treatment and follow‐up (Borenstein 2013)). We entered these into Review Manager Web using the generic inverse variance method and obtained pooled effect estimates across studies. We used the same procedure to obtain estimates of effects for secondary outcomes.

For repeated assessments, we planned to conduct separate comparisons (a) immediately after the intervention had finished (at the end of treatment) and (b) at follow‐up. As study duration and follow‐up times vary, and multiple assessments may be made within a follow‐up period, we only considered follow‐up in the short term (three months) to medium term (12 months), and used data from the latest assessment conducted during this time period.

We identified two cluster‐RCTs that were analysed as if they were individually randomised, which may overestimate the precision of the effect size for cluster RCTs (Bolland 2023; Higgins 2019c). However, as both studies had adjusted for clustering (Ramnath 2021; Robert 2021), we did not need to follow any specific procedures to avoid a unit‐of‐analysis error.

#### Dealing with missing data

We contacted study authors to obtain missing or unreported data (participant, outcome, or summary data). Any discrepancies between the number of participants who commenced and completed the study were addressed in our assessment of adherence in the study.

#### Assessment of heterogeneity

We identified significant heterogeneity using the Chi² and I² statistics. Based on the Chi² statistic, significant heterogeneity is if P < 0.10 (Deeks 2019). We used the I² statistic to estimate the level of heterogeneity: low (I² < 40%), moderate (I² = 40% to 60%), substantial (I² = 60% to 90%), considerable (I² > 90%). If we detected moderate or substantial heterogeneity (I² between 40% and 90%), we planned to investigate the sources of heterogeneity by conducting subgroup analyses.

If we detected considerable heterogeneity (I² > 90), we did not pool results; instead, we reported the results narratively for that comparison and outcome (Deeks 2019).

#### Assessment of reporting biases

For the primary outcomes, if the number of studies included in the pooled analysis was larger than 10 (Deeks 2019), we intended to assess publication bias associated with small study size by a visual inspection of the funnel plots. We planned to use the Duval and Tweedie’s trim‐and‐fill procedure (Duval 2000).

#### Data synthesis

We conducted separate comparisons for people with dementia and MCI.

Comparators were classified by type of control group and grouped into the following two categories:

- control group (i.e. inactive and active control such as no treatment, standard treatment, waiting list, or non‐specific active control);
- alternative treatment (e.g. treatment with a specific effect, such as physical activity or computerised cognitive training).

We conducted comparisons for the following time points:

- at the end of treatment (e.g. immediately post‐intervention);
- at follow‐up (e.g. up to 12 months following the end of intervention).

For each outcome of interest, depending on data availability, we performed the following comparisons.

Effects of interventions at the end of treatment for people with dementia

- Exergaming versus control
- Exergaming versus alternative treatment

Effects of interventions at the end of treatment for people with MCI

- Exergaming versus control
- Exergaming versus alternative treatment

Effects of interventions at follow‐up for people with dementia

- Exergaming versus control
- Exergaming versus alternative treatment

Effects of interventions at follow‐up for people with MCI

- Exergaming versus control
- Exergaming versus alternative treatment

We decided whether it was appropriate to pool data for each outcome based on a qualitative assessment of the similarity of the included studies in terms of participants, settings, intervention, comparison, and outcome measures. Because we anticipated variability in the interventions (e.g. various platforms of exergaming) or participants (e.g. dementia severity), we intended to use a random‐effects model for meta‐analysis (Deeks 2019). If we were able to pool data from at least two studies, we pooled results in a meta‐analysis in RevMan Web (Review Manager 2020), as well as in Comprehensive Meta‐Analysis 3 (Borenstein 2013). Where only two studies were available for meta‐analysis (Neguţ 2016), we used a fixed‐effect model (Borenstein 2009); for three or more studies, we used the random‐effects model. In accordance with well‐established thresholds, we interpreted the magnitude of effects as follows: small effect (SMD = 0.20 to 0.49), moderate effect (SMD = 0.50 to 0.79), and large effect (SMD ≥ 0.80) (Cohen 1988).

#### Subgroup analysis and investigation of heterogeneity

For each outcome, we planned subgroup analyses if heterogeneity (I²) was higher than 40%, and there were at least three studies per subgroup. However, because we did not have enough data, we could not perform subgroup analysis. A full list of the subgroup analysis that we had planned can be found in the Appendix 2.

#### Sensitivity analysis

To assess the robustness of our results, we intended to perform sensitivity analyses for our primary outcomes, as detailed in Appendix 3. Due to lack of data, we were unable to conduct sensitivity analyses.

#### Summary of findings and assessment of the certainty of the evidence

We assessed the certainty of the evidence with the GRADE approach. In short, the algorithm for assigning GRADE levels of evidence is based on five essential domains: risk of bias, unexplained heterogeneity or inconsistency of results, indirectness of evidence, imprecision of results, high probability of publication bias. For RCTs, the GRADE assessment starts at a high level of certainty. Based on weakness in each domain, the certainty of a body of evidence is downgraded by one or two levels for each domain. Four ratings are possible: high, moderate, low, and very low, which describe the levels of certainty associated with an outcome. According to the GRADE definitions (Schünemann 2023), high certainty of evidence indicates that we are very confident that the true effect lies close to that of the estimate of the effect. Moderate certainty indicates that we are moderately confident in the effect estimate and that the true effect is likely to be close to the estimate of the effect, though there is a possibility that it is substantially different. Low certainty indicates that our confidence in the effect estimate is limited, and the true effect may be substantially different from the estimate of the effect. Further research is very likely to have an important impact on our confidence in the estimate of effect, and is likely to change the estimate. Very low certainty indicates that we have very little confidence in the effect estimate and the true effect is likely to be substantially different from the estimate of effect.

Using GRADEpro GDT software (GRADEpro GDT), we generated summary of findings tables for the following comparisons.

- Exergaming versus control at the end of treatment for people with dementia
- Exergaming versus alternative treatment at the end of treatment for people with dementia.
- Exergaming versus control at the end of treatment for people with MCI
- Exergaming versus alternative treatment at the end of treatment for people with MCI

We included the following outcomes in each summary of findings table.

- Global physical functioning
- Global cognitive functioning
- Global ADL
- Adverse effects

## Results

### Description of studies

#### Results of the search

We identified 8815 records through database searching. After excluding 2361 duplicates, we screened a total of 6454 records based on their title and abstract and rejected 5985 records. We assessed the remaining 469 records in full, and we rejected 374 records by applying our eligibility criteria. Twenty‐seven records are ongoing studies and 14 are awaiting classification. A list of the 34 studies we excluded with reasons can be found in the Characteristics of excluded studies table.

We included 11 studies (19 records) in the qualitative and quantitative synthesis (meta‐analysis). We contacted the authors of protocols and ongoing trials identified through clinical trials registers to ask for published studies or any information concerning the location of available data or to ask for additional information to help us apply eligibility criteria. We contacted each author twice.

#### Included studies

We identified 11 studies reported in 20 records that were eligible for this review. For Karssemeijer 2019, we identified four records: two articles published in peer‐reviewed journals, one conference abstract, and one poster (with additional information obtained from the author). For Manser 2023, we included one published study protocol, one registration in a clinical trial registry and one paper published in a peer‐reviewed journal. The remaining seven studies were all articles published in peer‐reviewed journals. Five studies either had a published protocol (Karssemeijer 2019) or were registered in a clinical trial registry (Park 2018; Ramnath 2021; Swinnen 2021; Swinnen 2023). The remaining five studies did not mention a protocol, and we assumed they were not registered (Arshad 2023; Hughes 2014; Lee 2021; Robert 2021; Torpil 2020). Three studies received no financial support (Park 2018; Ramnath 2021; Torpil 2020), six received grant funding (Hughes 2014; Karssemeijer 2019; Manser 2023Robert 2021; Swinnen 2021; Swinnen 2023), and two did not specify whether they received funding (Arshad 2023; Lee 2021).

All 11 studies were published in English between 2014 and 2023, with four published in 2021 and three in 2023. Two studies were conducted in Belgium (Swinnen 2021; Swinnen 2023) and two in the Republic of Korea (Lee 2021; Park 2018). One study was conducted in France (Robert 2021), one in the Netherlands (Karssemeijer 2019), one in Pakistan (Arshad 2023), one in South Africa (Ramnath 2021), one in Switzerland (Manser 2023), one in Turkey (Torpil 2020) and one in the USA (Hughes 2014). Nine studies were individually‐randomised RCTs (Arshad 2023; Hughes 2014; Karssemeijer 2019; Lee 2021; Manser 2023; Park 2018; Swinnen 2021; Swinnen 2023; Torpil 2020), and two were cluster‐RCTs, which randomised groups of participants from various settings such as retirement homes, memory centres, day care and nursing homes (Ramnath 2021; Robert 2021). Sample sizes ranged from 15 participants (Manser 2023) to 78 participants (Park 2018).

A summary of included studies characteristics can be found in Table 6, and further details can be found in the Characteristics of included studies table.

**2:** Summary of participants, treatment, comparators

**Study**	**Participant diagnosis**	**Nature of the exergame intervention**	**Nature of the control or alternative intervention**	**Number, frequency and duration of sessions**
Arshad 2023	MCI	Exergames were played using Microsoft XBox.	Physical exercise such as stretching and strength training (alternative treatment)	Participants trained for 30 minutes five times per week for 6 weeks.
Hughes 2014	MCI	Exergames were played using Nintendo Wii.	Healthy ageing education (active control)	Participants met for 90 minutes once per week for 24 weeks.
Karssemeijer 2019	Dementia	Exergames consisted of a combined cognitive–aerobic bicycle training.	Aerobic training (alternative treatment) and relaxation and flexibility exercises (active control)	Participants met for approximately 30 minutes three times per week for 12 weeks.
Lee 2021	MCI	Exergames were played with Nintendo Wii.	Conventional elderly care (inactive control)	Participants met for 15 minutes three times per week for 12 weeks.
Manser 2023	MCI	Exergames were played at home using the “Senso Flex”, a home‐based version of the “Senso” platform (Dividad software).	Standard care (inactive control)	Participants were instructed to train for about 21 minutes five times per week for 12 weeks.
Park 2018	MCI	Exergames were played using the Nintendo Wii.	Cognition‐specific computer training (alternative treatment)	Participants met for 10 minutes three times per week for 10 weeks.
Ramnath 2021	MCI	Exergames were played using Microsoft XBox.	Conventional multimodal (alternative treatment)	Participants met for 30 minutes two times per week for 12 weeks.
Robert 2021	Dementia MCI	Exergames were delivered via X‐Torp game.	Standard care (inactive control)	Participants met for 15 minutes two times per week for 12 weeks.
Swinnen 2021	Dementia	Exergames were delivered using the device “Dividat Senso”.	Listening to favourite music (active control)	Participants met for 15 minutes three times per week for 8 weeks.
Swinnen 2023	Dementia	Exergames were delivered with the VITAAL exergame prototype, specially designed for the rehabilitation of older adults.	Physical exercise such as walking and squatting and stepping exercises (alternative treatment)	Participants took part in three individual sessions per week for 12 weeks, with 30 minutes per session.
Torpil 2020	MCI	Exergames were played using the Microsoft XBox.	Cognition‐specific computer training (alternative treatment)	Participants met for 45 minutes two times per week for 12 weeks.

#### General characteristics of participants

Participant source varied and included clinics (e.g. occupational therapy department; Torpil 2020), clinics and long‐term care facilities (Arshad 2023; Lee 2021; Manser 2023; Swinnen 2021; Swinnen 2023), retirement homes (Ramnath 2021), community (Hughes 2014), community welfare centres (Park 2018), day care, memory centre, and nursing homes (Robert 2021), memory clinics, day care centres, advertisements in local newspapers and word of mouth (Karssemeijer 2019).

Seven studies included people with MCI only (Arshad 2023; Hughes 2014; Lee 2021; Manser 2023; Park 2018; Ramnath 2021; Torpil 2020), three studies included people with dementia only (Karssemeijer 2019; Swinnen 2021; Swinnen 2023), and one study included both people with MCI and people with dementia (Robert 2021). Additional data were obtained through correspondence with study authors (Arshad 2023; Robert 2021). In six studies, people with MCI (Arshad 2023; Hughes 2014; Lee 2021; Park 2018; Ramnath 2021; Torpil 2020) were diagnosed according to established criteria (Petersen 1999), one study used the DSM 5 criteria for mild neurocognitive disorder (Robert 2021), and one study included participants with MCI diagnosed with either DSM 5, ICD 11 or adapted Petersen criteria (Manser 2023). In all four studies, which included people with dementia, the diagnosis was based on DSM IV and DSM 5 criteria. Dementia subtypes were Alzheimer's disease (AD), vascular dementia (VD), mixed AD and VD, neurocognitive disorder not otherwise specified (Karssemeijer 2019; Swinnen 2021; Swinnen 2023), and one study did not specify the type of dementia (Robert 2021). Three studies included participants with a moderate level of dementia severity (MMSE M = 17.51, SD = 4.01, Swinnen 2021; MMSE M = 17.2, SD = 5.48, Swinnen 2023; MMSE M = 18.30, SD = 3.32, Robert 2021) and one study included participants with mild dementia with a MMSE mean of 22.16 (SD = 3.12) (Karssemeijer 2019). None of the studies which included people with MCI specified the type of MCI (e.g. amnestic).

In two studies, the mean age of participants was between 60 and 70 years (Arshad 2023; Park 2018), in five studies the mean age was between 70 and 80 years (Hughes 2014; Karssemeijer 2019; Lee 2021; Manser 2023; Ramnath 2021; Torpil 2020) and in two studies between 80 and 90 years (Robert 2021; Swinnen 2021; Swinnen 2023). Four studies reported the percentage of participants with a university degree between 18% to 37% (Arshad 2023; Karssemeijer 2019; Ramnath 2021; Torpil 2020) and three studies reported average years of education between 8 years to 14 years (Hughes 2014; Manser 2023Park 2018; Robert 2021), while two studies did not report any data on education (Lee 2021; Swinnen 2021; Swinnen 2023).

#### General characteristics of experimental interventions

All studies included one condition that met our criteria for an exergame intervention. All exergame interventions used a monitor display and none used a VR‐based technology platform (HMD display). Five studies included commercially available exergame platforms: two used the Microsoft Xbox (Ramnath 2021; Torpil 2020), three used Nintendo Wii (Hughes 2014; Lee 2021; Park 2018) and five used customised exergames (Karssemeijer 2019; Manser 2023Robert 2021; Swinnen 2021; Swinnen 2023). Five exergame interventions focused only on physical activity (Hughes 2014; Lee 2021; Park 2018; Ramnath 2021; Torpil 2020) while five studies included exergames with a combination of physical activity and cognitive training (Karssemeijer 2019; Manser 2023; Robert 2021; Swinnen 2021; Swinnen 2023). In most of the reviews there was no explicit mention of whether the interventions were co‐designed with participants or healthcare professionals. However, we have reasons to believe that those that used customised exergames used co‐design during the development phase of the intervention (Karssemeijer 2019; Manser 2023; Robert 2021; Swinnen 2021; Swinnen 2023).

Multiple intervention settings were used: a church (Hughes 2014), community centres (Karssemeijer 2019), day care, memory centres, nursing homes (Robert 2021), clinics and long‐term care facilities (Swinnen 2021; Swinnen 2023), retirement homes (Ramnath 2021), participants' home (Manser 2023), four studies did not report the intervention settings (Arshad 2023; Lee 2021; Park 2018; Torpil 2020). The same four studies did not report whether the intervention was provided to individuals or in groups (Arshad 2023; Lee 2021; Park 2018; Torpil 2020). Of the other seven studies, four reported using individual sessions (Karssemeijer 2019; Manser 2023; Swinnen 2021; Swinnen 2023), two group sessions (Hughes 2014; Ramnath 2021), and one reported mixed sessions (Robert 2021).

#### General characteristics of comparison conditions

Ten studies had one control group and one study had two control groups (Karssemeijer 2019). Three control groups were inactive control groups in the form of conventional care (Lee 2021; Manser 2023; Robert 2021), and three were active control groups: relaxation exercises (Karssemeijer 2019), healthy ageing education programme (Hughes 2014); listening to favourite music videos (Swinnen 2021). The other five control groups were in the form of alternative treatment: aerobic exercise (Karssemeijer 2019), computerised cognitive training and rehabilitation (Park 2018; Torpil 2020), low intensity conventional multimodal exercises (Ramnath 2021), and exercise therapy (Arshad 2023; Swinnen 2023). The control interventions were delivered in the same settings as the exergame interventions and to individuals or groups in the same way.

#### Outcomes

All the studies used previously validated scales and instruments for the measurement of primary and secondary outcomes. Physical functioning was assessed in six studies (Hughes 2014; Karssemeijer 2019; Manser 2023; Ramnath 2021; Swinnen 2021; Swinnen 2023). Cognitive functioning was measured in nine studies (Hughes 2014; Karssemeijer 2019; Manser 2023; Park 2018; Ramnath 2021; Robert 2021; Swinnen 2021; Swinnen 2023; Torpil 2020). Four studies evaluated ADL performance (Hughes 2014; Karssemeijer 2019; Lee 2021; Swinnen 2021). Secondary outcomes were included as follows: QoL in four studies (Park 2018; Manser 2023; Swinnen 2021; Swinnen 2023), physical performance in two studies (Karssemeijer 2019; Swinnen 2021; Swinnen 2023), and physical activity and frailty in one study (Karssemeijer 2019).

Ten studies compared groups at the end of treatment (Arshad 2023; Hughes 2014; Karssemeijer 2019; Lee 2021; Manser 2023; Park 2018; Ramnath 2021; Swinnen 2021; Swinnen 2023; Torpil 2020). Three studies conducted follow‐up assessments (Hughes 2014; Karssemeijer 2019; Robert 2021). The average follow‐up time was 32 weeks after the end of treatment, ranging from 24 weeks (Karssemeijer 2019; Robert 2021) to 48 weeks (Hughes 2014).

Five studies reported that no participant experienced adverse effects related to the intervention or control conditions (Karssemeijer 2019; Manser 2023; Park 2018; Ramnath 2021; Swinnen 2021), and one study noted that two participants reported adverse effects: one participant in the exergaming group reported feeling faint and one participant in the same exergaming group reported knee pain while performing the exergaming exercises (Swinnen 2023). In all six studies, the participants were supervised by healthcare professionals during the sessions. The remaining studies did not mention any information concerning adverse effects (Arshad 2023; Hughes 2014; Lee 2021; Robert 2021; Torpil 2020).

Four studies reported data on falls. However, in two of them there was no distinction made between falls that occurred during the intervention and falls outwith the intervention. Karssemeijer 2019 reported that one participant dropped out from the active control group after allocation due to a fracture after a fall, while Ramnath 2021 reported that no falls occurred. One study reported three falls involving two participants that took place at participants' homes and were not directly related to the exergaming intervention, although the participants were in the exergame group (Manser 2023). Swinnen 2023 mentioned two falls. One participant in the alternative treatment condition fell when returning home from the treatment and one participant from the exergame condition fell during the weekend, which was not directly related to the intervention.

Three studies reported data on satisfaction (Hughes 2014) or acceptance and motivation (Manser 2023; Swinnen 2023). Two studies assessed usability and user experience (Manser 2023; Swinnen 2023). Seven studies included data on attendance and adherence (Hughes 2014; Karssemeijer 2019; Manser 2023; Ramnath 2021; Robert 2021; Swinnen 2021; Swinnen 2023). There were no dropouts at the end of treatment in four studies (Hughes 2014; Lee 2021; Park 2018; Ramnath 2021), and two studies had more than 10 participants drop out: 10/55 (Swinnen 2021) and 18/115 (Karssemeijer 2019). Three studies conducted follow‐up assessments, with 11/115 (Karssemeijer 2019), 21/90 (Robert 2021) and 1/20 dropping out (Hughes 2014). All studies gave reasons for study withdrawal. No caregiver outcomes were reported.

#### Intervention dose and duration

The exergame interventions lasted on average for 961 minutes, ranging from 360 (Swinnen 2021) to 2160 minutes (Hughes 2014). Each session lasted on average for 33 minutes, ranging from 15 (Lee 2021; Swinnen 2021) to 90 minutes (Hughes 2014). In total, an average of 30 intervention sessions took place, ranging from 24 (Hughes 2014; Ramnath 2021; Robert 2021; Swinnen 2021; Torpil 2020) to 54 sessions (Manser 2023). On average, four sessions per week were reported, ranging from one (Hughes 2014) to five sessions (Arshad 2023; Park 2018).

The average total duration of the control intervention excluding inactive control groups was 953 minutes, ranging from 360 (Swinnen 2021) to 2160 minutes (Hughes 2014). Each session lasted on average for 38 minutes, ranging from 15 (Lee 2021; Swinnen 2021) to 90 minutes (Hughes 2014). In total, an average of 29 sessions took place, ranging from 24 (Hughes 2014; Ramnath 2021; Swinnen 2021; Torpil 2020) to 36 sessions (Karssemeijer 2019). On average, four sessions per week were reported, ranging from one (Hughes 2014) to five sessions (Arshad 2023; Manser 2023; Park 2018).

Other than studies that included inactive control groups (Lee 2021; Manser 2023; Robert 2021), all studies involved intervention and control groups that were matched in terms of their dose. For example, the session length, number of sessions per week, and number of weeks were identical for the exergame and control groups.

#### Funding and conflicts of interest

Six studies reported one or more sources of funding or financial support, and all were externally funded to various extents via public, commercial, or not‐for‐profit sectors (Hughes 2014; Karssemeijer 2019; Manser 2023; Robert 2021; Swinnen 2021; Swinnen 2023). Three studies stated explicitly that they received no specific funding or financial support (Park 2018; Ramnath 2021; Torpil 2020). Two studies did not provide information on funding (Arshad 2023; Lee 2021).

Seven studies declared no conflict of interest (Arshad 2023; Hughes 2014; Manser 2023; Park 2018; Ramnath 2021; Torpil 2020; Swinnen 2023), and one study did not provide any statement on this (Lee 2021). Three studies reported conflicts of interest. One co‐author of the Swinnen 2021 study declared that they were a co‐funder of the spin‐off company that developed the exergame and an external advisor. However, the author mentioned that neither they nor the affiliated institution received any revenue. One co‐author in the Robert 2021 study was an employee of the company that developed the exergame. The corresponding author from the Karssemeijer 2019 study declared that he is co‐author of the Location Learning Test—Revised and receives royalties from its publisher.

#### Excluded studies

We assessed 469 papers in full‐text. After applying the eligibility criteria, we excluded 449 papers (see Figure 1). Most of these were clearly ineligible. For 34 studies that were less clear, we recorded details of our reasons for exclusion in the Characteristics of excluded studies table. Of these, nine studies evaluated an intervention that was not an exergame, and four involved a population other than people with MCI or dementia. We excluded one study that was not an RCT, one that did not measure any outcomes of interest, and two that did not provide data on request. We excluded 17 studies because the diagnosis of MCI was not based on established criteria as specified in the eligibility criteria and was mainly based on cut‐off scores from MoCA and MMSE. In cases where the information in the paper was not sufficient (e.g. authors only described the sample as being people with MCI but did not detail how this diagnosis was made), we sought additional information concerning the diagnosis from the authors. As per our protocol, in cases where the authors did not respond to clarify this, we were conservative and excluded the study because it did not fit our criteria related to participant selection. Additionally, in the case of protocols and clinical trial registries, we contacted the authors to ask for a published study and when they either did not respond or did not provide any data or papers, we categorised the records as ongoing (N = 27) or awaiting classification (N = 14).

**1:**
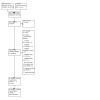


### Risk of bias in included studies

We assessed the risk of bias in the 11 studies included in the review using the risk of bias tool RoB 2 (Higgins 2019a; Sterne 2019). We assessed the risk of bias for our primary outcomes at the end of treatment. Our judgements and support for judgements can be found in the Characteristics of included studies and in the forest plots (Data and analyses). Based on the criteria from the Rob 2 tool, we categorised studies as being at low risk of bias (green (+)), high risk of bias (red (‐)), or having some concerns (yellow (?)). Our assessments were conducted for five domains: bias arising from the randomisation process, bias due to deviations from intended interventions, bias due to missing outcome data, bias in measurement of the outcome, bias in selection of the reported result; we also made a judgement about the overall risk of bias. We assessed an additional domain for two cluster‐RCTs: bias related to timing and recruitment of participants (Ramnath 2021; Robert 2021). Excel files that detail the RoB assessments can be found here: https://doi.org/10.17605/OSF.IO/96Q7M.

We judged the overall risk of bias for primary outcomes for all studies to be either high or 'some concerns'. None of the studies were at low risk of bias overall. In the case of individual risk of bias domains, most of the studies were judged to have some concerns about the randomisation process, mostly because the study authors did not describe in detail how randomisation was performed or did not provide information on allocation concealment, or both. A few studies did not provide enough data to properly assess baseline differences between groups. We had some concerns about bias in all studies due to deviations from intended interventions because blinding of participants and people delivering the interventions is not possible for this type of intervention, and because most studies did not report any information concerning deviations from planned interventions or whether any analyses to account for this were carried out. Similarly, for the bias in measurement of the outcome domain, in some cases, the study authors did not blind outcome assessors, and they used measures that could be impacted by this lack of blinding, e.g. cognitive tests or physical functioning tests such as sit‐to‐stand test or gait speed. In terms of bias due to missing outcome data, most studies did not have any dropouts or conduct a sensitivity analysis, so we judged them to be at low risk of bias for this domain. Risk of bias tables can be found in the supplementary material attached to this review: Table 55; Table 56; Table 57; Table 58; Table 59; Table 60; Table 61; Table 62; Table 63.

### Effects of interventions

See: Table 1; Table 2; Table 3; Table 4

For our primary outcomes and adverse effects at the end of treatment, we created summary of findings tables. See Table 1; Table 2; Table 3; Table 4.

Across all comparisons, there were insufficient data and studies available to conduct our planned subgroup analyses, based on severity of dementia, intervention characteristics, type of control intervention, type of exergame platform used, type of technology, length of intervention, and length of follow‐up period (see Assessment of heterogeneity; Subgroup analysis and investigation of heterogeneity; Appendix 2).

For secondary outcomes, we pooled results if we had sufficient data or if heterogeneity was low. If not, we presented results narratively.

#### Effects of interventions at the end of treatment for people with dementia

##### Exergaming versus control

###### Primary outcomes

Two studies included data on physical, cognitive, and activities of daily living (ADL) outcomes at the end of treatment for people with dementia (Karssemeijer 2019; Swinnen 2021).

Relative to a control group (i.e. active control such as relaxation and flexibility exercises or listening to favourite music), we found a large effect favouring exergaming for global cognitive functioning and little to no difference between groups in global physical functioning and ADL.

####### Global physical functioning

We found little to no difference between groups in global physical functioning (SMD ‐0.20, 95% ‐0.57 to 0.17, 2 studies, 113 participants, very low certainty of evidence). Our certainty about the evidence was very low because of serious concerns about high risk of bias, inconsistency and imprecision. See Analysis 1.1 and Table 1.

####### Global cognitive functioning

We found a large positive effect on global cognitive functioning (SMD 1.47, 95% 1.04 to 1.90, 2 studies, 113 participants, very low certainty of evidence). Our certainty was very low because of serious concerns about high risk of bias, inconsistency, and imprecision. See Analysis 1.2 and Table 1.

####### Activities of daily living

We found little to no difference between groups for ADL (SMD ‐0.28, 95% ‐0.65 to 0.09, 2 studies, 113 participants, very low certainty of evidence). Our certainty was very low because of serious concerns about the high risk of bias and imprecision. See Analysis 1.3 and Table 1.

###### Secondary outcomes

See Table 7 for full narrative results.

**3:** Secondary outcomes. Exergaming vs control at the end of treatment for people with dementia

**Secondary outcome domain**	**Study**	**N**	**Follow‐up (weeks)**	**Comparison**	**Measure(s)**	**Scale metrics**	**Numerical data**	**Narrative interpretation**
Lower limb function	Karssemeijer 2019	68	12	12 weeks of exergaming versus relaxation and flexibility exercises (control)	10‐Meter Walk Test (10‐M WT)	Walking speed in metres/seconds; higher scores reflect better performance	Post‐treatment mean (SD) of exergame vs control: 1. 10‐M WT = 1.12 (0.37) vs 1.01 (0.31); 2. TUG = 13.0 (4.2) vs 15.0 (7.5); 3. 5TSST = 14.7 (3.9) vs 16.3 (7.7) No between‐group differences (all Ps > 0.05)	Mixed findings
					Timed Up & Go Test (TUG)	Time to complete the test; lower scores reflect better performance		
					Five Times Sit‐to‐Stand Test (5T‐STS)	Time to complete the test; lower scores reflect better performance		
	Swinnen 2021	45	8	8 weeks of exergaming versus listening to favourite music (control)	4‐Meter Walk Test (4‐M WT)	Gait speed in metres/second; higher scores reflect better performance	Post‐treatment mean (SD) of exergame vs control: 1. 4‐M WT = 0.8 (0.3) vs 0.5 (0.2), P < 0.001	
Balance	Karssemeijer 2019	68	12	12 weeks of exergaming versus relaxation and flexibility exercises (control)	Frailty and Injuries Cooperative Studies of Interventions Technique Subtest 4 (FICSIT‐4 score)	Range 0 to 5; higher scores reflect better performance	Post‐treatment mean (SD) of exergame vs control: 1. FICSIT‐4 = 3.8 (1.1) vs 3.7 (1.3), P > 0.05	Little to no difference between groups
General cognition	Swinnen 2021	45	8	8 weeks of exergaming versus listening to favourite music (control)	Montreal Cognitive Assessment (MoCA)	Range 0 to 30; higher scores reflect better performance	Post‐treatment mean (SD) of exergame vs control: 1. MoCA = 12.1 (5.2) vs 5.7 (4.0), P < 0.001	Intervention had a beneficial effect
Attention, processing speed, and working memory	Karssemeijer 2019	68	12	12 weeks of exergaming versus relaxation and flexibility exercises (control)	Trail Making Test part A (TMT‐A)	Time taken to complete the task; lower scores reflect better performance	Post‐treatment mean (SD) of exergame vs control: 1. TMT‐A = 50.3 (42.0) vs 63.6 (61.0); 2. Stroop Test word‐reading = 37.3 (10.3) vs 43.3 (22.6); 3. Stroop Test color‐naming = 49.4 (17.9) vs 56.7 (25.9); 4. WAIS‐III Digit Span = 10.1 (2.9) vs 10.8 (4.0); 5. WMS‐III Spatial Span = 8.3 (3.6) vs 8.7 (3.6) Mean difference for processing speed (TMT‐A, Stroop Test color‐naming and word‐reading) between exergame and control at the end of treatment: 0.326, 95% CI 0.081 to 0.571, P = 0.009 The remaining comparisons were not statistically significant.	Mixed findings
					Stroop Test word‐reading	Time taken to complete the task; lower scores reflect better performance		
					Stroop Test color‐naming	Time taken to complete the task; lower scores reflect better performance		
					Wechsler Adult Intelligence Scale‐III Digit Span	Number of digits correctly repeated; higher scores reflect better performance		
					Wechsler Memory Scale‐III Spatial Span	Number of digits correctly repeated; higher scores reflect better performance		
Memory	Karssemeijer 2019	68	12	12 weeks of exergaming versus relaxation and flexibility exercises (control)	Location Learning Test Displacement score trial 1‐5 (LLT 1‐5)	Sum of errors; lower scores reflect better performance	Post‐treatment mean (SD) of exergame vs control: 1. LLT 1 to 5 = 85.5 (41.7) vs 107.1 (41.7), P > 0.05 2. 6. LLT delayed recall = 16.9 (11.2) vs 20.0 (10.9), P > 0.05	Little to no difference between groups
					Location Learning Test Displacement score delayed recall (LLT delayed recall)	Sum of errors; lower scores reflect better performance		
Executive functioning	Karssemeijer 2019	68	12	12 weeks of exergaming versus relaxation and flexibility exercises (control)	Trail Making Test part B (TMT‐B)	Time taken to complete the task; lower scores reflect better performance	Post‐treatment mean (SD) of exergame vs control: 1. TMT‐B = 160.7 (106.2) vs 170.1 (106.8) 2. Stroop Test speed‐accuracy trade off scores = 0.37 (0.18) vs 0.31 (0.18); 3. Stroop Test colour‐word interference card = 126.9 (63.7) vs 152.5 (81.3); 4. Stroop Test colour‐word Interference card (number of errors) = 8.0 (12.8) vs 10.7 (13.5); 5. Letter Fluency = 20.9 (10.6) vs 20.2 (12.0); 6. Rule Shift Card Test = 8.3 (6.4) vs 8.8 (6.2) None of the comparisons were statistically significant.	Little to no difference between groups
					Stroop Test speed‐accuracy trade off scores	Speed‐accuracy trade‐off scores are calculated with the formula: (100*accuracy) / reaction time; lower scores reflect better performance		
					Stroop Test colour‐word interference card	Time taken to complete the task; lower scores reflect better performance		
					Stroop Test colour‐word Interference card (number of errors)	Number of errors; lower scores reflect better performance		
					Letter Fluency	Number of correct words generated; higher scores reflect better performance		
					Rule Shift Card Test	Number of errors; lower scores reflect better performance		
Functional status and physical performance	Karssemeijer 2019	68	12	12 weeks of exergaming versus relaxation and flexibility exercises (control)	Short Physical Performance Battery Test (SPPB)	Scores range from 0 (worst performance) to 12 (best performance); higher scores reflect better performance	Post‐treatment mean (SD) of exergame vs control: 1. SPPB = 9.5 (1.8) vs 9.2 (2.4), P > 0.05 Post‐treatment mean (SD) of exergame vs control: 1. SPPB = 8.5 (2.5) vs 3.8 (2.5), P < 0.001	Mixed findings
	Swinnen 2021	45	8	8 weeks of exergaming versus listening to favourite music (control)	Short Physical Performance Battery Test (SPPB)	Scores range from 0 (worst performance) to 12 (best performance); higher scores reflect better performance		
Physical activity	Karssemeijer 2019	68	12	12 weeks of exergaming versus relaxation and flexibility exercises (control)	Physical Activity Scale for the Elderly (PASE)	PASE score ranges from 0 to 400 or more; higher scores reflect higher levels of physical activity	Post‐treatment mean (SD) of exergame vs control: 1. PASE = 78.6 (60.1) vs 55.1 (44.2), P > 0.05	Little to no difference between groups
Frailty	Karssemeijer 2019	68	12	12 weeks of exergaming versus relaxation and flexibility exercises (control)	Evaluative Frailty Index for Physical Activity (EFIP)	Scores range from 0.00 to 1.00; lower scores reflect better performance	Post‐treatment mean (SD) of exergame vs control: 1. EFIP = 0.23 (0.12) vs 0.23 (0.12) Mean difference between exergame and control at the end of treatment: ‐0.034, 95% CI ‐0.062 to ‐0.007, P = 0.012	Mixed findings
Quality of life	Swinnen 2021	45	8	8 weeks of exergaming versus listening to favourite music (control)	Dementia Quality of Life (DQoL)	Scores range from 1 to 5; higher scores reflect better QoL	Post‐treatment mean (SD) of exergame vs control: 1. DQoL = 3.5 (0.9) vs 3.0 (0.9), P = 0.012; not statistically significant, as applied multiple comparison corrections significant if P < 0.00625	Little to no difference between groups

P: probability; SD: standard deviation; vs: versus

We found little or no difference in balance between exergaming and control groups of people with dementia at the end of treatment. For lower limb function, there were mixed findings.

For general cognition, the intervention had a beneficial effect, but for attention, processing speed, and working memory, findings were mixed. For memory and executive functioning, there was little to no difference between groups.

For functional status and physical performance, as well as frailty, findings were mixed.

For physical activity and quality of life, there was little to no difference between groups.

##### Exergaming versus alternative treatment

###### Primary outcomes

Two studies included data on physical and cognitive functioning at the end of treatment for people with dementia (Karssemeijer 2019; Swinnen 2023), but only Karssemeijer 2019 included ADL outcomes as well.

Relative to alternative treatment (i.e. treatment with specific effects, such as aerobic exercises or cycling on a stationary bike), we found little to no difference in global physical, cognitive, and ADL outcomes.

####### Global physical functioning

We found little to no difference between groups in global physical functioning (SMD 0.14, 95% ‐0.30 to 0.58; 2 studies, 85 participants; very low certainty of evidence). Our certainty was very low because of serious concerns about a high risk of bias and imprecision. See Analysis 2.1 and Table 2.

####### Global cognitive functioning

We found little to no difference between groups in global cognitive functioning (SMD 0.11, 95% ‐0.33 to 0.55; 2 studies, 85 participants; very low certainty of evidence). Our certainty was very low because of serious concerns about high risk of bias, inconsistency, and imprecision. See Analysis 2.3 and Table 2.

####### Activities of daily living

Only one study included ADL outcomes (Karssemeijer 2019). Results showed little to no difference in ADL between groups (n = 67). Our certainty was low because of serious concerns about the risk of bias and imprecision. Table 8 provides numerical data, outcome definitions used within the study, a brief narrative interpretation, and the overall risk of bias. See Table 2.

**4:** Analysis 2.3 Exergaming versus alternative treatment at the end of treatment for people with dementia: change in ADL

**Study**	**N**	**Follow‐up (weeks)**	**Comparison**	**ADL domain: measure(s)**	**Scale metrics**	**Numerical data**	**Narrative interpretation**	**Risk of bias**
Karssemeijer 2019	67	12	12 weeks of exergaming versus aerobic exercise (alternative treatment)	Katz index (Katz‐15)	Range 0 to 15; higher scores reflect better performance	Mean (SD) of exergame vs alternative treatment and P value of difference between groups: 1. Katz index = 4.9 (3.3) vs 5 (3.4), P = 0.12	Little or no difference between groups	Overall risk of bias: high. Comment: There are some or high concerns in most domains.

ADL: activities of daily living; N: number; P: probability; SD: standard deviation; vs: versus

###### Secondary outcomes

We pooled results for lower limb functioning and for functional status and physical performance. For the remaining secondary outcomes, narrative results are presented in Table 9.

**5:** Secondary outcomes. Exergaming vs alternative treatment at the end of treatment for people with dementia

**Secondary outcome domain**	**Study**	**N**	**Follow‐up (weeks)**	**Comparison**	**Measure(s)**	**Scale metrics**	**Numerical data**	**Narrative interpretation**
Balance	Karssemeijer 2019	67	12	12 weeks of exergaming vs aerobic (alternative treatment)	Frailty and Injuries Cooperative Studies of Interventions Technique Subtest 4 (FICSIT‐4 score)	The score ranges from 0 to 5; higher scores reflect better performance	Post‐treatment mean (SD) of exergame vs control: 1. FICSIT‐4 = 3.8 (1.1) vs 3.4 (1.3), P > 0.05	Little to no difference between groups
General cognition	Swinnen 2023	18	12	12 weeks of exergaming vs exercise therapy (alternative treatment)	Mini‐Mental State Examination (MMSE)	Range 0 to 30; higher scores reflect better performance	Post‐treatment mean (SD) of exergame vs control: 1. MMSE = 17.1 (7.2) vs 15.7 (4.9), P = 0.012	Intervention had a beneficial effect
Attention, processing speed, and working memory	Karssemeijer 2019	67	12	12 weeks of exergaming vs aerobic (alternative treatment)	Trail Making Test part A (TMT‐A)	Time taken to complete the task; lower scores reflect better performance	Post‐treatment mean (SD) of exergame vs control: 1. TMT‐A = 50.3 (42.0) vs 37.7 (26.9) 2. Stroop Test word‐reading = 37.3 (10.3) vs 33.8 (8.5); 3. Stroop Test color‐naming = 49.4 (17.9) vs 42.8 (9.9); 4. WAIS‐III Digit Span = 10.1 (2.9) vs 10.8 (2.8); 5. WMS‐III Spatial Span = 8.3 (3.6) vs 9.5 (3.2) Mean difference processing speed (Z score TMT‐A, Stroop Test color‐naming and word‐reading) between exergame and control at the end of treatment: 0.37, 95% CI 0.103 to 0.637, P = 0.007 The remaining comparisons were not statistically significant.	Mixed findings
					Stroop Test word‐reading	Time taken to complete the task; lower scores reflect better performance		
					Stroop Test color‐naming	Time taken to complete the task; lower scores reflect better performance		
					Wechsler Adult Intelligence Scale‐III Digit Span (WAIS‐III Digit Span)	Number of digits correctly repeated; higher scores reflect better performance		
					Wechsler Memory Scale‐III Spatial Span (WMS Digit Span)	Number of digits correctly repeated; higher scores reflect better performance		
Memory	Karssemeijer 2019	67	12	12 weeks of exergaming vs aerobic (alternative treatment)	Location Learning Test Displacement score trial 1‐5 (LLT 1‐5)	Sum of errors; lower scores reflect better performance	Post‐treatment mean (SD) of exergame vs control: 1. LLT 1‐5 = 85.5 (41.7) vs 96.6 (41.1), P > 0.05; 2. 6. LLT delayed recall = 16.9 (11.2) vs 19.1 (9.9), P > 0.05	Little to no difference between groups
					Location Learning Test Displacement score delayed recall (LLT delayed recall)	Sum of errors; lower scores reflect better performance		
Executive functioning	Karssemeijer 2019	67	12	12 weeks of exergaming vs aerobic (alternative treatment)	Trail Making Test part B (TMT‐B)	Time taken to complete the task; lower scores reflect better performance	Post‐treatment mean (SD) of exergame vs control: 1. TMT‐B = 160.7 (106.2) vs 124.9 (97.9) 2. Stroop Test speed‐accuracy trade off scores = 0.37 (0.18) vs 0.37 (0.18); 3. Stroop Test colour‐word interference card = 126.9 (63.7) vs 121.6 (61.7); 4. Stroop Test colour‐word Interference card (number of errors) = 8.0 (12.8) vs 6.0 (8.3); 5. Letter Fluency = 20.9 (10.6) vs 22.9 (9.5); 6. Rule Shift Card Test = 8.3 (6.4) vs 8.3 (5.6) None of the comparisons were statistically significant.	Little to no difference between groups
					Stroop Test speed‐accuracy trade off scores	Speed‐accuracy trade‐off scores are calculated with the formula: (100*accuracy) / reaction time; lower scores reflect better performance		
					Stroop Test colour‐word interference card	Time taken to complete the task; lower scores reflect better performance		
					Stroop Test colour‐word Interference card (number of errors)	Number of errors; lower scores reflect better performance		
					Letter Fluency	Number of correct words generated; higher scores reflect better performance		
					Rule Shift Card Test	Number of errors; lower scores reflect better performance		
Physical activity	Karssemeijer 2019	67	12	12 weeks of exergaming vs aerobic (alternative treatment)	Physical Activity Scale for the Elderly (PASE)	PASE score ranges from 0 to 400 or more; higher scores reflect higher levels of physical activity	Post‐treatment mean (SD) of exergame vs control: 1. PASE = 78.6 (60.1) vs 89.4 (78.5), P > 0.05	Little to no difference between groups
Frailty	Karssemeijer 2019	67	12	12 weeks of exergaming vs aerobic (alternative treatment)	Evaluative Frailty Index for Physical Activity (EFIP)	Scores range from 0.00 to 1.00; lower scores reflect better performance	Post‐treatment mean (SD) of exergame vs control: 1. EFIP = 0.23 (0.12) vs 0.24 (0.13), P > 0.05	Little to no difference between groups
Quality of life	Swinnen 2023	18	12	Quality of life	Dementia Quality of Life (DQoL)	Scores range from 1 to 5; higher scores reflect better QoL	Post‐treatment mean (SD) of exergame vs control: 1. DQoL = 3.4 (1.0) vs 2.8 (0.4), P = 0.116	Little to no difference between groups

N: number; P: probability; SD: standard deviation; vs: versus

We found little to no difference in lower limb functioning between exergaming and alternative treatment groups of people with dementia at the end of treatment (SMD 0.21, 95% ‐0.74 to 1.16; 2 studies, 85 participants; Analysis 2.4).

We found little to no difference in functional status and physical performance between groups (MD 0.80, 95% ‐0.57 to 2.18; 2 studies, 85 participants; Analysis 2.2).

For balance, there was little to no difference between groups.

For general cognition, the intervention had a beneficial effect, but for attention, processing speed, and working memory, findings were mixed. For memory and executive functioning, there was little to no difference between groups.

For physical activity, frailty, and quality of life, there was little to no difference between groups.

#### Effects of interventions at the end of treatment for people with MCI

##### Exergaming versus control

Two studies assessed physical and cognitive functioning (Hughes 2014; Manser 2023), and two studies included ADL outcomes (Hughes 2014; Lee 2021) at the end of treatment for people with MCI.

Relative to a control (i.e. inactive control group, such as standard treatment, or an active control group, such as a healthy ageing education programme), we identified a medium effect favouring exergaming for global cognitive functioning, and little to no difference between groups in global physical functioning and ADL.

###### Primary outcomes

####### Global physical functioning

We found little to no difference in global physical functioning (SMD 0.27, 95% ‐0.41 to 0.94; 2 studies, 34 participants, very low certainty of evidence). Our certainty was very low because of serious concerns about increased risk of bias and imprecision. See Analysis 3.1 and Table 3.

####### Global cognitive functioning

We found a medium positive effect from exergaming for global cognitive functioning (SMD 0.79, 95% 0.05 to 1.53, 2 studies, 85 participants, very‐low certainty of evidence). Our certainty was very low because of serious concerns about increased risk of bias, inconsistency, and imprecision. See Analysis 3.2 and Table 3.

####### Activities of daily living

We found little to no difference between groups in ADL (SMD 0.51, 95% ‐0.01 to 1.03, 2 studies, 60 participants, very low certainty of evidence). Our certainty was very low because of serious concerns about increased risk of bias and imprecision. See Analysis 3.3 and Table 3.

###### Secondary outcomes

We pooled results for lower limb functioning, for general cognition, and for attention, processing speed, and working memory. For the remaining secondary outcomes, narrative results are presented in Table 10.

**6:** Secondary outcomes. Exergaming vs control at the end of treatment for people with MCI

**Secondary outcome domain**	**Study**	**N**	**Follow‐up (weeks)**	**Comparison**	**Measure(s)**	**Scale metrics**	**Numerical data**	**Narrative interpretation**
Memory	Manser 2023	14	12	12 weeks of exergaming versus usual care (control)	Subtest “logical memory” of Wechsler Memory Scale‐IV (LM‐WMS‐IV) part 1 free recall	Total points score; higher scores reflect better performance	Post‐treatment mean (SD) of exergame vs control: 1. LM‐WMS‐IV part 1 free recall = 25.75 (9.81) vs 26.33 (6.5) 2. LM‐WMS‐IV part 2 free recall = 5.38 (4.41) vs 9.33 (9.67); 3. LM‐WMS‐IV part 2 recognition = 15.9 (3.4) vs 17.6 (2.0) None of the comparisons were statistically significant.	Little to no difference between groups
					Subtest “logical memory” of WMS‐IV (LM‐WMS‐IV) part 2 free recall	Total points score; higher scores reflect better performance		
					Subtest “logical memory” of WMS‐IV (LM‐WMS‐IV) part 2 recognition	Total points score; higher scores reflect better performance		
Executive functioning	Manser 2023	14	12	12 weeks of exergaming versus usual care (control)	HOTAP picture‐sorting test part A combi score (HOTAP‐A)	Sum of the points divided by the time to arrange the cards; higher scores reflect better performance	Post‐treatment mean (SD) of exergame vs control: 1. HOTAP‐A = 4.7 (2.5) vs 5.8 (1.7); 2. TMT‐B‐RT = 129.25 (83.67) vs 69.26 (24.87); 3. TMT‐B‐errors = 5.29 (6.68) vs 5.83 (10.96) None of the comparisons were statistically significant.	Mixed findings
					Trail Making Test part B completion time (TMT‐B‐RT)	Time taken to complete the task; lower scores reflect better performance		
					Trail Making Test part B number of errors (TMT‐B‐errors)	Number of errors; lower scores reflect better performance		
Construction and motor performance	Manser 2023	14	12	12 weeks of exergaming versus usual care (control)	PEBL Mental Rotation Task reaction time (MRT‐RT)	RT for correct answered trials; lower scores reflect better performance	Post‐treatment mean (SD) of exergame vs control: 1. MRT‐RT = 3070.57 (592.81) vs 4049.83 (2517.85); 2. MRT‐TS = 49.1 (9.1) vs 44.7 (11.1) None of the comparisons were statistically significant.	Little to no difference between groups
					PEBL Mental Rotation Task total score (MRT‐TS)	The number of correct answered trials; higher scores reflect better performance		
Quality of life	Manser 2023	14	12	12 weeks of exergaming versus usual care (control)	Quality of life‐Alzheimer’s Disease (QoL‐AD)	Scores range from 13 to 52; higher scores reflect better QoL	Post‐treatment mean (SD) of exergame vs control: 1. QoL‐AD = 37.75 (6.78) vs 36.83 (3.25), P > 0.05	Little to no difference between groups

N: number; P: probability; SD: standard deviation; vs: versus

We found little to no difference in lower limb functioning between exergaming and control groups of people with MCI at the end of treatment (SMD 0.27, 95% ‐0.41 to 0.94; 2 studies, 34 participants; Analysis 3.4).

We found little to no difference between groups in general cognition (SMD 0.46, 95% ‐0.22 to 1.15; 2 studies, 34 participants; Analysis 3.5).

We found little to no difference between groups in attention, processing speed, and working memory (SMD 1.14, 95% ‐0.03 to 2.31; 2 studies, 34 participants; Analysis 3.6).

For memory, for construction and motor performance, and for quality of life, we found little to no difference between groups. There were mixed findings for executive functioning.

##### Exergaming versus alternative treatment

###### Primary outcomes

Only one study included physical functioning outcomes (Ramnath 2021), and four studies included cognitive functioning outcomes at the end of treatment for people with MCI (Arshad 2023; Park 2018; Ramnath 2021; Torpil 2020). No study evaluated ADL outcomes.

Relative to alternative treatment (e.g. treatment with specific effect such as stretching and strength training, standing and seated exercises, computerised cognitive training), we found little to no difference in global physical functioning. For global cognitive functioning, the evidence is very uncertain.

####### Global physical functioning

Ramnath 2021 reported little to no difference between groups for global physical functioning (n = 45; low certainty of evidence). Our certainty was low because of serious concerns about increased risk of bias and imprecision. Table 11 provides numerical data, outcome definitions used within the study, a brief narrative interpretation, and the overall risk of bias. See Table 4.

**7:** Analysis 4.1 Exergaming vs alternative treatment at the end of treatment for people with MCI: change in global physical functioning (composite)

**Study**	**N**	**Follow‐up (weeks)**	**Comparison**	**Physical domain: measure(s)**	**Scale metrics**	**Numerical data**	**Narrative interpretation**	**Risk of bias**
Ramnath 2021	45	12	12 weeks of exergaming vs conventional multimodal training (alternative treatment)	Lower limb function: Timed Up & Go Test s (TUG)	Time taken to complete the task in seconds; lower scores reflect better performance	Mean (SD) of exergame vs alternative treatment and P value of difference between groups: 1. TUG = 6.2 (1.24) vs 7.4 (1.27), P > 0.05; 2. 6‐MWT = 513.7 (83.45) vs 507.7 (83.49), P > 0.05; 3. 10m Test = 35.7 (11.03) vs 43.8 (11.26), P > 0.05; 4. FRT = 85.9 (4.56) vs 82.0 (4.55), P > 0.05 No improvements between baseline and at the end of treatment on TUG, 10m Test, FRT.	Little to no difference between groups	Overall risk of bias: high. Comment: there are some or high concerns in most domains.
				Lower limb function: 6‐Minute Walk Test (6‐MWT)	Total distance covered in metres; higher scores reflect better performance			
				Balance: Dynamic Balance 10‐meter Test	Time taken to complete the first 6 steps and to complete the 10 metres in seconds; lower scores reflect better performance			
				Balance: Functional Reach Test (FRT)	Ability to reach in centimetres; higher scores reflect better performance			

N: number; P: probability; SD: standard deviation; vs: versus

####### Global cognitive functioning

For this comparison, we included four studies (n = 235), but due to considerable heterogeneity (I² = 96%), we could not pool results. Our narrative interpretation suggests that the evidence is very uncertain. Arshad 2023 (n = 31) and Torpil 2020 (n = 61) identified effects in favour of exergaming on global cognitive functioning. Park 2018 (n = 48) reported mixed findings, and Ramnath 2021 (n =45) reported little to no difference. Our certainty was very low because of serious concerns about high risk of bias, inconsistency, and imprecision. Table 12 provides numerical data, outcome definitions used within the study, a brief narrative interpretation, and the overall risk of bias. See Table 4.

**8:** Analysis 4.2 Exergaming vs alternative treatment at the end of treatment for people with MCI: change in global cognitive functioning (composite)

**Study**	**N**	**Follow‐up (weeks)**	**Comparison**	**Cognitive domain: measure(s)**	**Scale metrics**	**Numerical data**	**Narrative interpretation**	**Risk of bias**
Arshad 2023	31	6	6 weeks of exergaming vs stretching and strength training (alternative treatment)	General cognition: Mini‐Mental State Exam (MMSE)	Range 0 to 30; higher scores reflect better performance	Post‐treatment mean (SD) of exergame vs alternative treatment and P value of difference between groups: 1. MMSE = 24.23 (1.63) vs 23.00 (2.00), P = 0.02; 2. MoCA = 22.35 (1.57) vs 21.16 (1.79), P = 0.015; 3. TMT‐A = 1.94 (0.19) vs 2.15 (0.27), P = 0.003; 4. TMT‐B = 3.67 (0.56) vs 4.50 (0.68), P = 0.005; 5. VFT‐Semantic = 10.46 (1.55) vs 9.32 (1.46), P = 0.010; 6. VFT‐Phonemic = 7.27 (1.21) vs 5.52 (1.58), P = 0.008 Within‐group comparisons between baseline mean (SD) vs post‐treatment mean (SD) in intervention group: 1. MMSE = 21.23 (1.88) vs 24.23 (1.63), P = 0.003; 2. MoCA = 19.58 (2.31) vs 22.35 (1.57), P = 0.005; 3. TMT‐A = 2.38 (0.35) vs 1.94 (0.19), P = 0.001; 4. TMT‐B = 4.93 (0.50) vs 3.67 (0.56), P = 0.002; 5. VFT‐Semantic = 8.27 (1.34) vs 10.46 (1.55), P = 0.015; 6. VFT‐Phonemic = 4.96 (1.48) vs 7.27 (1.21), P = 0.002 Within‐group comparisons between baseline mean (SD) vs post‐treatment mean (SD) in alternative treatment group: 1. MMSE = 21.12 (2.12) vs 23.00 (2.00), P = 0.005; 2. MoCA = 19.40 (2.21) vs 21.16 (1.79), P = 0.007; 3. TMT‐A = 2.19 (0.20) vs 2.15 (0.27), P = 0.177; 4. TMT‐B = 4.62 (0.51) vs 4.50 (0.68), p = 0.069; 5. VFT‐Semantic = 8.24 (1.01) vs 9.32 (1.46), P = 0.183; 6. VFT‐Phonemic = 5.04 (1.69) vs 5.52 (1.58), P = 0.076	Intervention had a beneficial effect on all 5 scales.	Overall risk of bias: high. There are some concerns or high risk of bias in most domains.
				General cognition: Montreal Cognitive Assessment (MoCA)	Range 0 to 30; higher scores reflect better performance			
				Attention, processing speed, and working memory: Trail Making Test part A (TMT‐A)	Time taken to complete the task; lower scores reflect better performance			
				Executive functioning: Trail Making Test part B (TMT‐B)	Time taken to complete the task; lower scores reflect better performance			
				Executive functioning: Verbal Fluency test (VFT) Semantic	Number of words generated; higher scores reflect better performance			
				Executive functioning: Verbal Fluency test (VFT) Phonemic	Number of words generated; higher scores reflect better performance			
Park 2018	78	10	10 weeks of exergaming vs computerised cognitive training (alternative treatment)	Attention, processing speed, and working memory: Digit Span Forward	Number of digits correctly repeated; higher scores reflect better performance	Post‐treatment mean (SD) of exergame vs alternative treatment and P value of difference between groups: 1. Digit Span Forward = 7.61 (0.19) vs 7.29 (0.18), P < 0.05; 2. Digit Span Backward = 3.78 (0.23) vs 3.39 (0.23), P < 0.05; 3. RAVLT = 106.46 (8.2) vs 107.03 (8.34), P > 0.05; 4. RCFT = 101.00 (5.63) vs 101.92 (4.74), P > 0.05; 5. WAIS‐BDT = 27.03 (2.6) vs 26.92 (2.76), P > 0.05; 6. TMT‐B = 129.38 (7.02) vs 128.21 (8.71), P > 0.05; 7. Stroop = 26.77 (3.15) vs 26.97 (2.49), P > 0.05 Within‐group change from baseline to end of treatment mean (SD) in intervention group: 1. Digit Span Forward = 0.40 (0.08), P < 0.05; 2. Digit Span Backward = 0.46 (0.09), P < 0.05; 3. RAVLT = 9.31 (2.54), P < 0.05; 4. RCFT = 5.92 (1.86), P < 0.05; 5. WAIS‐BDT = 2.15 (0.99), P < 0.05; 6. TMT‐B = 1.31 (0.98), P > 0.05; 7. Stroop = 1.15 (0.84), P > 0.05 Within‐group change from baseline to end of treatment mean (SD) in alternative treatment group: 1. Digit Span Forward = 0.12 (0.04), P < 0.05; 2. Digit Span Backward = 0.11 (0.04), P < 0.05; 3. RAVLT = 9.26 (4.38), P < 0.05; 4. RCFT = 5.84 (3.34), P < 0.05; 5. WAIS‐BDT = 1.97 (0.99), P < 0.05; 6. TMT‐B = 1.38 (1.21), P > 0.05; 7. Stroop = 0.90 (0.72), P > 0.05	Mixed findings	Overall risk of bias: some concerns. The study is judged to have low risk of bias in most domains, but there are some concerns for bias due to randomisation process and bias in selection of the reported result.
				Attention, processing speed, and working memory: Digit Span Backward	Number of digits correctly repeated; higher scores reflect better performance			
				Memory: Memory‐Rey Auditory Verbal Learning Test (RAVLT)	Number of words correctly recalled; higher scores reflect better performance			
				Memory: Rey–Osterrieth Complex Figure Test (RCFT)	Number of words correctly recalled; higher scores reflect better performance			
				Construction and motor performance: Visouspatial ability‐Wechsler Adult Intelligence Scale‐Block Design Test (WAIS‐BDT)	Scores can range from 0 to 48; higher scores reflect better performance			
				Executive functioning: Trail Making Test part B (TMT‐B)	Time taken to complete the task; lower scores reflect better performance			
				Executive functioning: Stroop Color‐Word Test	Time and accuracy; lower scores reflect better performance			
Ramnath 2021	45	12	12 weeks of exergaming vs conventional multimodaltraining (alternative treatment)	General cognition: Mini‐Mental State Exam (MMSE)	Range 0 to 30; higher scores reflect better performance	Mean (SD) of exergame vs alternative treatment and P value of difference between groups: 1. MMSE = 27.3 (2.49) vs 25.3 (2.49), P > 0.05; 2. Stroop Total Correct Responses = 80.7 (20.96) vs 74.8 (20.97), P > 0.05; 3. Stroop Average RT of all Correct Responses = 1594.7 (237.97) vs 1660.3 (238.23), P > 0.05; 4. Stroop Correct Grey (Neutral) Words = 74.3 (25.42) vs 79.7 (25.47), P > 0.05; 5. Stroop Average RT of 6. Correct Grey (Neutral) Words = 1770.3 (419.01) vs 1725.6 (419.46), P > 0.05; 7. Stroop Correct Colour (Incongruent) Words = 82.9 (21.92) vs 73.2 (21.95), P > 0.05; 8. Stroop Average RT of Correct Colour (Incongruent) Words = 1533.3 (261.28) vs 1613.8 (261.58), P > 0.05; 9. Stroop Average RT of total number of mistakes = 1434.9 (685.56) vs 1503.6 (686.35), P > 0.05; 10. Stroop Average RT of Incorrect Colour (Incongruent) Words = 1155.7 (731.41) vs 1487.9 (732.27), P > 0.05; 11. Stroop Average RT of Incorrect Grey (Neutral) Words = 994.3 (896.53) vs 705.8 (897.56), P > 0.05; 12. N‐back 0‐Back Score = 98.6 (1.77) vs 97.5 (1.74), P > 0.05; 13. N‐back 1‐Back Score = 96.0 (5.66) vs 94.6 (5.68), P > 0.05; 14. N‐back 2‐Back Score = 73.8 (18.13) vs 73.7 (18.15), P > 0.05 No improvements between baseline and at the end of treatment on any outcomes.	Little to no difference between groups	Overall risk of bias: high. There are some concerns or high risk of bias in most domains.
				Attention, processing speed, and working memory: Stroop Total Correct Responses	Number of correct responses; higher scores reflect better performance			
				Attention, processing speed, and working memory: Stroop Average reaction time of all Correct Responses	Reaction time; lower scores reflect better performance			
				Attention, processing speed, and working memory: Stroop Correct Grey (Neutral) Words	Number of correct responses; higher scores reflect better performance			
				Attention, processing speed, and working memory: Stroop Average reaction time of Correct Grey (Neutral) Words	Reaction time; lower scores reflect better performance			
				Attention, processing speed, and working memory: Stroop Correct Colour (Incongruent) Words	Number of correct responses; higher scores reflect better performance			
				Attention, processing speed, and working memory: Stroop Average reaction time of Correct Colour (Incongruent) Words	Reaction time; lower scores reflect better performance			
				Attention, processing speed, and working memory: Stroop Average reaction time of total number of mistakes	Reaction time; lower scores reflect better performance			
				Attention, processing speed, and working memory: Stroop Average reaction time of Incorrect Colour (Incongruent) Words	Reaction time; lower scores reflect better performance			
				Attention, processing speed, and working memory: Stroop Average reaction time of Incorrect Grey (Neutral) Words	Reaction time; lower scores reflect better performance			
				Attention, processing speed, and working memory: N‐back 0‐Back Score	Total number correct targets achieved; higher scores reflect better performance			
				Attention, processing speed, and working memory: N‐back 1‐Back Score	Total number correct targets achieved; higher scores reflect better performance			
				Attention, processing speed, and working memory: N‐back 2‐Back Score	Total number correct targets achieved; higher scores reflect better performance			
Torpil 2020	61	12	12 weeks of exergaming vs computer cognitive rehabilitation (alternative treatment)	General cognition: Loewenstein Occupational Therapy Cognitive Assessment‐Geriatric (LOTCA‐G)	Range 26 to 115; higher scores reflect better performance	Mean (SD) of exergame vs alternative treatment and P value of difference between groups: 1. LOTCA‐G = 97.50 (2.44) vs 93.06 (3.38), P < 0.001 Within‐group comparisons between baseline mean (SD) vs post‐treatment mean (SD) in intervention group: 1. LOTCA‐G = 84.38 (3.91) vs 97.50 (2.44), P < 0.001 Within‐group comparisons between baseline mean (SD) vs post‐treatment mean (SD) in alternative treatment group: 1. LOTCA‐G = 84.66 (3.32) vs 93.06 (3.38), P < 0.05	Intervention had a beneficial effect on 1/1 scale.	Overall risk of bias: some concerns. There are some concerns of bias in most domains.

N: number; P: probability; SD: standard deviation; vs: versus

####### Activities of daily living

No study evaluated ADL outcomes.

###### Secondary outcomes

Narrative results are presented in Table 13.

**9:** Secondary outcomes. Exergaming vs alternative treatment at the end of treatment for people with MCI

**Secondary outcome domain**	**Study**	**N**	**Follow‐up (weeks)**	**Comparison**	**Measure(s)**	**Scale metrics**	**Numerical data**	**Narrative interpretation**
Lower limb function	Ramnath 2021	45	12	12 weeks of exergaming vs conventional multimodaltraining (alternative treatment)	Timed Up & Go Test s (TUG)	Time taken to complete the task in seconds; lower scores reflect better performance	Mean (SD) of exergame vs alternative treatment and P value of difference between groups: 1. TUG = 6.2 (1.24) vs 7.4 (1.27), P > 0.05; 2. 6MWT = 513.7 (83.45) vs 507.7 (83.49), P > 0.05 No improvements between baseline and at the end of treatment on TUG.	Little to no difference between groups
					6‐Minute Walk Test (6MWT)	Total distance covered in metres; higher scores reflect better performance		
Balance	Ramnath 2021	45	12	12 weeks of exergaming vs conventional multimodaltraining (alternative treatment)	Dynamic Balance 10‐meter Test	Time taken to complete the first 6 steps and to complete the 10 metres in seconds; lower scores reflect better performance	Mean (SD) of exergame vs alternative treatment and P value of difference between groups: 1. 10m Test = 35.7 (11.03) vs 43.8 (11.26), P > 0.05; 2. FRT = 85.9 (4.56) vs 82.0 (4.55), P > 0.05 No improvements between baseline and at the end of treatment on 10m Test, FRT.	Little to no difference between groups
					Functional Reach Test (FRT)	Ability to reach in centimetres; higher scores reflect better performance		
General cognition	Arshad 2023	31	6	6 weeks of exergaming vs stretching and strength training (alternative treatment)	Mini‐Mental State Exam (MMSE)	Range 0 to 30; higher scores reflect better performance	Post‐treatment mean (SD) of exergame vs alternative treatment and P value of difference between groups: 1. MMSE = 24.23 (1.63) vs 23.00 (2.00), P = 0.02; 2. MoCA = 22.35 (1.57) vs 21.16 (1.79), P = 0.015; 3. MMSE = 27.3 (2.49) vs 25.3 (2.49), P > 0.05 Mean (SD) of exergame vs alternative treatment and P value of difference between groups: 1. LOTCA‐G = 97.50 (2.44) vs 93.06 (3.38), P < 0.001 Within‐group comparisons between baseline mean (SD) vs post‐treatment mean (SD) in intervention group: 2. LOTCA‐G = 84.38 (3.91) vs 97.50 (2.44), P < 0.001 Within‐group comparisons between baseline mean (SD) vs post‐treatment mean (SD) in alternative treatment group: 1. LOTCA‐G = 84.66 (3.32) vs 93.06 (3.38), P < 0.05	Intervention had a beneficial effect on some scales.
					Montreal Cognitive Assessment (MoCA)	Range 0 to 30; higher scores reflect better performance		
	Ramnath 2021	31	6	6 weeks of exergaming vs stretching and strength training (alternative treatment)	Mini‐Mental State Exam (MMSE)	Range 0 to 30; higher scores reflect better performance		
	Torpil 2020	61	12	12 weeks of exergaming vs computer cognitive rehabilitation (alternative treatment)	Loewenstein Occupational Therapy Cognitive Assessment‐Geriatric (LOTCA‐G)	Range 26 to 115; higher scores reflect better performance		
Attention, processing speed, and working memory	Arshad 2023	31	6	6 weeks of exergaming vs stretching and strength training (alternative treatment)	Trail Making Test part A (TMT‐A)	Time taken to complete the task; lower scores reflect better performance	Post‐treatment mean (SD) of exergame vs alternative treatment and P value of difference between groups: 1. TMT‐A = 1.94 (0.19) vs 2.15 (0.27), P = 0.003 Post‐treatment mean (SD) of exergame vs alternative treatment and P value of difference between groups: 2. Digit Span Forward = 7.61 (0.19) vs 7.29 (0.18), P < 0.05; 3. Digit Span Backward = 3.78 (0.23) vs 3.39 (0.23), P < 0.05 Mean (SD) of exergame vs alternative treatment and P value of difference between groups: 4. Stroop Total Correct Responses = 80.7 (20.96) vs 74.8 (20.97), P > 0.05; 5. Stroop Average RT of all Correct Responses = 1594.7 (237.97) vs 1660.3 (238.23), P > 0.05; 6. Stroop Correct Grey (Neutral) Words = 74.3 (25.42) vs 79.7 (25.47), P > 0.05; 7. Stroop Average RT of 8. Correct Grey (Neutral) Words = 1770.3 (419.01) vs 1725.6 (419.46), P > 0.05; 9. Stroop Correct Colour (Incongruent) Words = 82.9 (21.92) vs 73.2 (21.95), P > 0.05; 10. Stroop Average RT of Correct Colour (Incongruent) Words = 1533.3 (261.28) vs 1613.8 (261.58), P > 0.05; 11. Stroop Average RT of total number of mistakes = 1434.9 (685.56) vs 1503.6 (686.35), P > 0.05 12. Stroop Average RT of Incorrect Colour (Incongruent) Words = 1155.7 (731.41) vs 1487.9 (732.27), P > 0.05; 13. Stroop Average RT of Incorrect Grey (Neutral) Words = 994.3 (896.53) vs 705.8 (897.56), P > 0.05; 14. N‐back 0‐Back Score = 98.6 (1.77) vs 97.5 (1.74), P > 0.05; 15. N‐back 1‐Back Score = 96.0 (5.66) vs 94.6 (5.68), P > 0.05; 16. N‐back 2‐Back Score = 73.8 (18.13) vs 73.7 (18.15), P > 0.05	Mixed findings
	Park 2018	78	10	10 weeks of exergaming vs computerised cognitive training (alternative treatment)	Digit Span Forward	Number of digits correctly repeated; higher scores reflect better performance		
					Digit Span Backward	Number of digits correctly repeated; higher scores reflect better performance		
	Ramnath 2021	31	6	6 weeks of exergaming vs stretching and strength training (alternative treatment)	Stroop Total Correct Responses	Number of correct responses; higher scores reflect better performance		
					Stroop Average reaction time of all Correct Responses	Reaction time; lower scores reflect better performance		
					Stroop Correct Grey (Neutral) Words	Number of correct responses; higher scores reflect better performance		
					Stroop Average reaction time of Correct Grey (Neutral) Words	Reaction time; lower scores reflect better performance		
					Stroop Correct Colour (Incongruent) Words	Number of correct responses; higher scores reflect better performance		
					Stroop Average reaction time of Correct Colour (Incongruent) Words	Reaction time; lower scores reflect better performance		
					Stroop Average reaction time of total number of mistakes	Reaction time; lower scores reflect better performance		
					Stroop Average reaction time of Incorrect Colour (Incongruent) Words	Reaction time; lower scores reflect better performance		
					Stroop Average reaction time of Incorrect Grey (Neutral) Words	Reaction time; lower scores reflect better performance		
					N‐back 0‐Back Score	Total number correct targets achieved; higher scores reflect better performance		
					N‐back 1‐Back Score	Total number correct targets achieved; higher scores reflect better performance		
					N‐back 2‐Back Score	Total number correct targets achieved; higher scores reflect better performance		
Memory	Park 2018	78	10	10 weeks of exergaming vs computerised cognitive training (alternative treatment)	Memory‐Rey Auditory Verbal Learning Test (RAVLT)	Number of words correctly recalled; higher scores reflect better performance	Post‐treatment mean (SD) of exergame vs alternative treatment and P value of difference between groups: 1. RAVLT = 106.46 (8.2) vs 107.03 (8.34), P > 0.05; 2. RCFT = 101.00 (5.63) vs 101.92 (4.74), P > 0.05	Little to no difference between groups
					Rey–Osterrieth Complex Figure Test (RCFT)	Number of words correctly recalled; higher scores reflect better performance		
Executive functioning	Arshad 2023	31	6	6 weeks of exergaming vs stretching and strength training (alternative treatment)	Trail Making Test part B (TMT‐B)	Time taken to complete the task; lower scores reflect better performance	Post‐treatment mean (SD) of exergame vs alternative treatment and P value of difference between groups: 1. TMT‐B = 3.67 (0.56) vs 4.50 (0.68), P = 0.005; 2. VFT‐Semantic = 10.46 (1.55) vs 9.32 (1.46), P = 0.010; 3. VFT‐Phonemic = 7.27 (1.21) vs 5.52 (1.58), P = 0.008 4. TMT‐B = 129.38 (7.02) vs 128.21 (8.71), P > 0.05; 5. Stroop = 26.77 (3.15) vs 26.97 (2.49), P > 0.05	Mixed findings
					Verbal Fluency test (VFT) Semantic	Number of words generated; higher scores reflect better performance		
					Verbal Fluency test (VFT) Phonemic	Number of words generated; higher scores reflect better performance		
	Park 2018	78	10	10 weeks of exergaming vs computerised cognitive training (alternative treatment)	Trail Making Test part B (TMT‐B)	Time taken to complete the task; lower scores reflect better performance		
					Stroop Color‐Word Test	Time and accuracy; Lower scores reflect better performance		
Construction and motor performance	Park 2018	78	10	10 weeks of exergaming vs computerised cognitive training (alternative treatment)	Visouspatial ability‐Wechsler Adult Intelligence Scale‐Block Design Test (WAIS‐BDT)	Scores can range from 0 to 48; higher scores reflect better performance	Post‐treatment mean (SD) of exergame vs alternative treatment and P value of difference between groups: 1. WAIS‐BDT = 27.03 (2.6) vs 26.92 (2.76), P > 0.05	Little to no difference between groups
Quality of life	Park 2018	78	10	10 weeks of exergaming vs computerised cognitive training (alternative treatment)	Short‐Form Health Survey (SF‐36)	Consists of 36 items and 8 subscales: (1) physical functioning, (2) role‐physical, (3) bodily pain, (4) general health, (5) vitality, (6) social functioning, (7) role‐emotional, and (8) mental health; scores range from 0 to 100 (for each individual subscale); higher scores reflect better health ‐related QoL	Post‐treatment mean (SD) of exergame vs alternative treatment and P value of difference between groups: 1. SF36 – QoL Vitality = 72.62 (4.78) vs 62.63 (3.66) P < 0.05 2. SF36 – QoL Social Functioning = 79.18 (5.25) vs 75.69 (3.59) P > 0.05 3. SF36 – Role emotional = 75.79 (5.15) vs 69.79 (3.83) P < 0.05 4. SF36 – Mental health = 88.56 (5.97) vs 82.69 (3.73) P < 0.05 5. SF36 – Physical functioning = 75.49 (4.15) vs 71.74 (3.48) P > 0.05 6. SF36 – Role physical = 72.41 (4.16) vs 68.67 (3.37) P > 0.05 7. SF36 – Bodily pain = 72.67 (5.05) vs 64.74 (3.65) P < 0.05 8. SF36 – General health = 66.83 (5.01) vs 62.26 (3.92) P > 0.05	Mixed findings

N: number; P: probability; QoL: quality of life; SD: standard deviation; vs: versus

For lower limb function and for balance, we found little to no difference between exergaming and alternative treatment groups of people with MCI at the end of treatment.

For general cognition, exergaming had a beneficial effect compared to alternative treatments. For attention, processing speed, and working memory, and for executive functioning, findings were mixed. For memory and for construction and motor performance, we found little to no difference between groups.

For quality of life, we found mixed findings.

#### Effects of interventions at follow‐up for people with dementia

##### Exergaming versus control

###### Primary outcomes

Only one study included physical and cognitive functioning outcomes (Karssemeijer 2019). Robert 2021 included only cognitive functioning outcomes at follow‐up for people with dementia. No study assessed ADL outcomes at follow‐up.

Relative to a control group (i.e. inactive control group such as standard treatment and active control group such as relaxation and flexibility exercises), overall there was little to no difference in global physical and cognitive functioning.

####### Global physical functioning

Karssemeijer 2019 reported little to no difference between groups in global cognitive functioning (n = 62). Table 14 provides numerical data, outcome definitions used within the study, and a brief narrative interpretation.

**10:** Analysis 5.1 Exergaming vs control at follow‐up for people with dementia: change in global physical functioning

**Study**	**N**	**Follow‐up (weeks)**	**Comparison**	**Physical domain: measure(s)**	**Scale metrics**	**Numerical data**	**Narrative interpretation**
Karssemeijer 2019	62	24	12 weeks of exergaming vs relaxation and flexibility exercises (control); follow‐up assessed at 24 weeks	Lower limb function: 10 m Walk Test (m/s)	Walking speed in m/s; higher scores reflect better performance	Follow‐up mean (SD) of exergame vs control: 1. 10 m Walk Test = 1.02 (0.33) vs 1.00 (0.30); 2. TUG = 13.8 (5.5) vs 14.7 (7.3); 3. 5TSST = 15.9 (5.7) vs 17.9 (10.1); 4. FICSIT‐4 = 3.8 (1.1) vs 3.7 (1.4) No between‐group differences (all Ps > 0.05) No improvements between baseline and follow‐up	Little to no difference between groups
				Lower limb function: Timed Up & Go Test s (TUG)	Time to complete the test; lower scores reflect better performance		
				Lower limb function: Five Times Sit‐to‐stand test (5TSST) (sec)	Time to complete the test; lower scores reflect better performance		
				Balance: Frailty and Injuries Cooperative Studies of Interventions Technique Subtest 4 (FICSIT‐4 score)	Score ranges from 0 to 5; higher score reflect better performance		

m: metres; N: number; s: seconds; SD: standard deviation; vs: versus

####### Global cognitive functioning

We aimed to pool results for global cognitive functioning but, due to considerable heterogeneity (I² = 94%), this was not appropriate. Karssemeijer 2019 reported mixed findings (n = 62), and Robert 2021 found little to no difference between groups (n = 50). Table 15 provides numerical data, outcome definitions used within the study, and a brief narrative interpretation.

**11:** Analysis 5.2 Exergaming vs control at follow‐up for people with dementia: change in global cognitive functioning

**Study**	**N**	**Follow‐up (weeks)**	**Comparison**	**Cognitive domain: measure(s)**	**Scale metrics**	**Numerical data**	**Narrative interpretation**
Karssemeijer 2019	62	24	12 weeks of exergaming vs relaxation and flexibility exercises (control); follow‐up assessed at 24 weeks	Attention, processing speed, and working memory: Trail Making Test part A (TMT‐A)	Time taken to complete the task; lower scores reflect better performance	Follow‐up mean (SD) of exergame vs control: 1. TMT‐A = 55.8 (54.0) vs 68.5 (73.4) 2. Stroop Test word‐reading = 41.1 (16.4) vs 49.7 (33.4); 3. Stroop Test color‐naming = 55.0 (26.1) vs 61.4 (36.1); 4. WAIS‐III Digit Span = 9.6 (3.3) vs 9.8 (3.6); 5. WMS‐III Spatial Span = 7.7 (3.8) vs 9.3 (4.2); 6. LLT 1‐5 = 96.1 (34.1) vs 118.8 (51.2); 7. LLT delayed recall = 18.5 (8.1) vs 23.3 (10.8); 8. TMT‐B = 178.1 (105.4) vs 166.1 (105.1); 9. Stroop Test speed‐accuracy trade off scores = 0.34 (0.18) vs 0.29 (0.21); 10. Stroop Test colour‐word interference card = 139.2 (75.2) vs 136.5 (74.5); 11. Stroop Test colour‐word Interference card (number of errors) = 6.1 (8.8) vs 8.1 (12.5); 12. Letter Fluency = 20.9 (11.2) vs 21.7 (15.1); 13. Rule Shift Card Test = 9.2 (5.5) vs 8.9 (6.7) Improvement in psychomotor speed for the exergame vs control (mean difference domain score (95% CI) 0.326 (0.070 to 0.604), P = 0.014. The remaining comparisons were not statistically significant.	Mixed findings
				Attention, processing speed, and working memory: Stroop Test word‐reading	Time taken to complete the task; lower scores reflect better performance		
				Attention, processing speed, and working memory: Stroop Test color‐naming	Time taken to complete the task; lower scores reflect better performance		
				Attention, processing speed, and working memory: Wechsler Adult Intelligence Scale‐III Digit Span	Number of digits correctly repeated; higher scores reflect better performance		
				Attention, processing speed, and working memory: Wechsler Memory Scale‐III Spatial Span	Number of digits correctly repeated; higher scores reflect better performance		
				Memory: Location Learning Test Displacement score trial 1 to 5 (LLT 1 to 5)	Sum of errors; lower scores reflect better performance		
				Memory: Location Learning Test Displacement score delayed recall (LLT delayed recall)	Sum of errors; lower scores reflect better performance		
				Executive functioning: Trail Making Test part B (TMT‐B)	Time taken to complete the task; lower scores reflect better performance		
				Executive functioning: Stroop Test speed‐accuracy trade off scores	Speed‐accuracy trade‐off scores calculated with the formula: (100*accuracy) / reaction time; lower scores reflect better performance		
				Executive functioning: Stroop Test colour‐word interference card	Time taken to complete the task; lower scores reflect better performance		
				Executive functioning: Stroop Test colour‐word Interference card (number of errors)	Number of errors; lower scores reflect better performance		
				Executive functioning: Letter Fluency	Number of correct words generated; higher scores reflect better performance		
				Executive functioning: Rule Shift Card Test	Number of errors; lower scores reflect better performance		
Robert 2021	50	24	12 weeks of exergaming vs standard care (control); follow‐up assessed at 24 weeks	General cognition: Mini‐Mental State Exam (MMSE)	Range 0 to 30; higher scores reflect better performance	Follow‐up mean (SD) of exergame vs control: 1. MMSE = 22.9 (3.7) vs 21.5 (3.7) No improvements between baseline and follow‐up on MMSE	Little to no difference between groups

N: number; P: probability; SD: standard deviation; vs: versus

####### Activities of daily living

No study assessed ADL outcomes.

###### Secondary outcomes

Narrative results are presented in Table 16.

**12:** Secondary outcomes. Exergaming vs control at follow‐up for people with dementia

**Secondary outcome domain**	**Study**	**N**	**Follow‐up (weeks)**	**Comparison**	**Measure(s)**	**Scale metrics**	**Numerical data**	**Narrative interpretation**
Lower limb function	Karssemeijer 2019	62	24	12 weeks of exergaming vs relaxation and flexibility exercises (control); follow‐up assessed at 24 weeks	10 m Walk Test (metres/seconds)	Walking speed in m/seconds; higher scores reflect better performance	Follow‐up mean (SD) of exergame vs control: 1. 10 m Walk Test = 1.02 (0.33) vs 1.00 (0.30); 2. TUG = 13.8 (5.5) vs 14.7 (7.3); 3. 5TSST = 15.9 (5.7) vs 17.9 (10.1) No between‐group differences (all Ps > 0.05). No improvements between baseline and follow‐up.	Little to no difference between groups
					Timed Up & Go Test (TUG)	Time to complete the test; lower scores reflect better performance		
					Five Times Sit‐to‐stand test (5TSST) (seconds)	Time to complete the test; lower scores reflect better performance		
Balance	Karssemeijer 2019	62	24	12 weeks of exergaming vs relaxation and flexibility exercises (control); follow‐up assessed at 24 weeks	Frailty and Injuries Cooperative Studies of Interventions Technique Subtest 4 (FICSIT‐4 score)	Score ranges from 0 to 5; higher score reflect better performance	Follow‐up mean (SD) of exergame vs control: 1. FICSIT‐4 = 3.8 (1.1) vs 3.7 (1.4) No between‐group differences (P > 0.05). No improvements between baseline and follow‐up.	Little to no difference between groups
General cognition	Robert 2021	20	12	12 weeks of exergaming vs standard care (control); follow‐up assessed at 24 weeks	Mini‐Mental State Exam (MMSE)	Range 0 to 30; higher scores reflect better performance	Follow‐up mean (SD) of exergame vs control: 1. MMSE = 22.9 (3.7) vs 21.5 (3.7) No improvements between baseline and follow‐up on MMSE.	Little to no difference between groups
Attention, processing speed, and working memory	Karssemeijer 2019	62	24	12 weeks of exergaming vs relaxation and flexibility exercises (control); follow‐up assessed at 24 weeks	Trail Making Test part A (TMT‐A)	Time taken to complete the task; lower scores reflect better performance	Follow‐up mean (SD) of exergame vs control: 1. TMT‐A = 55.8 (54.0) vs 68.5 (73.4) 2. Stroop Test word‐reading = 41.1 (16.4) vs 49.7 (33.4); 3. Stroop Test color‐naming = 55.0 (26.1) vs 61.4 (36.1); 4. WAIS‐III Digit Span = 9.6 (3.3) vs 9.8 (3.6); 5. WMS‐III Spatial Span = 7.7 (3.8) vs 9.3 (4.2) Improvement in processing speed for exergame vs control (mean difference domain score (95% CI) 0.326 (0.070 to 0.604), P = 0.014. The remaining comparisons were not statistically significant.	Mixed findings
					Stroop Test word‐reading	Time taken to complete the task; lower scores reflect better performance		
					Stroop Test color‐naming	Time taken to complete the task; lower scores reflect better performance		
					Wechsler Adult Intelligence Scale‐III Digit Span (WAIS‐III Digit Span)	Number of digits correctly repeated; higher scores reflect better performance		
					Wechsler Memory Scale‐III Spatial Span (WMS Digit Span)	Number of digits correctly repeated; higher scores reflect better performance		
Memory	Karssemeijer 2019	62	24	12 weeks of exergaming vs relaxation and flexibility exercises (control); follow‐up assessed at 24 weeks	Location Learning Test Displacement score trial 1 to 5 (LLT 1 to 5)	Sum of errors; lower scores reflect better performance	Follow‐up mean (SD) of exergame vs control: 1. LLT 1‐5 = 96.1 (34.1) vs 118.8 (51.2); 2. LLT delayed recall = 18.5 (8.1) vs 23.3 (10.8) None of the comparisons were statistically significant.	Little to no difference between groups
					Location Learning Test Displacement score delayed recall (LLT delayed recall)	Sum of errors; lower scores reflect better performance		
Executive functioning	Karssemeijer 2019	62	24	12 weeks of exergaming vs relaxation and flexibility exercises (control); follow‐up assessed at 24 weeks	Trail Making Test part B (TMT‐B)	Time taken to complete the task; lower scores reflect better performance	Follow‐up mean (SD) of exergame vs control: 1. TMT‐B = 178.1 (105.4) vs 166.1 (105.1) 2. Stroop Test speed‐accuracy trade off scores = 0.34 (0.18) vs 0.29 (0.21); 3. Stroop Test colour‐word interference card = 139.2 (75.2) vs 136.5 (74.5); 4. Stroop Test colour‐word Interference card (number of errors) = 6.1 (8.8) vs 8.1 (12.5); 5. Letter Fluency = 20.9 (11.2) vs 21.7 (15.1); 6. Rule Shift Card Test = 9.2 (5.5) vs 8.9 (6.7) None of the comparisons were statistically significant.	Little to no difference between groups
					Stroop Test speed‐accuracy trade off scores	Speed‐accuracy trade‐off scores are calculated with the formula: (100*accuracy) / reaction time; lower scores reflect better performance		
					Stroop Test colour‐word interference card	Time taken to complete the task; lower scores reflect better performance		
					Stroop Test colour‐word Interference card (number of errors)	Number of errors; lower scores reflect better performance		
					Letter Fluency	Number of correct words generated; higher scores reflect better performance		
					Rule Shift Card Test	Number of errors; lower scores reflect better performance		
Functional status and physical performance	Karssemeijer 2019	62	24	12 weeks of exergaming vs relaxation and flexibility exercises (control); follow‐up assessed at 24 weeks	Short Physical Performance Battery Test (SPPB)	Scores range from 0 (worst performance) to 12 (best performance); higher scores reflect better performance	Post‐treatment mean (SD) of exergame vs control: 1. SPPB = 9.3 (2.2) vs 9.0 (2.2), P > 0.05	Little to no difference between groups
Physical activity	Karssemeijer 2019	62	24	12 weeks of exergaming vs relaxation and flexibility exercises (control); follow‐up assessed at 24 weeks	Physical Activity Scale for the Elderly (PASE)	PASE score ranges from 0 to 400 or more; higher scores reflect higher levels of physical activity	Post‐treatment mean (SD) of exergame vs control: 1. PASE = 65.5 (55.5) vs 54.2 (45.1), P > 0.05	Little to no difference between groups

N: number; P: probability; SD: standard deviation; vs: versus

For lower limb function and for balance, we found little to no difference between exergaming and control groups of people with dementia at follow‐up.

For general cognition, for memory, and for executive functioning, we found little to no difference between groups. For attention, processing speed, and working memory, findings were mixed.

For functional status and physical performance, and for physical activity, there was little to no difference between groups.

##### Exergaming versus alternative treatment

###### Primary outcomes

Only one study included physical and cognitive functioning outcomes at follow‐up for people with dementia (Karssemeijer 2019). No study assessed ADL outcomes at follow‐up for people with dementia.

Relative to alternative treatment (i.e. treatment with specific effect, in this case, aerobic cycling), we found little to no difference in global physical functioning. For global cognitive functioning, results were mixed.

####### Global physical functioning

Karssemeijer 2019 reported little to no difference in global physical functioning between groups (n = 62). Table 17 provides numerical data, outcome definitions used within the study, and a brief narrative interpretation.

**13:** Analysis 6.1 Exergaming vs alternative treatment at follow‐up for people with dementia: change in global physical functioning

**Study**	**N**	**Follow‐up (weeks)**	**Comparison**	**Physical domain: measure(s)**	**Scale metrics**	**Numerical data**	**Narrative interpretation**
Karssemeijer 2019	62	24	12 weeks of exergaming vs aerobic (alternative treatment); follow‐up assessed at 24 weeks	Lower limb function: 10‐m Walk Test (m/s)	Walking speed in m per second; higher scores reflect better performance	Follow‐up mean (SD) of exergame vs alternative treatment: 1. 10m Walk Test = 1.02 (0.33) vs 1.05 (0.41); 2. TUG = 13.8 (5.5) vs 13.6 (5.3); 3. 5TSST = 15.9 (5.7) vs 16.0 (6.9); 4. FICSIT‐4 = 3.8 (1.1) vs 3.6 (1.3) No between‐group differences (all Ps > 0.05).	Little to no difference between groups
				Lower limb function: Timed Up & Go Test s (TUG)	Time to complete the test; lower scores reflect better performance		
				Lower limb function: Five Times Sit‐to‐stand test (s)	Time to complete the test; lower scores reflect better performance		
				Balance: Frailty and Injuries Cooperative Studies of Interventions Technique Subtest 4 (FICSIT‐4 score)	Score ranges from 0 to 5; higher score reflect better performance		

M: metres; N: number; P: probability; s: seconds; SD: standard deviation; vs: versus

####### Global cognitive functioning

Karssemeijer 2019 reported mixed findings for exergaming vs alternative treatment for global cognitive functioning (n = 62). Table 18 provides numerical data, outcome definitions used within the study, and a brief narrative interpretation.

**14:** Analysis 6.2 Exergaming vs alternative treatment at follow‐up for people with dementia: change in global cognitive functioning

**Study**	**N**	**Follow‐up (weeks)**	**Comparison**	**Cognitive domain: measure(s)**	**Scale metrics**	**Numerical data**	**Narrative interpretation**
Karssemeijer 2019	62	24	12 weeks of exergaming vs aerobic (alternative treatment); follow‐up assessed at 24 weeks	Attention, processing speed, and working memory: Trail Making Test part A (TMT‐A)	Time taken to complete the task; lower scores reflect better performance	Follow‐up mean (SD) of exergame vs alternative treatment: 1. TMT‐A = 55.8 (54.0) vs 38.6 (40.2); 2. Stroop Test word‐reading = 41.1 (16.4) vs 35.7 (18.5); 3. Stroop Test color‐naming = 55.0 (26.1) vs 44.4 (22.8); 4. WAIS‐III Digit Span = 9.6 (3.3) vs 11.0 (3.2); 5. WMS‐III Spatial Span = 7.7 (3.8) vs 9.4 (12.9); 6. LLT 1‐5 =96.1 (34.1) vs 97.5 (41.3); 7. 6. LLT delayed recall = 18.5 (8.1) vs 19.6 (10.5); 8. TMT‐B = 178.1 (105.4) vs 141.1 (96.5); 9. Stroop Test speed‐accuracy trade off scores = 0.34 (0.18) vs 0.32 (0.15); 10. Stroop Test colour‐word interference card = 139.2 (75.2) vs 123.8 (63.5); 11. Stroop Test colour‐word Interference card (number of errors) = 6.1 (8.8) vs.6.6 (7.9); 12. Letter Fluency = 20.9 (11.2) vs 23.5 (12.9); 13. Rule Shift Card Test = 9.2 (5.5) vs 7.6 (4.7) No significant improvement in psychomotor speed for the exergame vs alternative treatment (mean difference domain score (95% CI) − 0.116 (0.399 to – 0.398), p = 0.399). Similarly, the remaining comparisons were not statistically significant.	Mixed findings
				Attention, processing speed, and working memory: Stroop Test word‐reading	Time taken to complete the task; lower scores reflect better performance		
				Attention, processing speed, and working memory: Stroop Test color‐naming	Time taken to complete the task; lower scores reflect better performance		
				Attention, processing speed, and working memory: WAIS‐III Digit Span	Number of digits correctly repeated; higher scores reflect better performance		
				Attention, processing speed, and working memory: WMS‐III Spatial Span	Number of digits correctly repeated; higher scores reflect better performance		
				Memory: Location learning test displacement score	Sum of errors; lower scores reflect better performance		
				Memory: Location learning test displacement score delayed recall	Sum of errors; lower scores reflect better performance		
				Executive functioning: Trail Making Test part B (TMT‐B)	Time taken to complete the task; lower scores reflect better performance		
				Executive functioning: Stroop Test speed‐accuracy trade off scores	Speed‐accuracy trade‐off scores calculated with the formula: (100*accuracy) / reaction time; lower scores reflect better performance		
				Executive functioning: Stroop Test colour‐word interference card	Time taken to complete the task; lower scores reflect better performance		
				Executive functioning: Stroop Test colour‐word Interference card (number of errors)	Number of errors; lower scores reflect better performance		
				Executive functioning: Letter Fluency	Number of correct words generated; higher scores reflect better performance		
				Executive functioning: Rule Shift Card Test	Number of errors; lower scores reflect better performance		

M: metres; N: number; P: probability; s: seconds; SD: standard deviation; vs: versus

####### Activities of daily living

No study assessed ADL outcomes.

###### Secondary outcomes

Narrative results are presented in Table 19.

**15:** Secondary outcomes. Exergaming vs alternative treatment at follow‐up for people with dementia

**Secondary outcome domain**	**Study**	**N**	**Follow‐up (weeks)**	**Comparison**	**Measure(s)**	**Scale metrics**	**Numerical data**	**Narrative interpretation**
Lower limb function	Karssemeijer 2019	62	24	12 weeks of exergaming vs aerobic (alternative treatment); follow‐up assessed at 24 weeks	10‐m Walk Test (m/seconds)	Walking speed in m/seconds; higher scores reflect better performance	Follow‐up mean (SD) of exergame vs alternative treatment: 1. 10m Walk Test = 1.02 (0.33) vs 1.05 (0.41); 2. TUG = 13.8 (5.5) vs 13.6 (5.3); 3. 5TSST = 15.9 (5.7) vs 16.0 (6.9) No between‐group differences (all Ps > 0.05).	Little to no difference between groups
					Timed Up & Go Test s (TUG)	Time to complete the test; lower scores reflect better performance		
					Five Times Sit‐to‐stand test (5TSST) (seconds)	Time to complete the test; lower scores reflect better performance		
Balance	Karssemeijer 2019	62	24	12 weeks of exergaming vs aerobic (alternative treatment); follow‐up assessed at 24 weeks	Frailty and Injuries Cooperative Studies of Interventions Technique Subtest 4 (FICSIT‐4 score)	Score ranges from 0 to 5; higher score reflect better performance	Follow‐up mean (SD) of exergame vs alternative treatment: 1. 4. FICSIT‐4 = 3.8 (1.1) vs 3.6 (1.3) No between‐group differences (all Ps > 0.05).	Little to no difference between groups
Attention, processing speed, and working memory	Karssemeijer 2019	62	24	12 weeks of exergaming vs aerobic (alternative treatment); follow‐up assessed at 24 weeks	Trail Making Test part A (TMT‐A)	Time taken to complete the task; lower scores reflect better performance	Follow‐up mean (SD) of exergame vs alternative treatment: 1. TMT‐A = 55.8 (54.0) vs 38.6 (40.2) 2. Stroop Test word‐reading = 41.1 (16.4) vs 35.7 (18.5); 3. Stroop Test color‐naming = 55.0 (26.1) vs 44.4 (22.8); 4. WAIS‐III Digit Span = 9.6 (3.3) vs 11.0 (3.2); 5. WMS‐III Spatial Span = 7.7 (3.8) vs 9.4 (12.9) No between‐group differences (all Ps > 0.05).	Little to no difference between groups
					Stroop Test word‐reading	Time taken to complete the task; lower scores reflect better performance		
					Stroop Test color‐naming	Time taken to complete the task; lower scores reflect better performance		
					Wechsler Adult Intelligence Scale‐III Digit Span (WAIS‐III Digit Span)	Number of digits correctly repeated; higher scores reflect better performance		
					Wechsler Memory Scale‐III Spatial Span (WMS Digit Span)	Number of digits correctly repeated; higher scores reflect better performance		
Memory	Karssemeijer 2019	62	24	12 weeks of exergaming vs aerobic (alternative treatment); follow‐up assessed at 24 weeks	Location Learning Test Displacement score trial 1 to 5 (LLT 1‐5)	Sum of errors; lower scores reflect better performance	Follow‐up mean (SD) of exergame vs alternative treatment: 1. LLT 1‐5 = 96.1 (34.1) vs 97.5 (41.3) 2. LLT delayed recall = 18.5 (8.1) vs 19.6 (10.5) No between‐group differences (all Ps > 0.05).	Little to no difference between groups
					Location Learning Test Displacement score delayed recall (LLT delayed recall)	Sum of errors; lower scores reflect better performance		
Executive functioning	Karssemeijer 2019	62	24	12 weeks of exergaming vs aerobic (alternative treatment); follow‐up assessed at 24 weeks	Trail Making Test part B (TMT‐B)	Time taken to complete the task; lower scores reflect better performance	Follow‐up mean (SD) of exergame vs alternative treatment: 1. TMT‐B = 178.1 (105.4) vs 141.1 (96.5); 2. Stroop Test speed‐accuracy trade off scores = 0.34 (0.18) vs 0.32 (0.15); 3. Stroop Test colour‐word interference card = 139.2 (75.2) vs 123.8 (63.5); 4. Stroop Test colour‐word Interference card (number of errors) = 6.1 (8.8) vs.6.6 (7.9); 5. Letter Fluency = 20.9 (11.2) vs 23.5 (12.9); 6. Rule Shift Card Test = 9.2 (5.5) vs 7.6 (4.7) No between‐group differences (all Ps > 0.05).	Little to no difference between groups
					Stroop Test speed‐accuracy trade‐off scores	Speed‐accuracy trade‐off scores are calculated with the formula: (100*accuracy) / reaction time; lower scores reflect better performance		
					Stroop Test colour‐word interference card	Time taken to complete the task; lower scores reflect better performance		
					Stroop Test colour‐word interference card (number of errors)	Number of errors; lower scores reflect better performance		
					Letter Fluency	Number of correct words generated; higher scores reflect better performance		
					Rule Shift Card Test	Number of errors; lower scores reflect better performance		
Functional status and physical performance	Karssemeijer 2019	62	24	12 weeks of exergaming vs aerobic (alternative treatment); follow‐up assessed at 24 weeks	Short Physical Performance Battery Test (SPPB)	Scores range from 0 (worst performance) to 12 (best performance); higher scores reflect better performance	Post‐treatment mean (SD) of exergame vs alternative treatment: 1. SPPB = 9.3 (2.2) vs 9.3 (2.4), P > 0.05	Little to no difference between groups
Physical activity	Karssemeijer 2019	62	24	12 weeks of exergaming vs aerobic (alternative treatment); follow‐up assessed at 24 weeks	Physical Activity Scale for the Elderly (PASE)	PASE score ranges from 0 to 400 or more; higher scores reflect higher levels of physical activity	Post‐treatment mean (SD) of exergame vs alternative treatment: 1. PASE = 65.5 (55.5) vs 72.4 (51.4), P > 0.05	Little to no difference between groups

M: metres; N: number; P: probability; s: seconds; SD: standard deviation; vs: versus

For lower limb function and for balance, we found little to no difference between exergaming and alternative treatment groups of people with dementia at follow‐up.

For attention, processing speed, and working memory, for memory, and for executive functioning, we found little to no difference between groups.

For functional status and physical performance, and for physical activity, there was little to no difference between groups.

#### Effects of interventions at follow‐up for people with MCI

##### Exergaming versus control

###### Primary outcomes

Hughes 2014 included physical, cognitive, and ADL outcomes, while Robert 2021 included only cognitive functioning outcomes at follow‐up for people with MCI.

Relative to a control group (i.e. inactive control group such as standard treatment and an active control group such as a healthy ageing education programme), overall there was little to no difference in any of the primary outcomes.

####### Global physical functioning

Hughes 2014 reported little to no difference in global cognitive functioning between groups (n = 19). Table 20 provides numerical data, outcome definitions used within the study, and a brief narrative interpretation.

**16:** Analysis 7.2 Exergaming vs control at follow‐up for people with MCI: change in global physical functioning

**Study**	**N**	**Follow‐up (weeks)**	**Comparison**	**Physical domain: measure(s)**	**Scale metrics**	**Numerical data**	**Narrative interpretation**
Hughes 2014	19	48	24 weeks of exergaming vs healthy aging education programmes (control); follow‐up assessed at 48 weeks	Lower limb function: Gait speed 6‐m walk (m/s)	Time in seconds to complete a 6‐m walk; lower scores reflect better performance	Follow‐up mean (SD) of exergame vs control: 1. 6‐m walk = 7.00 (2.41) vs 7.18 (2.29) Authors reported no between group differences (P > 0.05)	Little to no difference between groups

MCI: mild cognitive impairment; m: metres; N: number; P: probability; s: seconds; SD: standard deviation; vs: versus

####### Global cognitive functioning

We found little to no difference between groups in global cognitive functioning (SMD 0.02, 95% ‐0.62 to 0.67; 2 studies, 38 participants; Analysis 7.1).

####### Activities of daily living

Hughes 2014 reported little to no difference in ADL between groups (n = 19). Table 21 provides numerical data, outcome definitions used within the study, and a brief narrative interpretation.

**17:** Analysis 7.3 Exergaming vs control at follow‐up for people with MCI: change in ADL

**Study**	**N**	**Follow‐up (weeks)**	**Comparison**	**ADL domain: measure(s)**	**Scale metrics**	**Numerical data**	**Narrative interpretation**
Hughes 2014	19	78	24 weeks of exergaming vs healthy aging education programmes (control); follow‐up assessed at 48 weeks	Instrumental activities of daily living: Timed Instrumental Activities of Daily Living (TIADL)	Overall time to complete tasks; lower scores reflect better performance	Follow‐up mean (SD) of exergame vs control: 1. TIADL = 180.60 (99.21) vs 182.46 (86.63) Authors reported no between‐group differences (P > 0.05)	Little to no difference between groups

ADL: activities of daily living; MCI: mild cognitive impairment; N: number; P: probability; SD: standard deviation; vs: versus

###### Secondary outcomes

We pooled results for general cognition. For the remaining secondary outcomes, narrative results are presented in Table 22.

**18:** Secondary outcomes. Exergaming vs control at follow‐up for people with MCI

**Secondary outcome domain**	**Study**	**N**	**Follow‐up (weeks)**	**Comparison**	**Measure(s)**	**Scale metrics**	**Numerical data**	**Narrative interpretation**
Lower limb function	Hughes 2014	19	48	24 weeks of exergaming vs healthy aging education programmes (control); follow‐up assessed at 48 weeks	Gait speed 6‐Meter Walk (m/seconds)	Time in seconds to complete a 6‐meter walk; lower scores reflect better performance	Follow‐up mean (SD) of exergame vs control: 1.6‐m walk = 7.00 (2.41) vs 7.18 (2.29) No between‐group differences (P > 0.05)	Little to no difference between groups
Attention, processing speed, and working memory	Hughes 2014	19	48	24 weeks of exergaming vs healthy aging education programmes (control); follow‐up assessed at 48 weeks	Traking task 1 (connections/second)	Connections per second; higher scores reflect better performance	Follow‐up mean (SD) of exergame vs control: 1. Tracking task 1 = 0.04 (0.02) vs 0.05 (0.02) 2. 1. Tracking task 2 = 0.03 (0.02) vs 0.01 (0.01) No between‐group differences (P > 0.05)	Little to no difference between groups
					Traking task 2 (connections/second)	Connections per second; higher scores reflect better performance		

m: metres; MCI: mild cognitive impairment; N: number; P: probability; SD: standard deviation; vs: versus

We found little to no difference in general cognition between exergaming and control groups of people with MCI at follow‐up (SMD 0.01, 95% ‐0.64 to 0.65; 2 studies, 38 participants; Analysis 7.2).

For lower limb function, we found little to no difference between groups.

We found little to no difference between groups in attention, processing speed, and working memory.

##### Exergaming versus alternative treatment

There was no study that measured the effects of exergames versus alternative treatment at follow‐up for people with MCI.

#### Adverse effects for people with dementia

##### Exergaming versus control

Two studies reported whether there were adverse effects for people with dementia in the exergame and control groups (Karssemeijer 2019; Swinnen 2021). The evidence is very uncertain about the effects of exergaming on adverse effects (0/113 participants). Our certainty was very low because of serious concerns about increased risk of bias and very serious concerns about imprecision due to the small sample size and no events in either group. See Table 23 and Table 1.

**19:** Analysis 8.2 Adverse effects: exergaming vs control for people with dementia

**Study**	**N**	**Adverse effects**	**Narrative interpretation**	**Risk of bias**
Karssemeijer 2019	68	0 adverse effects reported in the intervention and in the control group	No adverse reactions linked to the intervention and control group	Overall risk of bias: some concerns. The study is judged to have low risk of bias in most domains, but there are some concerns for bias due to randomisation process and deviations from intended interventions.
Swinnen 2021	45	0 adverse effects reported in the intervention and in the control group	No adverse reactions linked to the intervention and control group	Overall risk of bias: some concerns. The study is judged to have low risk of bias in most domains, but there are some concerns for bias due to randomisation process and deviations from intended interventions.

N: number; vs: versus

##### Exergaming versus alternative treatment

Two studies reported whether there were adverse effects for people with dementia in the exergame and alternative treatment groups (Karssemeijer 2019; Swinnen 2023). The evidence is very uncertain about the effects of exergaming on adverse effects (RR 7.50, 95% CI 0.41 to 136.52; 2 studies, 2/85 participants; Analysis 8.1). Our certainty was very low because of serious concerns about increased risk of bias and very serious concerns about imprecision. See Table 2.

#### Adverse effects for people with MCI

##### Exergaming versus control

Only one study monitored adverse effects for people with MCI when comparing exergaming with a control group (Manser 2023). The control group in Manser 2023 was usual care. No adverse effects were reported (0/14 participants). The evidence is very uncertain about the effects of exergaming on adverse effects. We judged the certainty of evidence to be very low, downgrading due to serious concerns over risk of bias and very serious concerns about imprecision due to small sample size and no events in either group. See Table 24 and Table 3.

**20:** Analysis 8.3 Adverse effects: exergaming vs control for people with MCI

**Study**	**N**	**Adverse effects**	**Narrative interpretation**	**Risk of bias**
Manser 2023	14	0 adverse effects reported in the intervention and in the control group	No adverse reactions linked to the intervention and control group	Overall risk of bias: some concerns. The study is at low risk in most domains, but there are some concerns in two domains: deviations from intended interventions and selection of reported results.

MCI: mild cognitive impairment; N: number; vs: versus

##### Exergaming versus alternative treatment

Two studies reported whether there were adverse effects for people with MCI when comparing exergames with alternative treatment (Park 2018; Ramnath 2021). The alternative treatments were computerised cognitive training (Park 2018) and standing and sitting exercises (Ramnath 2021). Neither study found any adverse events (0/123 participants). The evidence is very uncertain about the effects of exergaming on adverse effects. We judged the certainty of the evidence to be very low, downgrading due to serious concerns over risk of bias and very serious concerns about imprecision due to small sample size and no events in either group. See Table 25 and Table 4.

**21:** Analysis 8.4 Adverse effects: exergaming vs alternative treatment for people with MCI

**Study**	**N**	**Adverse effects**	**Narrative interpretation**	**Risk of bias**
Park 2018	78	0 adverse effects reported in intervention and in control group	No adverse reactions linked to the intervention and control groups.	Overall risk of bias: some concerns. The study is at low risk in most domains, but there are some concerns in two domains: deviations from intended interventions and selection of reported results.
Ramnath 2021	45	0 adverse effects reported in intervention and in control group	No adverse reactions linked to the intervention and control groups.	Overall risk of bias: some concerns. There are some concerns in most domains.

MCI: mild cognitive impairment; N: number; vs: versus

#### Feasibility and treatment adherence at the end of treatment for people with dementia

##### Exergaming versus control

We included two studies in this analysis (Karssemeijer 2019; Swinnen 2021). Results showed little to no difference between groups for dropout (RR 0.89, 95% CI 0.39 to 2.05; 2 studies, 19/132 participants; Analysis 9.1).

##### Exergaming versus alternative treatment

We included two studies in this analysis (Karssemeijer 2019; Swinnen 2023). Results showed little to no difference between groups for dropout (RR 0.88, 95% CI 0.33 to 2.32; 2 studies, 14/99 participants; Analysis 9.2).

#### Feasibility and treatment adherence at the end of treatment for people with MCI

##### Exergaming versus control

We included three studies in this analysis (Hughes 2014; Lee 2021; Manser 2023). Hughes 2014 and Lee 2021 reported no dropouts at the end of treatment for either of the two groups. Manser 2023 reported 2/10 participants lost in the exergame group and 0/6 participants lost in the control group. Reasons for withdrawal were lack of motivation and surgery not related to the intervention. Results showed little to no difference in dropout rate (RR 3.18, 95% CI 0.18 to 56.95; 3 studies, 2/76 participants; Analysis 9.3).

##### Exergaming versus alternative treatment

We included four studies in this analysis (Arshad 2023; Park 2018; Ramnath 2021; Torpil 2020). Results showed little to no difference between groups in dropout rate (RR 1.5, 95% CI 0.45 to 4.99; 4 studies, 10/243 participants; Analysis 9.4).

#### Feasibility and treatment adherence at follow‐up for people with dementia

##### Exergaming versus control

We included two studies in this analysis (Karssemeijer 2019; Robert 2021). Results showed little to no difference in dropout rate at follow‐up between the exergame and control groups (RR 0.99, 95% CI 0.48 to 2.04; 2 studies, 23/144 participants; Analysis 9.5).

##### Exergaming versus alternative treatment

Only one study of 76 participants was available for inclusion (Karssemeijer 2019). There were 2/38 participants lost to follow‐up in the exergame group and 4/39 lost in the alternative treatment group at follow‐up, for similar reasons, including hospital admissions, death of partner, refusal, holiday, and cancellation.

#### Feasibility and treatment adherence at follow‐up for people with MCI

##### Exergaming versus control

We included two studies in the analysis (Hughes 2014; Robert 2021). Results showed little to no difference between groups in dropout rate (RR 0.24, 95% CI 0.03 to 1.93; 2 studies, 5/43 participants; Analysis 9.6).

##### Exergaming versus alternative treatment

There was no study that measured the effects of exergames versus alternative treatment at follow‐up for people with MCI.

## Discussion

### People with dementia

#### Exergaming versus control

For people with dementia, we found that exergaming may increase global cognitive functioning at the end of treatment, but the evidence is very uncertain. At follow‐up, the evidence is very uncertain about the effects of exergaming on global cognitive functioning.

Exergaming may result in little to no difference in global physical functioning and ADL at the end of treatment, but the evidence is very uncertain. Similarly, at follow‐up, we found little to no difference in global physical functioning. No study included ADL outcomes at follow‐up.

The evidence is very uncertain about the effects of exergaming on adverse effects. There may be little to no difference between exergaming and control interventions in the number of dropouts at the end of treatment and follow‐up.

#### Exergaming versus alternative treatment

Exergaming may have little to no effect on global physical and cognitive functioning at the end of treatment, but the evidence is very uncertain. Exergaming may not increase ADL at the end of treatment.

At follow‐up, exergaming may result in little to no difference in global physical functioning. Similarly, at follow‐up, there are mixed findings for global physical functioning. No study included ADL outcomes at follow‐up.

The evidence is very uncertain about the effects of exergaming on adverse effects. There may be little to no difference between exergaming and alternative treatment interventions in the number of participants who have dropped out by the end of treatment and follow‐up.

### People with MCI

#### Exergaming versus control

For people with MCI, we found that exergaming may increase global cognitive functioning at the end of treatment, but the evidence is very uncertain. Concerning the effects of exergaming at follow‐up, the evidence is very uncertain for global cognitive functioning.

Exergaming may result in little to no difference in global physical functioning and ADL at the end of treatment, but the evidence is very uncertain. Similarly, at follow‐up, there is little to no difference in global physical functioning and ADL.

The evidence is very uncertain about the effects of exergaming on adverse effects. There may be little to no difference between exergaming and control interventions in the number of dropouts at the end of treatment and follow‐up.

#### Exergaming versus alternative treatment

Exergaming may have little to no effect on cognitive functioning at the end of treatment, but the evidence is very uncertain. The evidence suggests that exergaming versus alternative treatment may result in little to no difference in global physical functioning. No study included ADL outcomes.

There was no study that measured the effects of exergaming versus alternative treatment at follow‐up for people with MCI.

The evidence is very uncertain about the effects of exergaming on adverse effects. There may be little to no difference between exergaming and alternative treatment interventions in the number of dropouts at the end of treatment. No study was available for inclusion at follow‐up.

### Overall completeness and applicability of evidence

We identified 11 studies that were eligible for inclusion in this review. Seven studies included people with MCI, and three studies included people with dementia (one study included both, providing separate data for people with MCI and people with dementia). In total, we had data from 328 participants with MCI and 214 people with dementia. Ten studies assessed the effects of exergaming at the end of treatment; only two of those studies assessed the effects at a later follow‐up time point.

There were few studies to include per comparison and outcome because we looked separately at exergaming versus control (i.e. no treatment, standard treatment, waiting list, or non‐specific active control), and exergaming versus alternative treatment. For example, for people with dementia, only three studies compared exergaming with control groups and two studies with alternative treatment. For people with MCI, three studies included a control group and four studies had an alternative treatment comparator. Moreover, not every study included all of our primary outcomes. For some comparisons and outcomes, it was not meaningful to pool results due to statistical heterogeneity.

The small number of studies available for inclusion for each comparison, together with the small sample sizes, make it difficult to generalise from the results. This is also reflected in the very low or low certainty of the evidence for all comparisons as assessed according to GRADE. Though it is important to highlight that the lack of evidence of benefit does not necessarily equate to a lack of benefit. This applies to all our findings, meaning that the evidence around the effects exergaming is inconclusive.

Due to having too few studies, we were unable to investigate the effects of moderators by conducting subgroup analyses. For dementia severity, one study included people with mild dementia (Karssemeijer 2019), and two studies included participants with moderate dementia (Robert 2021; Swinnen 2021). One study reported mean scores on the MMSE that correspond to moderate severity of dementia (Swinnen 2023). The same study reported means scores for the MoCA that correspond to severe dementia (Swinnen 2023).

To explore the combined benefits of physical activity and cognitive training in exergaming, we aimed to compare studies using physical activity alone versus those incorporating both physical activity and cognitive tasks. However, because of small sample size, we could not perform planned subgroup analyses. In the studies of exergame interventions for people with dementia, the exergames combined physical activity with cognitive training tasks. As a consequence, we cannot generalise the effects of this type of exergaming platform to exergaming platforms that rely on physical exercises only. Most commercial platforms such as Microsoft Xbox or Nintendo Wii involve mainly or only physical exercises. For people with MCI, three studies delivered exergaming interventions that combined physical activity with cognitive training tasks (Arshad 2023; Manser 2023; Robert 2021). Other studies employed exergame interventions exclusively featuring physical activity tasks (Hughes 2014; Lee 2021; Park 2018; Ramnath 2021; Torpil 2020). Because of this, we have concerns that, especially for MCI, it may be more difficult to isolate the effects of exergaming interventions with or without additional cognitive training elements.

In terms of the type of control intervention, only three studies included an inactive control group (i.e. no intervention or treatment as usual) and six studies included forms of active control groups (i.e. intervention involving equivalent contact with the researchers but not hypothesised to have any specific effect on the study outcomes). One study with an inactive control group performed measurements only at follow‐up (Robert 2021). Limited data resulted in fewer than the desired number of studies available for subgroup comparisons.

All platforms designed for people with dementia were customised. All but one of the studies involving people with MCI used commercial platforms. All studies used monitor display technology, and none used immersive technologies and HMDs such as the Oculus Rift, HTC VIVE, or CAVE environments.

In terms of intervention length, subgroup comparisons were not possible as the total length of the intervention was less than 360 minutes (6 hours) in only two studies (Robert 2021; Swinnen 2021). One study had a duration of up to 12 hours (361 to 720 minutes) (Lee 2021). The remaining studies had an intervention duration of more than 720 minutes.

An insufficient number of studies were available to conduct subgroup analyses for length of follow‐up period, with only two studies reporting follow‐up data for people with MCI (48 weeks, Hughes 2014; 24 weeks, Robert 2021), and two for people with dementia (24 weeks, Karssemeijer 2019; 24 weeks, Robert 2021).

We concluded that the evidence is very uncertain about the effects of exergaming on adverse effects. In every study that included and reported adverse effects, the exergaming sessions were supervised by a healthcare professional. A key benefit of the use of exergaming as an intervention may be the possibility of delivering it in a home environment, unsupervised. Only two studies delivered the exergaming intervention in a home environment and the sessions were supervised. Due to the above, we consider that there is no robust evidence on which to base a judgement of the safety of unsupervised exergaming in a home environment for people with MCI or dementia.

### Quality of the evidence

Our GRADE assessments for each comparison can be found in the Effects of interventions (in text) and in summary of findings tables: Table 1; Table 2; Table 3; Table 4.

Based on GRADE criteria, we considered the certainty of evidence across all comparisons to be very low or low, indicating that our confidence in the effect estimate is very limited. We identified issues of risk of bias, imprecision, and inconsistency.

For cluster‐RCTs, we downgraded for by one level for imprecision due to the small number of participants. The effect estimates for these cluster‐RCTs were of very low and low certainty of evidence, which limits our confidence in the effect estimates and indicates that the true effects are likely to be substantially different from the estimates of the effects we found in this review.

For most comparisons, using GRADE criteria, we downgraded by one level for risk of bias because the evidence came from studies at high risk or with some concerns of bias; we downgraded for inconsistency as the directions of effects were inconsistent across studies, or due to considerable heterogeneity that could not be explained; for imprecision, we downgraded as the confidence interval included both appreciable harm and appreciable benefit (i.e. 95% CI spans 0), and due to the small numbers of participants (N < 400). For imprecision, we downgraded all studies by at least one level as all included less than 400 participants, a threshold that can be difficult to achieve with new technologies such as VR or exergame studies. For example, most studies in neuroscience are underpowered (Button 2013). We did not downgrade for indirectness as there was no evidence of it. For all comparisons, we did not downgrade for reporting bias as we set the minimum threshold for examining this at 10 trials per comparison, which was not met.

We conclude that, overall, the evidence for the effectiveness of exergaming for people with dementia and MCI is inconclusive.

### Potential biases in the review process

We intended to assess reporting biases using funnel plots and trim‐and‐fill procedure. However, this was not possible due to the small number of studies providing outcome data in the review.

Following our protocol, we applied rigorous participant eligibility criteria and only included studies where the participants were categorised based on well‐known diagnostic criteria for both dementia and MCI. We therefore excluded 17 studies that did not appear to match our diagnostic eligibility criteria.

We conducted the electronic searches without language and date restrictions, and we aimed to identify all relevant studies. For example, we contacted all the authors of ongoing studies and study protocols that could match our eligibility criteria on at least two occasions. However, the response rate was very low, and we were not able to include studies where we did not receive any response or data. Instead, these were listed in the ongoing studies or studies awaiting classification sections. Despite trying to gather all available evidence, there may be bias in failing to include all the available data because they were still being collected or under review, or studies were at halt due to Covid restrictions. When we were unsure about the eligibility of the population according to our protocol, we contacted study authors to clarify the dementia or MCI diagnostic criteria used in their studies. From 12 studies, we got seven replies. For five studies, we did not get a reply to judge eligibility criteria; therefore, we were conservative and excluded these studies (see Characteristics of excluded studies).

We aimed to perform several sensitivity analyses. First, we aimed to compare studies at high risk versus low risk of bias; but because all comparisons were judged to be at high risk or with some concerns, we were not able to perform these sensitivity analyses. We also aimed to compare the results of dose‐matched studies (equal dose of time and frequency) versus non‐dose‐matched studies, but we judged there to be an insufficient number of studies to run sensitivity analysis. All studies except Lee 2021, Manser 2023 and Robert 2021 administered control and exergame interventions with an equal dose of time and frequency. We also planned to compare the results of studies with high statistical power (> 0.80) and studies with low statistical power. For these, the average post‐hoc power of 80% was achieved in case of Arshad 2023 for cognitive functioning, Lee 2021 for ADL, Swinnen 2021 for physical and cognitive functioning, and Torpil 2020 for cognitive functioning. We did not run the sensitivity analyses due to the small number of observations, or because we were unable to pool results meaningfully due to heterogeneity.

We stated we would include only exergaming interventions of moderate intensity. The level of energy expenditure varies on a continuum from low to high (Caspersen 1985). We used published guidelines to identify levels of intensity of physical activity (Norton 2010). Norton 2010 proposed the following categories of intensity, based on energy expenditure: sedentary **–** sitting or lying with low additional movement (e.g. watching TV or riding a car), and low energy requirement; light **–** an aerobic activity that does not cause a noticeable change in breathing rate, can be sustained for at least 60 minutes (e.g. domestic or occupational tasks, such as ironing, washing, cooking, eating); moderate **–** an aerobic activity that can be performed while maintaining an uninterrupted conversation, may last between 30 and 60 minutes (e.g. gentle swimming, social tennis, golf, cycling at regular pace, carrying light loads); high **–** an aerobic activity that cannot sustain a conversation without interruption, and can last for about 30 minutes (e.g. jogging, cycling, aerobics, competitive tennis); very high **–** an aerobic activity that cannot be sustained for longer than about 10 minutes, and has a relative intensity level of at least 90% maximum heart rate; but such activities rarely occur in daily life. We only considered interventions of at least moderate intensity. We were unable to use objective criteria (e.g. heart rate or energy consumption) to identify if the interventions were of moderate intensity as this was not reported in the studies and relied on the guidelines proposed by Norton 2010. Results of a meta‐analysis showed that exergaming may facilitate light‐ to moderate‐intensity physical activity (Peng 2011).

We also included two cluster‐RCTs in this review. For these, we did not follow specific procedures to avoid a unit‐of‐analysis error. Both studies adjusted for clustering (Ramnath 2021; Robert 2021). We used appropriate risk of bias tool 2 for cluster‐RCTs.

We have documented and justified modifications to our published protocol in the Differences between protocol and review section of this review.

### Agreements and disagreements with other studies or reviews

A relatively small body of work has been published on the topic of the effectiveness of exergame interventions for improving physical, cognitive, and ADL outcomes for people with MCI and dementia.

We identified three systematic reviews and one meta‐analysis that specifically assessed the effects of exergames among people with dementia and people with MCI.

Cai 2023 performed a meta‐analysis to assess the effects of exergaming on cognitive function, physical performance, and ADL in people with MCI and dementia. Eight studies were included. Results were mixed. For some cognitive function outcomes, exergaming gave some improvements of limited clinical importance, but this was not found for physical functioning outcomes and ADL. The risk of bias in the included studies was assessed with the Cochrane RoB 1 tool, with studies mostly at low risk of bias. The analysis included both people with MCI and dementia, and did not discriminate between the two categories of population. For the control group, the authors included studies with a control group that underwent physical activity or continued their usual care.

Swinnen 2022 conducted a systematic review with eight studies to assess the effects of exergaming on physical and cognitive functioning outcomes, and ADL in people with major neurocognitive disorder. Overall, the authors report that exergames may result in improvements in cognitive and physical functioning, but not ADL. The methodological study quality was assessed using the PEDro tool and for non‐randomised studies with Robins‐I tool. Seven studies were rated as having high methodological quality. The GRADE assessment revealed four studies with moderate quality, and four studies having high methodological quality. In this review, not all participants had a clinical diagnosis of dementia based on DSM or other established criteria. The type of control group was not considered (e.g. waitlist, standard care, alternative treatment).

Zhao 2020 included 10 studies that compared exergames versus no intervention or active or alternative interventions, on physical and cognitive outcomes, among both people with dementia and MCI. This review found no differences in physical functioning and cognitive functioning. They did not include data on ADL. The authors report high heterogeneity in terms of the duration, frequency, and gaming platform used. Quality was judged to be moderate to high and was assessed with the Cochrane RoB 1 tool for RCTs and the ROBINS‐I tool for quasi‐RCTs. The review did not discriminate between types of control groups or populations with MCI or dementia.

Van Santen 2018 focused on the effects of exergame compared with active or alternative interventions on physical, cognitive, emotional, social functioning, and quality of life of people living with dementia. Three studies were included, with mixed results.

As we can see, there is some overlap between the current review and these recent reviewes, although the comparison is complicated by differences in classifying interventions, type of control group (e.g. passive or active controls versus alternative treatment), and type of population (e.g. dementia versus MCI). In our review, we conducted separate comparisons depending on the type of control group (e.g. control versus alternative treatment) and population type (i.e. dementia versus MCI). There are also differences related to how risk of bias and the quality or certainty of the evidence was assessed. We assessed risk of bias with the latest version of the Cochrane RoB 2 tool and assessed the certainty of the evidence with GRADE.

In general, none of the above reviews included or defined exergames based on energy expenditure; nor did they apply rigorous criteria to define the population that was included (e.g. diagnostic criteria). Results were very heterogeneous and difficult to collate. All reviews were pre‐registered. Only Swinnen 2022 included adverse effects and feasibility outcomes, reporting no major adverse effects and high adherence for both groups. We agree with Cai 2023, Swinnen 2022 and Zhao 2020 when they consider the high heterogeneity as a limitation in the evidence base, for example, in terms of exergame hardware and software, duration of studies, length of follow‐up, and mode of delivery, such as individual and group sessions. We identified considerable heterogeneity across some comparisons, and we were not able to explore this as there were not enough studies in each category to perform additional subgroup analyses. We are also in agreement with Cai 2023, Van Santen 2018 and Zhao 2020 when they discuss limitations due to small sample sizes. In our review, the small sample size affected our confidence in results and we downgraded the certainty of the evidence based on this.

Overall, similar to the other reviews published, our results are impacted by the small sample size, low power, and the considerable heterogeneity in the implementation of exergaming interventions. This may impact the conclusions we can draw from this review, which are based on an insufficient number of studies available in this area.

## Authors' conclusions

**Implications for practice**

There may be some benefits from exergaming for global cognitive functioning when compared with a control group (i.e. usual treatment or non‐specific active control) for people with dementia and people with mild cognitive impairment (MCI), but we have very low certainty about this evidence, and whether any benefit persists in the long term is unclear. The evidence is very uncertain about the effect of exergaming relative to alternative treatment (e.g. treatment with specific effects such as physical activity) on cognitive functioning for people with dementia or MCI.

Although exergaming is proposed to improve physical functioning and activities of daily living (ADL), there is currently very uncertain evidence about its use amongst people with dementia and MCI. Similarly, the evidence is very uncertain about the effect of exergaming on physical, cognitive, and ADL outcomes at follow‐up.

There is very uncertain evidence about the safety of exergaming in sessions supervised by a healthcare professional that take place in a controlled environment. We have no evidence about the safety of unsupervised exergaming in a home environment.

Adherence rates were similar between the exergames and all control groups, suggesting that exergaming is as feasible as the active or alternative interventions tested. The studies were conducted in Europe, the USA, the Republic of Korea, Pakistan, and Turkey, which suggests their cross‐cultural applicability.

Because our results are based on a small number of studies, with low power and high heterogeneity, we consider that there are insufficient data to allow us to draw definitive conclusions concerning the effectiveness of exergaming in dementia and MCI for improving physical, cognitive, and ADL outcomes. Our findings are based on very‐low and low certainty of evidence because of the impact of low sample sizes and substantial heterogeneity. This resulted in a lack of reliable evidence to support exergaming, as highlighted throughout this review. However, we emphasise that the absence of evidence does not necessarily imply the absence of benefit.

**Implications for research**

Most of the evidence comes from studies that used small samples that were underpowered to detect effects (e.g. most of the comparisons achieved a post‐hoc statistical power less than the threshold of 80%). Large, multicentre randomised trials of exergaming interventions are needed, which have multiple assessment points for physical, cognitive, and ADL outcomes, including long‐term follow‐up. Including multiple time‐point assessments and adequate follow‐up measurements would enable understanding of the long‐term effects of exergaming, and adequate sample sizes would improve power and precision.

There was high heterogeneity in interventions and control conditions, type of exergame platform, length of interventions, and follow‐up duration; these are some of the variables that could impact treatment outcomes and that make it difficult to generalise results. There are some aspects that remain understudied, such as types of virtual reality (VR) technology. No study from the current review focused on the use of head‐mounted displays (HMDs) such as the Oculus or HTC Vive or CAVE environments, and all studies used monitor display platforms such as projectors or big TV screens. We are aware that when establishing the evidence for a novel healthcare intervention such as exergaming, studies are first conducted in a controlled environment such as laboratories and healthcare centres; however, certainty concerning safety use can only be achieved if studies are conducted "in the wild", in participants' homes, for example, and unsupervised.

Other variables such as participant origin (e.g. whether recruited from memory clinics or community) and delivery setting (e.g. community centre, home, memory clinic, retirement home) could also impact effectiveness and adherence. Motivational factors and economic variables were out of scope for this review; however, future studies could focus on investigating their effects.

Finally, more information concerning the randomisation process, including allocation concealment and group differences at baseline, coupled with blinding of outcome assessors, would allow for increased certainty of evidence in the results and recommendations.

## History

Protocol first published: Issue 1, 2021

## Risk of bias

**Table:** Risk of bias for analysis 1.1 Exergaming vs control at the end of treatment: change in global physical functioning (composite)

**Study**	**Bias**											
	**Randomisation process**		**Deviations from intended interventions**		**Missing outcome data**		**Measurement of the outcome**		**Selection of the reported results**		**Overall**	
	**Authors' judgement**	**Support for judgement**	**Authors' judgement**	**Support for judgement**	**Authors' judgement**	**Support for judgement**	**Authors' judgement**	**Support for judgement**	**Authors' judgement**	**Support for judgement**	**Authors' judgement**	**Support for judgement**
Karssemeijer 2019	Some concerns	There is evidence of random allocation but there is no information of allocation concealment. There is no information about significant baseline differences between the groups.	Some concerns	Comment: Blinding of participants was not possible due to the nature of the intervention and there is no information about blinding of carers or those delivering the intervention, but it is assumed the same limitations apply. There is no information about deviations from intended interventions or of moving participants after allocation.	Low risk of bias	Comment: Dropout was 13% in the control and 11% in the exergame condition, which is above the 5% threshold. A sensitivity analysis was performed and suggested similar results indicating that the findings were robust.	High risk of bias	Comment: Used standardised measurements and were the same for both intervention and control groups. Outcome assessors were not blinded.	Low risk of bias	Comment: Data were analysed according to the pre‐registered protocol. The outcomes and analyses were pre‐registered in the protocol.	High risk of bias	Comment: There are some or high concerns in most domains.
Swinnen 2021	Some concerns	Comment: Report of randomisation procedures but no information about allocation concealment. There were no baseline differences between groups.	Some concerns	Comment: No information about blinding of participants or carers/ those delivering the intervention, but it is assumed that it was not possible due to the nature of the intervention (i.e., participants see what they do). There is no information about deviations from intended interventions or of moving participants after allocation.	Low risk of bias	Comment: There is no report of sensitivity analysis or any other methods to correct for potential bias, but dropout rate and reasons were the same for the intervention and control groups.	Low risk of bias	Comment: Used standardised measurements and the scales and timing of outcomes was the same for both intervention and control groups. Assessors were blinded to treatment allocation.	Low risk of bias	Comment: A protocol was published without enough information about the data analysis plan, but there is no indication for selecting specific outcomes based on the results or the analyses.	Some concerns	Comment: The study is judged to have low risk of bias in most domains but there are some concerns for bias due to randomisation process and deviations from intended interventions.

**Table:** Risk of bias for analysis 1.2 Exergaming vs control at the end of treatment: change in global cognitive functioning (composite)

**Study**	**Bias**											
	**Randomisation process**		**Deviations from intended interventions**		**Missing outcome data**		**Measurement of the outcome**		**Selection of the reported results**		**Overall**	
	**Authors' judgement**	**Support for judgement**	**Authors' judgement**	**Support for judgement**	**Authors' judgement**	**Support for judgement**	**Authors' judgement**	**Support for judgement**	**Authors' judgement**	**Support for judgement**	**Authors' judgement**	**Support for judgement**
Karssemeijer 2019	Some concerns	There is evidence of random allocation but there is no information of allocation concealment. There is no information about significant baseline differences between the groups.	Some concerns	Comment: Blinding of participants was not possible due to the nature of the intervention and there is no information about blinding of carers or those delivering the intervention, but it is assumed the same limitations apply. There is no information about deviations from intended interventions or of moving participants after allocation.	Low risk of bias	Comment: Dropout was 13% in the control and 11% in the exergame condition, which is above the 5% threshold. A sensitivity analysis was performed and suggested similar results indicating that the findings were robust.	Low risk of bias	Comment: Used standardised measurements and were the same for both intervention and control groups. Outcome assessors were blinded.	Low risk of bias	Comment: Data were analysed according to the pre‐registered protocol. The outcomes and analyses were pre‐registered in the protocol.	Some concerns	The study is judged to have low risk of bias in most domains but there are some concerns for bias due to randomisation process and deviations from intended interventions.
Swinnen 2021	Some concerns	Comment: Report of randomisation procedures but no information about allocation concealment. There were no baseline differences between groups.	Some concerns	Comment: No information about blinding of participants or carers/ those delivering the intervention, but it is assumed that it was not possible due to the nature of the intervention (i.e., participants see what they do). There is no information about deviations from intended interventions or of moving participants after allocation.	Low risk of bias	Comment: There is no report of sensitivity analysis or any other methods to correct for potential bias, but dropout rate and reasons were the same for the intervention and control groups.	Low risk of bias	Comment: Used standardised measurements and the scales and timing of outcomes was the same for both intervention and control groups. Assessors were blinded to treatment allocation.	Low risk of bias	Comment: A protocol was published without enough information about the data analysis plan, but there is no indication for selecting specific outcomes based on the results or the analyses.	Some concerns	Comment: The study is judged to have low risk of bias in most domains but there are some concerns for bias due to randomisation process and deviations from intended interventions.

**Table:** Risk of bias for analysis 1.3 Exergaming vs control at the end of treatment: change in activities for daily living (ADL)

**Study**	**Bias**											
	**Randomisation process**		**Deviations from intended interventions**		**Missing outcome data**		**Measurement of the outcome**		**Selection of the reported results**		**Overall**	
	**Authors' judgement**	**Support for judgement**	**Authors' judgement**	**Support for judgement**	**Authors' judgement**	**Support for judgement**	**Authors' judgement**	**Support for judgement**	**Authors' judgement**	**Support for judgement**	**Authors' judgement**	**Support for judgement**
Karssemeijer 2019	Some concerns	There is evidence of random allocation but there is no information of allocation concealment. There is no information about significant baseline differences between the groups.	Some concerns	Comment: Blinding of participants was not possible due to the nature of the intervention and there is no information about blinding of carers or those delivering the intervention, but it is assumed the same limitations apply. There is no information about deviations from intended interventions or of moving participants after allocation.	Low risk of bias	Comment: Dropout was 13% in the control and 11% in the exergame condition, which is above the 5% threshold. A sensitivity analysis was performed and suggested similar results indicating that the findings were robust.	High risk of bias	Comment: Used standardised measurements and were the same for both intervention and control groups. Outcome assessors were not blinded.	Low risk of bias	Comment: Data were analysed according to the pre‐registered protocol. The outcomes and analyses were pre‐registered in the protocol.	High risk of bias	Comment: There are some or high concerns in most domains.
Swinnen 2021	Some concerns	Comment: Report of randomisation procedures but no information about allocation concealment. There were no baseline differences between groups.	Some concerns	Comment: No information about blinding of participants or carers/ those delivering the intervention, but it is assumed that it was not possible due to the nature of the intervention (i.e., participants see what they do). There was no information about deviations from intended interventions or about moving participants after the allocation.	Low risk of bias	Comment: There is no report of sensitivity analysis or any other methods to correct for potential bias, but dropout rate and reasons were the same for the intervention and control groups.	Low risk of bias	Comment: Used standardised measurements and the scales and timing of outcomes was the same for both intervention and control groups. Assessors were blinded to treatment allocation.	Low risk of bias	Comment: A protocol was published without enough information about the data analysis plan, but there is no indication for selecting specific outcomes based on the results or the analyses.	Some concerns	Comment: The study is judged to have low risk of bias in most domains but there are some concerns for bias due to randomisation process and deviations from intended interventions.

**Table:** Risk of bias for analysis 2.1 Exergaming vs alternative treatment at the end of treatment: change in global physical functioning (composite)

**Study**	**Bias**											
	**Randomisation process**		**Deviations from intended interventions**		**Missing outcome data**		**Measurement of the outcome**		**Selection of the reported results**		**Overall**	
	**Authors' judgement**	**Support for judgement**	**Authors' judgement**	**Support for judgement**	**Authors' judgement**	**Support for judgement**	**Authors' judgement**	**Support for judgement**	**Authors' judgement**	**Support for judgement**	**Authors' judgement**	**Support for judgement**
Karssemeijer 2019	Some concerns	There is evidence of random allocation but there is no information of allocation concealment. There is no information about significant baseline differences between the groups.	Some concerns	Comment: Blinding of participants was not possible due to the nature of the intervention and there is no information about blinding of carers or those delivering the intervention, but it is assumed the same limitations apply. There is no information about deviations from intended interventions or of moving participants after allocation.	Low risk of bias	Comment: Dropout was 13% in the control and 11% in the exergame condition, which is above the 5% threshold. A sensitivity analysis was performed and suggested similar results indicating that the findings were robust.	High risk of bias	Comment: Used standardised measurements and were the same for both intervention and control groups. Outcome assessors were not blinded.	Low risk of bias	Comment: Data were analysed according to the pre‐registered protocol. The outcomes and analyses were pre‐registered in the protocol.	High risk of bias	Comment: There are some or high concerns in most domains.
Swinnen 2023	Some concerns	Comment: Report of randomisation procedures but no information about allocation concealment. There were no baseline differences between groups.	Some concerns	Comment: Participants and people delivering the interventions were not blinded. There is no information about deviations from intended interventions or of moving participants after allocation.	Low risk of bias	Comment: There is no report of sensitivity analysis or any other methods to correct for potential bias, but dropout rate and reasons were the same for the intervention and control groups.	High risk of bias	Comment: Used standardised measurements and were the same for both intervention and control groups. There is no information about blinding of outcome assessors.	Low risk of bias	Comment: A protocol was published without enough information about the data analysis plan, but there is no indication for selecting specific outcomes based on the results or the analyses.	High risk of bias	Comment: There are some or high concerns in most domains.

**Table:** Risk of bias for analysis 2.3 Exergaming vs alternative treatment at the end of treatment: change in global cognitive functioning (composite)

**Study**	**Bias**											
	**Randomisation process**		**Deviations from intended interventions**		**Missing outcome data**		**Measurement of the outcome**		**Selection of the reported results**		**Overall**	
	**Authors' judgement**	**Support for judgement**	**Authors' judgement**	**Support for judgement**	**Authors' judgement**	**Support for judgement**	**Authors' judgement**	**Support for judgement**	**Authors' judgement**	**Support for judgement**	**Authors' judgement**	**Support for judgement**
Karssemeijer 2019	Some concerns	There is evidence of random allocation but there is no information of allocation concealment. There is no information about significant baseline differences between the groups.	Some concerns	Comment: Blinding of participants was not possible due to the nature of the intervention and there is no information about blinding of carers or those delivering the intervention, but it is assumed the same limitations apply. There is no information about deviations from intended interventions or of moving participants after allocation.	Low risk of bias	Comment: Dropout was 13% in the control and 11% in the exergame condition, which is above the 5% threshold. A sensitivity analysis was performed and suggested similar results indicating that the findings were robust.	Low risk of bias	Comment: Used standardised measurements and were the same for both intervention and control groups. Outcome assessors were blinded.	Low risk of bias	Comment: Data were analysed according to the pre‐registered protocol. The outcomes and analyses were pre‐registered in the protocol.	Some concerns	The study is judged to have low risk of bias in most domains but there are some concerns for bias due to randomisation process and deviations from intended interventions.
Swinnen 2023	Some concerns	Comment: Report of randomisation procedures but no information about allocation concealment. There were no baseline differences between groups.	Some concerns	Comment: Participants and people delivering the interventions were not blinded. There is no information about deviations from intended interventions or of moving participants after allocation.	Low risk of bias	Comment: There is no report of sensitivity analysis or any other methods to correct for potential bias, but dropout rate and reasons were the same for the intervention and control groups.	High risk of bias	Comment: Used standardised measurements and were the same for both intervention and control groups. There is no information about blinding of outcome assessors.	Low risk of bias	Comment: A protocol was published without enough information about the data analysis plan, but there is no indication for selecting specific outcomes based on the results or the analyses.	High risk of bias	Comment: There are some or high concerns in most domains.

**Table:** Risk of bias for analysis 3.1 Exergaming vs control at the end of treatment: change in global physical functioning (composite)

**Study**	**Bias**											
	**Randomisation process**		**Deviations from intended interventions**		**Missing outcome data**		**Measurement of the outcome**		**Selection of the reported results**		**Overall**	
	**Authors' judgement**	**Support for judgement**	**Authors' judgement**	**Support for judgement**	**Authors' judgement**	**Support for judgement**	**Authors' judgement**	**Support for judgement**	**Authors' judgement**	**Support for judgement**	**Authors' judgement**	**Support for judgement**
Hughes 2014	Some concerns	Comment: Participants were randomly assigned to the intervention and control group using generated random numbers. Allocation concealment was not described. There were no baseline differences between groups.	Some concerns	Comment: No information about blinding of participants or carers/ those delivering the intervention, but it is assumed that it was not possible due to the nature of the intervention (i.e., participants see what they do). There is no information about deviations from intended interventions or of moving participants after allocation.	Low risk of bias	Comment: There were no dropouts at the end of treatment and all participants were included in the data analysis.	High risk of bias	Comment: Used of standardised measurements and scales and timepoints of measurement were the same for the intervention and control group. No information about the blinding of outcome assessors was provided. Bias due to no blinding could have influenced the outcomes.	Some concerns	Comment: No protocol is reported, so it is not possible to assess if there was selection of reported outcomes based on the results or the analyses.	High risk of bias	Comment: There are some concerns or high risk of bias in most domains.
Manser 2023	Low risk of bias	Comment: Report of randomisation procedures and allocation concealment. No significant differences between groups at baseline.	Some concerns	Comment: Due to the nature of the intervention blinding was not possible. There is no information about deviations from intended interventions or of moving participants after allocation.	Low risk of bias	Comment: Dropout rates were above (20%) the threshold (5%) for the intervention group. A sensitivity analysis was performed and suggested similar results indicating that the findings were robust.	High risk of bias	Comment: Used standardised measurements and were the same for both intervention and control groups. Outcome assessors were not blinded.	Some concerns	Comment: A protocol was published retrospectively and based on the information provided in the clinical trial registration, it was not possible to assess if data was analysed accordingly.	High risk of bias	Comment: There are some concerns or high risk of bias in most domains.

**Table:** Risk of bias for analysis 3.2 Exergaming vs control at the end of treatment: change in global cognitive functioning (composite)

**Study**	**Bias**											
	**Randomisation process**		**Deviations from intended interventions**		**Missing outcome data**		**Measurement of the outcome**		**Selection of the reported results**		**Overall**	
	**Authors' judgement**	**Support for judgement**	**Authors' judgement**	**Support for judgement**	**Authors' judgement**	**Support for judgement**	**Authors' judgement**	**Support for judgement**	**Authors' judgement**	**Support for judgement**	**Authors' judgement**	**Support for judgement**
Hughes 2014	Some concerns	Comment: Participants were randomly assigned to the intervention and control group using generated random numbers. Allocation concealment was not described. There were no baseline differences between groups.	Some concerns	Comment: No information about blinding of participants or carers/ those delivering the intervention, but it is assumed that it was not possible due to the nature of the intervention (i.e., participants see what they do). There is no information about deviations from intended interventions or of moving participants after allocation.	Low risk of bias	Comment: There were no dropouts at the end of treatment and all participants were included in the data analysis.	High risk of bias	Comment: Used of standardised measurements and scales and timepoints of measurement were the same for the intervention and control group. No information about the blinding of outcome assessors was provided. Bias due to no blinding could have influenced the outcomes.	Some concerns	Comment: No protocol is reported, so it is not possible to assess if there was selection of reported outcomes based on the results or the analyses.	High risk of bias	Comment: There are some concerns or high risk of bias in most domains.
Manser 2023	Low risk of bias	Comment: Report of randomisation procedures and allocation concealment. No significant differences between groups at baseline.	Some concerns	Comment: Due to the nature of the intervention blinding was not possible. There is no information about deviations from intended interventions or of moving participants after allocation.	Low risk of bias	Comment: Dropout rates were above (20%) the threshold (5%) for the intervention group. A sensitivity analysis was performed and suggested similar results indicating that the findings were robust.	High risk of bias	Comment: Used standardised measurements and were the same for both intervention and control groups. Outcome assessors were not blinded.	Some concerns	Comment: A protocol was published retrospectively and based on the information provided in the clinical trial registration, it was not possible to assess if data was analysed accordingly.	High risk of bias	Comment: There are some concerns or high risk of bias in most domains.

**Table:** Risk of bias for analysis 3.3 Exergaming vs control at the end of treatment: change in activities of daily living (ADL)

**Study**	**Bias**											
	**Randomisation process**		**Deviations from intended interventions**		**Missing outcome data**		**Measurement of the outcome**		**Selection of the reported results**		**Overall**	
	**Authors' judgement**	**Support for judgement**	**Authors' judgement**	**Support for judgement**	**Authors' judgement**	**Support for judgement**	**Authors' judgement**	**Support for judgement**	**Authors' judgement**	**Support for judgement**	**Authors' judgement**	**Support for judgement**
Hughes 2014	Some concerns	Comment: Participants were randomly assigned to the intervention and control group using generated random numbers. Allocation concealment was not described. There were no baseline differences between groups.	Some concerns	Comment: No information about blinding of participants or carers/ those delivering the intervention, but it is assumed that it was not possible due to the nature of the intervention (i.e., participants see what they do). There is no information about deviations from intended interventions or of moving participants after allocation.	Low risk of bias	Comment: There were no dropouts at the end of treatment and all participants were included in the analyses.	High risk of bias	Comment: Used of standardised measurements and scales and timepoints of measurement were the same for the intervention and control group. No information about the blinding of outcome assessors was provided. Bias due to no blinding could have influenced the outcomes.	Some concerns	Comment: No protocol is reported, so it is not possible to assess if there was selection of reported outcomes based on the results or the analyses.	High risk of bias	There are some concerns or high risk of bias in most domains.
Lee 2021	Some concerns	Comment: Not enough information to assess the randomisation method. Allocation concealment was not described. No significant differences between groups at baseline.	Some concerns	Comment: No information about blinding of participants or carers/people delivering the intervention was provided, but due to the nature of the intervention we assume that blinding was not possible. There is no information about deviations from intended interventions or of moving participants after allocation.	Low risk of bias	Comment: There were no dropouts at the end of treatment and all participants were included in the data analysis.	High risk of bias	Comment: Used of standardised measurements and scales and timepoints of measurement were the same for the intervention and control group. No information about the blinding of outcome assessors was provided. Bias due to no blinding could have influenced the outcomes.	Some concerns	Comment: No protocol is reported, so it is not possible to assess if there was selection of reported outcomes based on the results or the analyses.	High risk of bias	Comment: There are some concerns or high risk of bias in most domains.

**Table:** Risk of bias for analysis 8.1 Exergaming vs alternative treatment for people with dementia

**Study**	**Bias**											
	**Randomisation process**		**Deviations from intended interventions**		**Missing outcome data**		**Measurement of the outcome**		**Selection of the reported results**		**Overall**	
	**Authors' judgement**	**Support for judgement**	**Authors' judgement**	**Support for judgement**	**Authors' judgement**	**Support for judgement**	**Authors' judgement**	**Support for judgement**	**Authors' judgement**	**Support for judgement**	**Authors' judgement**	**Support for judgement**
Karssemeijer 2019	Some concerns	There is evidence of random allocation but there is no information of allocation concealment. There is no information about significant baseline differences between the groups.	Some concerns	Comment: Blinding of participants was not possible due to the nature of the intervention and there is no information about blinding of carers or those delivering the intervention, but it is assumed the same limitations apply. There is no information about deviations from intended interventions or of moving participants after allocation.	Low risk of bias	Comment: Dropout was 13% in the control and 11% in the exergame condition, which is above the 5% threshold. A sensitivity analysis was performed and suggested similar results indicating that the findings were robust.	Low risk of bias	Used standardised measurements and were the same for both intervention and control groups. For adverse effects there is not enough information concerning the blining of outcome assessors, however, it is highly unlikely to impact the assessment of adverse effects.	Low risk of bias	Comment: Data were analysed according to the pre‐registered protocol. The outcomes and analyses were pre‐registered in the protocol.	Some concerns	Comment: The study is judged to have low risk of bias in most domains but there are some concerns for bias due to randomisation process and deviations from intended interventions.
Swinnen 2023	Some concerns	Comment: Report of randomisation procedures but no information about allocation concealment. There were no baseline differences between groups.	Some concerns	Comment: Participants and people delivering the interventions were not blinded. There is no information about deviations from intended interventions or of moving participants after allocation.	Low risk of bias	Comment: There is no report of sensitivity analysis or any other methods to correct for potential bias, but dropout rate and reasons were the same for the intervention and control groups.	Low risk of bias	Used standardised measurements and were the same for both intervention and control groups. For adverse effects there is not enough information concerning the blining of outcome assessors, however, it is highly unlikely to impact the assessment of adverse effects.	Low risk of bias	Comment: A protocol was published without enough information about the data analysis plan, but there is no indication for selecting specific outcomes based on the results or the analyses.	Some concerns	Comment: The study is judged to have low risk of bias in most domains but there are some concerns for bias due to randomisation process and deviations from intended interventions.

## Appendices

### Appendix 1. Sources searched and search strategies

**Table:**

**Source**	**Search strategy**	**Records retrieved**
1. CENTRAL (The Cochrane Library) https://crso.cochrane.org/SearchSimple.php Date of most recent search 2 March 2022	#1 MESH DESCRIPTOR Dementia EXPLODE ALL TREES 5849 #2 MESH DESCRIPTOR Delirium EXPLODE ALL TREES 765 #3 MESH DESCRIPTOR Wernicke Encephalopathy EXPLODE ALL TREES 4 #4 MESH DESCRIPTOR Neurocognitive Disorders EXPLODE ALL TREES 11042 #5 MESH DESCRIPTOR Cognition Disorders EXPLODE ALL TREES 5181 #6 (Benign senescent forgetfulness):TI,AB,KY 2 #7 (CDR 0.5):TI,AB,KY 0 #8 (clinical dementia rating scale 0.5):TI,AB,KY 0 #9 (cognit* impair*):TI,AB,KY 8691 #10 (GDS 3):TI,AB,KY 9 #11 (major neurocognitive disorder):TI,AB,KY 22 #12 (mild neurocognit* disorder*):TI,AB,KY 40 #13 (organic brain disease):TI,AB,KY 23 #14 (organic brain syndrome):TI,AB,KY 116 #15 (preclinical AD):TI,AB,KY 40 #16 (pre‐clinical AD):TI,AB,KY 2 #17 (preclinical alzheimer*):TI,AB,KY 47 #18 (pre‐clinical alzheimer*):TI,AB,KY 5 #19 (stage 3 GDS):TI,AB,KY 0 #20 ("global deterioration scale" and "stage 3"):TI,AB,KY 1 #21 ((cognit* or memory or cerebr* or mental*) adj3 (declin* or impair* or los* or deteriorat* or degenerat* or complain* or disturb* or disorder* or insufficient* or chronic)):TI,AB,KY 32009 #22 aMCI:TI,AB,KY 181 #23 MCIa:TI,AB,KY 0 #24 (lewy* adj2 bod*):TI,AB,KY 392 #25 (prodrom* adj2 dement*):TI,AB,KY 16 #26 alzheimer*:TI,AB,KY 11075 #27 dement*:TI,AB,KY 12787 #28 MCI:TI,AB,KY 2351 #29 #1 OR #2 OR #3 OR #4 OR #5 OR #6 OR #7 OR #8 OR #9 OR #10 OR #11 OR #12 OR #13 OR #14 OR #15 OR #16 OR #17 OR #18 OR #19 OR #20 OR #21 OR #22 OR #23 OR #24 OR #25 OR #26 OR #27 OR #28 47820 #30 MESH DESCRIPTOR Video Games EXPLODE ALL TREES 689 #31 MESH DESCRIPTOR Exercise Therapy EXPLODE ALL TREES WITH QUALIFIERS IS,MT 7581 #32 MESH DESCRIPTOR Virtual Reality Exposure Therapy EXPLODE ALL TREES 188 #33 MESH DESCRIPTOR Virtual Reality EXPLODE ALL TREES 236 #34 (active gaming):TI,AB,KY 12 #35 (active video gaming):TI,AB,KY 22 #36 (Bright Arm Duo):TI,AB,KY 0 #37 (Clinical Arcade):TI,AB,KY 0 #38 (cognitive‐aerobic bicycle training):TI,AB,KY 1 #39 (computer game*):TI,AB,KY 472 #40 (computer based):TI,AB,KY 2498 #41 (computer generated environment):TI,AB,KY 5 #42 (Dance Dance Revolution):TI,AB,KY 19 #43 (digital gam*):TI,AB,KY 33 #44 (exercise gaming):TI,AB,KY 7 #45 exer‐gam*:TI,AB,KY 4 #46 (gaming console*):TI,AB,KY 15 #47 (infra red camera*):TI,AB,KY 4 #48 (interactive gaming):TI,AB,KY 9 #49 (motion tracking):TI,AB,KY 84 #50 (play station):TI,AB,KY 3 #51 (Sony EyeToy):TI,AB,KY 0 #52 (video based):TI,AB,KY 690 #53 (video gam*):TI,AB,KY 1745 #54 (virtual city):TI,AB,KY 4 #55 (virtual environment):TI,AB,KY 313 #56 (virtual reality):TI,AB,KY 3513 #57 exergam*:TI,AB,KY 389 #58 gaming:TI,AB,KY 643 #59 IREX:TI,AB,KY 11 #60 Kinect*:TI,AB,KY 333 #61 Nintendo:TI,AB,KY 378 #62 playstation:TI,AB,KY 23 #63 videogam*:TI,AB,KY 254 #64 Wii:TI,AB,KY 568 #65 Xbox:TI,AB,KY 153 #66 (X box):TI,AB,KY 26 #67 (computer interaction):TI,AB,KY 142 #68 (exercise bike):TI,AB,KY 54 #69 (sports game*):TI,AB,KY 18 #70 gamecycle:TI,AB,KY 3 #71 immersive:TI,AB,KY 540 #72 (serious game*):TI,AB,KY 216 #73 treadmill:TI,AB,KY 8102 #74 #30 OR #31 OR #32 OR #33 OR #34 OR #35 OR #36 OR #37 OR #38 OR #39 OR #40 OR #41 OR #42 OR #43 OR #44 OR #45 OR #46 OR #47 OR #48 OR #49 OR #50 OR #51 OR #52 OR #53 OR #54 OR #55 OR #56 OR #57 OR #58 OR #59 OR #60 OR #61 OR #62 OR #63 OR #64 OR #65 OR #66 OR #67 OR #68 OR #69 OR #70 OR #71 OR #72 OR #73 24517 #75 #29 AND #74 1217	Jan 2021: 1217 March 2022: 185
2. MEDLINE In‐process and other non‐indexed citations and MEDLINE 1950‐present (Ovid SP) Date of most recent search 2 March 2022	1 exp Dementia/ 2 Delirium/ 3 Wernicke Encephalopathy/ 4 Neurocognitive Disorders/ 5 exp *Cognition Disorders/ 6 "Benign senescent forgetfulness".ti,ab. 7 "CDR 0.5".ti,ab. 8 "clinical dementia rating scale 0.5".ti,ab. 9 "cognit* impair*".ti,ab. 10 "GDS 3".ti,ab. 11 "major neurocognitive disorder".ti,ab. 12 "mild neurocognit* disorder*".ti,ab. 13 "organic brain disease".ti,ab. 14 "organic brain syndrome".ti,ab. 15 "preclinical AD".ti,ab. 16 "pre‐clinical AD".ti,ab. 17 "preclinical alzheimer*".ti,ab. 18 "pre‐clinical alzheimer*".ti,ab. 19 "stage 3 GDS".ti,ab. 20 ("global deterioration scale" and "stage 3").ti,ab. 21 ((cognit* or memory or cerebr* or mental*) adj3 (declin* or impair* or los* or deteriorat* or degenerat* or complain* or disturb* or disorder* or insufficient* or chronic)).ti,ab. 22 aMCI.ti,ab. 23 MCIa.ti,ab. 24 (lewy* adj2 bod*).ti,ab. 25 (prodrom* adj2 dement*).ti,ab. 26 alzheimer*.ti,ab. 27 dement*.ti,ab. 28 MCI.ti,ab. 29 or/1‐28 30 exp Video Games/ 31 exp Exercise Therapy/mt [Methods] 32 Virtual Reality Exposure Therapy/ 33 Virtual Reality/ 34 exp Exercise Therapy/is [Instrumentation] 35 "active gaming".ti,ab. 36 "active video gaming".ti,ab. 37 "Bright Arm Duo".ti,ab. 38 "Clinical Arcade".ti,ab. 39 "cognitive‐aerobic bicycle training".ti,ab. 40 "computer game*".ti,ab. 41 "computer based".ti,ab. 42 "computer generated environment".ti,ab. 43 "Dance Dance Revolution".ti,ab. 44 "digital gam*".ti,ab. 45 "exercise gaming".ti,ab. 46 "exer‐gam*".ti,ab. 47 "gaming console*".ti,ab. 48 "infra red camera*".ti,ab. 49 "interactive gaming".ti,ab. 50 "motion tracking".ti,ab. 51 "play station".ti,ab. 52 "Sony EyeToy".ti,ab. 53 "video based".ti,ab. 54 "video gam*".ti,ab. 55 "virtual city".ti,ab. 56 "virtual environment".ti,ab. 57 "virtual reality".ti,ab. 58 exergam*.ti,ab. 59 gaming.ti,ab. 60 IREX.ti,ab. 61 Kinect*.ti,ab. 62 Nintendo.ti,ab. 63 playstation.ti,ab. 64 videogam*.ti,ab. 65 Wii.ti,ab. 66 Xbox.ti,ab. 67 "X box".ti,ab. 68 "computer interaction".ti,ab. 69 "exercise bike".ti,ab. 70 "sports game*".ti,ab. 71 gamecycle.ti,ab. 72 immersive.ti,ab. 73 "serious game*".ti,ab. 74 treadmill.ti,ab. 75 or/30‐74 76 29 and 75 77 randomized controlled trial.pt. 78 controlled clinical trial.pt. 79 randomized.ab. 80 placebo.ab. 81 drug therapy.fs. 82 randomly.ab. 83 trial.ab. 84 groups.ab. 85 or/77‐84 86 exp animals/ not humans.sh. 87 85 not 86 88 76 and 87	Jan 2021: 1211 March 2022: 180
3. EMBASE (Ovid SP) 1974 to present Date of most recent search 2 March 2022	1 dementia/ 2 delirium/ 3 Wernicke encephalopathy/ 4 mild cognitive impairment/ 5 dement*.ti,ab. 6 alzheimer*.ti,ab. 7 (lewy* adj2 bod*).ti,ab. 8 (chronic adj2 cerebrovascular).ti,ab. 9 "organic brain disease".ti,ab. 10 "organic brain syndrome".ti,ab. 11 "benign senescent forgetfulness".ti,ab. 12 (cerebr* adj2 deteriorat*).ti,ab. 13 (cerebral* adj2 insufficient*).ti,ab. 14 "major neurocognitive disorder*".ti,ab. 15 "mild neurocognitive disorder*".ti,ab. 16 aMCI.ti,ab. 17 MCI.ti,ab. 18 "mild cognitive impairment".ti,ab. 19 ((cognit* or memory or cerebr* or mental*) adj3 (declin* or impair* or los* or deteriorat* or degenerat* or complain* or disturb* or disorder* or insufficient* or chronic)).ti,ab. 20 or/1‐19 21 exp video game/ 22 exp kinesiotherapy/ 23 virtual reality exposure therapy/ 24 exp virtual reality/ 25 "active gaming".ti,ab. 26 "active video gaming".ti,ab. 27 "Bright Arm Duo".ti,ab. 28 "Clinical Arcade".ti,ab. 29 "cognitive‐aerobic bicycle training".ti,ab. 30 "computer game*".ti,ab. 31 "computer based".ti,ab. 32 "computer generated environment".ti,ab. 33 "Dance Dance Revolution".ti,ab. 34 "digital gam*".ti,ab. 35 "exercise gaming".ti,ab. 36 "exer‐gam*".ti,ab. 37 "gaming console*".ti,ab. 38 "infra red camera*".ti,ab. 39 "interactive gaming".ti,ab. 40 "motion tracking".ti,ab. 41 "play station".ti,ab. 42 "Sony EyeToy".ti,ab. 43 "video based".ti,ab. 44 "video gam*".ti,ab. 45 "virtual city".ti,ab. 46 "virtual environment".ti,ab. 47 "virtual reality".ti,ab. 48 exergam*.ti,ab. 49 gaming.ti,ab. 50 IREX.ti,ab. 51 Kinect*.ti,ab. 52 Nintendo.ti,ab. 53 playstation.ti,ab. 54 videogam*.ti,ab. 55 Wii.ti,ab. 56 Xbox.ti,ab. 57 "X box".ti,ab. 58 "computer interaction".ti,ab. 59 "exercise bike".ti,ab. 60 "sports game*".ti,ab. 61 gamecycle.ti,ab. 62 immersive.ti,ab. 63 "serious game*".ti,ab. 64 treadmill.ti,ab. 65 or/21‐64 66 20 and 65 67 randomized controlled trial/ 68 controlled clinical trial/ 69 random$.ti,ab. 70 randomization/ 71 intermethod comparison/ 72 placebo.ti,ab. 73 (compare or compared or comparison).ti. 74 ((evaluated or evaluate or evaluating or assessed or assess) and (compare or compared or comparing or comparison)).ab. 75 (open adj label).ti,ab. 76 ((double or single or doubly or singly) adj (blind or blinded or blindly)).ti,ab. 77 double blind procedure/ 78 parallel group$1.ti,ab. 79 (crossover or cross over).ti,ab. 80 ((assign$ or match or matched or allocation) adj5 (alternate or group$1 or intervention$1 or patient$1 or subject$1 or participant$1)).ti,ab. 81 (assigned or allocated).ti,ab. 82 (controlled adj7 (study or design or trial)).ti,ab. 83 (volunteer or volunteers).ti,ab. 84 trial.ti. 85 or/67‐84 86 66 and 85	Jan 2021: 1986 March 2022: 400
4. PSYCINFO (Ovid SP) Date of most recent search 2 March 2022	1 exp Dementia/ 2 exp Delirium/ 3 exp Wernicke's Syndrome/ 4 exp Cognitive Impairment/ 5 dement*.mp. 6 alzheimer*.mp. 7 deliri*.mp. 8 (lewy* adj2 bod*).mp. 9 (chronic adj2 cerebrovascular).mp. 10 "organic brain disease".mp. 11 "organic brain syndrome".mp. 12 "benign senescent forgetfulness".mp. 13 (cerebr* adj2 deteriorat*).mp. 14 (cerebral* adj2 insufficient*).mp. 15 "major neurocognitive disorder*".ti,ab. 16 "mild neurocognitive disorder*".ti,ab. 17 aMCI.ti,ab. 18 MCI.ti,ab. 19 "mild cognitive impairment".ti,ab. 20 or/1‐19 21 exp Computer Games/ 22 exp Virtual Reality Exposure Therapy/ 23 exp Virtual Reality/ 24 "active gaming".ti,ab. 25 "active video gaming".ti,ab. 26 "Bright Arm Duo".ti,ab. 27 "Clinical Arcade".ti,ab. 28 "cognitive‐aerobic bicycle training".ti,ab. 29 "computer game*".ti,ab. 30 "computer based".ti,ab. 31 "computer generated environment".ti,ab. 32 "Dance Dance Revolution".ti,ab. 33 "digital gam*".ti,ab. 34 "exercise gaming".ti,ab. 35 "exer‐gam*".ti,ab. 36 "gaming console*".ti,ab. 37 "infra red camera*".ti,ab. 38 "interactive gaming".ti,ab. 39 "motion tracking".ti,ab. 40 "play station".ti,ab. 41 "Sony EyeToy".ti,ab. 42 "video based".ti,ab. 43 "video gam*".ti,ab. 44 "virtual city".ti,ab. 45 "virtual environment".ti,ab. 46 "virtual reality".ti,ab. 47 exergam*.ti,ab. 48 gaming.ti,ab. 49 IREX.ti,ab. 50 Kinect*.ti,ab. 51 Nintendo.ti,ab. 52 playstation.ti,ab. 53 videogam*.ti,ab. 54 Wii.ti,ab. 55 Xbox.ti,ab. 56 "X box".ti,ab. 57 "computer interaction".ti,ab. 58 "exercise bike".ti,ab. 59 "sports game*".ti,ab. 60 gamecycle.ti,ab. 61 immersive.ti,ab. 62 "serious game*".ti,ab. 63 treadmill.ti,ab. 64 or/21‐63 65 20 and 64 66 exp Clinical Trials/ 67 randomly.ab. 68 randomi?ed.ti,ab. 69 placebo.ti,ab. 70 groups.ab. 71 "double‐blind*".ti,ab. 72 "single‐blind*".ti,ab. 73 RCT.ti,ab. 74 or/66‐73 75 65 and 74	Jan 2021: 211 March 2022: 23
5. CINAHL (EBSCOhost) Date of most recent search 2 March 2022	S88 S73 AND S86 S87 S73 AND S86 S86 S74 OR S75 OR S76 OR S77 OR S78 OR S79 OR S80 OR S81 OR S82 OR S83 OR S84 OR S85 S85 MH "Random Assignment" S84 MH "Single‐Blind Studies" or MH "Double‐Blind Studies" or MH "Triple‐Blind Studies" S83 MH "Crossover Design" S82 MH "Factorial Design" S81 MH "Placebos" S80 MH "Clinical Trials" S79 TX "multi‐centre study" OR "multi‐center study" OR "multicentre study" OR "multicenter study" OR "multi‐site study" S78 TX crossover OR "cross‐over" S77 AB placebo* S76 TX random* S75 TX trial* S74 TX "latin square" S73 S27 AND S72 S72 S28 OR S29 OR S30 OR S31 OR S32 OR S33 OR S34 OR S35 OR S36 OR S37 OR S38 OR S39 OR S40 OR S41 OR S42 OR S43 OR S44 OR S45 OR S46 OR S47 OR S48 OR S49 OR S50 OR S51 OR S52 OR S53 OR S54 OR S55 OR S56 OR S57 OR S58 OR S59 OR S60 OR S61 OR S62 OR S63 OR S64 OR S65 OR S66 OR S67 OR S68 OR S69 OR S70 OR S71 S71 TX treadmill S70 TX "serious game*" S69 TX immersive S68 TX gamecycle S67 TX "sports game*" S66 TX "exercise bike" S65 TX "computer interaction" S64 TX "X box" S63 TX Xbox S62 TX Wii S61 TX videogam* S60 TX playstation S59 TX Nintendo S58 TX Kinect* S57 TX IREX S56 TX gaming S55 TX exergam* S54 TX "virtual reality" S53 TX "virtual environment" S52 TX "virtual city" S51 TX "video gam*" S50 TX "video based" S49 TX "Sony EyeToy" S48 TX "play station" S47 TX "motion tracking" S46 TX "interactive gaming" S45 TX "infra red camera*" S44 TX "gaming console*" S43 TX "exer‐gam*" S42 TX "exercise gaming" S41 TX "digital gam*" S40 TX "Dance Dance Revolution" S39 TX "computer generated environment" S38 TX "computer based" S37 TX "computer game*" S36 TX "cognitive‐aerobic bicycle training" S35 TX "Clinical Arcade" S34 TX "Bright Arm Duo" S33 TX "active video gaming" S32 TX "active gaming" S31 (MH "Virtual Reality+") S30 (MH "Virtual Reality Exposure Therapy") S29 (MH "Therapeutic Exercise+/MT") S28 (MH "Video Games+") S27 S1 OR S2 OR S3 OR S4 OR S5 OR S6 OR S7 OR S8 OR S9 OR S10 OR S11 OR S12 OR S13 OR S14 OR S15 OR S16 OR S17 OR S18 OR S19 OR S20 OR S21 OR S22 OR S23 OR S24 OR S25 OR S26 S26 TX ((cognit* or memory or cerebr* or mental*) N3 (declin* or impair* or los* or deteriorat* or degenerat* or complain* or disturb* or disorder* or insufficient* or chronic)) S25 TX MCI S24 TX aMCI S23 TX "mild cognitive impairment" S22 TX "mild neurocognitive disorder*" S21 TX "major neurocognitive disorder" S20 TX korsako* S19 TX binswanger* S18 TX huntington* S17 TX creutzfeldt or jcd or cjd S16 TX pick* N2 disease S15 TX cerebral* N2 insufficient* S14 TX cerebr* N2 deteriorat* S13 TX "benign senescent forgetfulness" S12 TX "normal pressure hydrocephalus" and "shunt*" S11 TX "organic brain disease" or "organic brain syndrome" S10 TX chronic N2 cerebrovascular S9 TX deliri* S8 TX lewy* N2 bod* S7 TX alzheimer* S6 TX dement* S5 (MH "Mild Cognitive Impairment") S4 MH "Wernicke's Encephalopathy" S3 MH "Delirium, Dementia, Amnestic, Cognitive Disorders" S2 MH "Delirium" S1 MH "Dementia+"	Jan 2021: 653 March 2022: 69
6. Web of Science – core collection (Clarivate) Date of most recent search 2 March 2022	TOPIC: (dement* OR alzheimer* OR "vascular cognitive impairment" OR "lew* bod*" OR CADASIL OR "cognit* impair*") AND TOPIC: (Exergam* OR "Video Gam*" OR "Virtual Reality" OR "computer gam*" OR gaming) AND TOPIC: (randomly OR randomised OR randomized OR "random allocat*" OR RCT OR CCT OR "double blind*" OR "single blind*" OR "double blind*" OR "single blind*" OR trial)	Jan 2021: 300 March 2022: 129
7. LILACS (BIREME) Date of most recent search 2 March 2022	alzheimer OR alzheimers OR alzheimer’s OR dementia OR demenc$ OR neurocognitive disorder [Words] and randomly OR randomised OR randomized OR RCT OR controlled trial OR double blind$ OR placebo [Words] and Exergame OR Video Game OR Virtual Reality OR computer game OR gaming [Words]	Jan 2021: 0 March 2022: 0
8. ClinicalTrials.gov (www.clinicaltrials.gov) Date of most recent search 2 March 2022	dementia OR alzheimers OR cognition OR cognitive | Exergame OR "Video Game" OR "Virtual Reality" OR "computer game" OR gaming	Jan 2021: 268 March 2022: 78
9. ICTRP Date of most recent search 2 March 2022	dementia OR alzheimers OR cognition OR cognitive | Exergame OR "Video Game" OR "Virtual Reality" OR "computer game" OR gaming	Jan 2021: 0 March 2022: 60
10. CDCIG specialised register (CRS web) Date of most recent search 2 March 2022	#1 Video Games AND INREGISTER #2 Virtual Reality AND INREGISTER #3 Exergam* AND INREGISTER #4 active video gaming AND INREGISTER #5 computer gam* AND INREGISTER #6 computer based AND INREGISTER #7 digital gam* AND INREGISTER #8 gaming AND INREGISTER #9 virtual environment AND INREGISTER #10 Nintendo AND INREGISTER #11 videogam* AND INREGISTER #12 Wii AND INREGISTER #13 Xbox AND INREGISTER #14 X box AND INREGISTER #15 serious game* AND INREGISTER #16 #1 OR #2 OR #3 OR #4 OR #5 OR #6 OR #7 OR #8 OR #9 OR #10 OR #11 OR #12 OR #13 OR #14 OR #15	Jan 2021: 293 March 2022: 96
TOTAL before de‐duplication		Jan 2021: 6139 March 2022: 1220
Total after de‐duplication		Jan 2021: 3911 March 2022: 859

### Appendix 2. Planned subgroup analysis

For each outcome, we had planned subgroup analyses if heterogeneity (I²) was higher than 40%, and there were at least three studies per subgroup. It was not possible to conduct subgroup analyses (see Differences between protocol and review), but in future updates, we will consider the following subgroups.

- Severity of dementia: mild versus moderate versus severe
- Intervention characteristics: physical activity with or without the addition of specific cognitive training or rehabilitative tasks (beyond the basic performance of the game)
- Type of control intervention (for the comparisons of exergaming versus control): inactive control (no intervention or treatment as usual) versus active control (intervention involving equivalent contact with the researchers but not hypothesised to have any specific effect on the study outcomes)
- Type of exergame platform used: commercial versus customised
- Type of technology: VR‐based (e.g. head mounted display headsets such as Oculus Rift) versus monitor display (e.g. Wii Fit, Sports)
- Length of intervention: the total time of the intervention in minutes: 0 to 360 minutes (6 hours) versus 361 to 720 minutes (12 hours) versus more than 720 minutes
- Length of follow‐up period: 0 to 1 month versus 1 to 3 months versus 4 to 6 months versus longer than 6 months

### Appendix 3. Planned sensitivity analysis

We did not conduct any sensitivity analyses (see Differences between protocol and review). In future updates, to assess the robustness of our findings, we may perform the following sensitivity analyses for our primary outcomes by removing:

- results from studies at high risk of bias (high risk in at least two domains) or with some concerns;
- results from studies that are not dose‐matched (i.e. intervention and control interventions had unequal doses in terms of time or frequency);
- results from studies that do not use strict criteria to define MCI (e.g. criteria proposed by Petersen 2009);
- results from studies with low statistical power. We define powered studies as those that have a post hoc achieved power larger than 0.80 (calculated using GPower software (Faul 2007)).

## Data and analyses

**Comparison 1:** Exergaming vs control at the end of treatment for people with dementia

Outcome or subgroup title	No. of studies	No. of participants	Statistical method	Effect size
1.1 Exergaming vs control at the end of treatment: change in global physical functioning (composite)	2	113	Std. Mean Difference (IV, Fixed, 95% CI)	‐0.20 [‐0.57, 0.17]
1.2 Exergaming vs control at the end of treatment: change in global cognitive functioning (composite)	2	113	Std. Mean Difference (IV, Fixed, 95% CI)	1.47 [1.04, 1.90]
1.3 Exergaming vs control at the end of treatment: change in activities for daily living (ADL)	2	113	Std. Mean Difference (IV, Fixed, 95% CI)	‐0.28 [‐0.65, 0.09]

**Comparison 2:** Exergaming vs alternative treatment at the end of treatment for people with dementia

Outcome or subgroup title	No. of studies	No. of participants	Statistical method	Effect size
2.1 Exergaming vs alternative treatment at the end of treatment: change in global physical functioning (composite)	2	85	Std. Mean Difference (IV, Fixed, 95% CI)	0.14 [‐0.30, 0.58]
2.2 Exergaming vs alternative treatment at the end of treatment: change in functional status and physical performance	2	85	Mean Difference (IV, Fixed, 95% CI)	0.80 [‐0.57, 2.18]
2.3 Exergaming vs alternative treatment at the end of treatment: change in global cognitive functioning (composite)	2	85	Std. Mean Difference (IV, Fixed, 95% CI)	0.11 [‐0.33, 0.55]
2.4 Exergaming vs alternative treatment at the end of treatment: change in lower limb functioning	2		Std. Mean Difference (IV, Fixed, 95% CI)	Subtotals only

**Comparison 3:** Exergaming vs control at the end of treatment for people with MCI

Outcome or subgroup title	No. of studies	No. of participants	Statistical method	Effect size
3.1 Exergaming vs control at the end of treatment: change in global physical functioning (composite)	2	34	Std. Mean Difference (IV, Fixed, 95% CI)	0.27 [‐0.41, 0.94]
3.2 Exergaming vs control at the end of treatment: change in global cognitive functioning (composite)	2	34	Std. Mean Difference (IV, Fixed, 95% CI)	0.79 [0.05, 1.53]
3.3 Exergaming vs control at the end of treatment: change in activities of daily living (ADL)	2	60	Std. Mean Difference (IV, Fixed, 95% CI)	0.51 [‐0.01, 1.03]
3.4 Exergaming vs control at the end of treatment: change in lower limb functioning	2	34	Std. Mean Difference (IV, Fixed, 95% CI)	0.27 [‐0.41, 0.94]
3.5 Exergaming vs control at the end of treatment: change in general cognition	2	34	Std. Mean Difference (IV, Fixed, 95% CI)	0.46 [‐0.22, 1.15]
3.6 Exergaming vs control at the end of treatment: change in attention, processing speed, working memory	2	34	Std. Mean Difference (IV, Fixed, 95% CI)	1.14 [‐0.03, 2.31]

**Comparison 7:** Exergaming vs control at follow‐up for people with MCI

Outcome or subgroup title	No. of studies	No. of participants	Statistical method	Effect size
7.1 Exergaming vs control at follow‐up: change in global cognitive functioning (composite)	2	38	Std. Mean Difference (IV, Fixed, 95% CI)	0.02 [‐0.62, 0.67]
7.2 Exergaming vs control at follow‐up: change in general cognition	2	38	Std. Mean Difference (IV, Fixed, 95% CI)	0.01 [‐0.64, 0.65]

**Comparison 8:** Adverse effects

Outcome or subgroup title	No. of studies	No. of participants	Statistical method	Effect size
8.1 Exergaming vs alternative treatment for people with dementia	2		Risk Ratio (M‐H, Fixed, 95% CI)	Subtotals only

**Comparison 9:** Feasibility and treatment adherence

Outcome or subgroup title	No. of studies	No. of participants	Statistical method	Effect size
9.1 Number of dropouts at the end of treatment (exergaming vs control intervention) for people with dementia	2	132	Risk Ratio (M‐H, Fixed, 95% CI)	0.89 [0.39, 2.05]
9.2 Number of dropouts at the end of treatment (exergaming vs alternative treatment) for people with dementia	2	99	Risk Ratio (M‐H, Fixed, 95% CI)	0.88 [0.33, 2.32]
9.3 Number of dropouts at the end of treatment (exergaming vs control intervention) for people with mild cognitive impairment (MCI)	3	76	Risk Ratio (M‐H, Fixed, 95% CI)	3.18 [0.18, 56.95]
9.4 Number of dropouts at the end of treatment (exergaming vs alternative treatment) for people with mild cognitive impairment (MCI)	4	243	Risk Ratio (M‐H, Fixed, 95% CI)	1.50 [0.45, 4.99]
9.5 Number of dropouts at follow‐up (exergame vs control intervention) for people with dementia	2	144	Risk Ratio (M‐H, Fixed, 95% CI)	0.99 [0.48, 2.04]
9.6 Number of dropouts at follow‐up (exergame vs control intervention) for people with mild cognitive impairment (MCI)	2	43	Risk Ratio (M‐H, Fixed, 95% CI)	0.24 [0.03, 1.93]

## Characteristics of studies

### Characteristics of included studies [ordered by study ID]

**Table:** Arshad 2023

***Study characteristics***	
Methods	RCT with 2 groups: exergaming and physical exercise
Participants	56 participants with MCI were randomised; 51 completed the study. MCI diagnosis based on NIA‐AA and Petersen criteria
Interventions	In the exergame intervention, participants (N = 26) played a commercially available videogame, published by Namco Bandai designed for virtual reality‐based Xbox Kinect 360, Body, and Brain Exercises by Dr Kawashima. In total, 9 games were used: “Time abomb” and “Match Maker” in the logic category; “Off the Radar” and “Flip and Find” in the memory category; “Traffic Control” and “Mouse Mayhem” in the physical category; “Balloon Buster” and “Flag Frenzy” in the reflex category; and “Perfect 10”. For all games, participants were required to control the games with body movements. In the alternative treatment condition, participants (N = 25) performed stretching of all major muscle groups of upper extremity and lower extremity, and strength training with use of elastic bands. Participants met for 30 minutes, 5 times per week for 6 weeks for a total of 15 hours.
Outcomes	Outcomes were recorded at baseline and end of treatment (6 weeks). Outcomes included cognitive functioning outcomes (e.g. MMSE (Folstein 1975); MoCA (Nasreddine 2005); TMT A and B (Reitan 1955); VFT Semantic and Phonemic).
Notes	The study took place in Pakistan; no follow‐up assessments.

**Table:** Hughes 2014

***Study characteristics***	
Methods	RCT with 2 groups: exergaming and health education
Participants	20 participants with MCI were randomised; there were no dropouts at the end of treatment and 1 at follow‐up. MCI diagnosis based on conventional MCI criteria and MYHAT criteria (Ganguli 2010).
Interventions	The exergame intervention (N = 10) consisted of interactive videogames (Nintendo Wii). Various videogames (e.g. Wii Sports such as bowling, golf, tennis, and baseball) were played where participants used their limbs or body to simulate actions required to play the game. Starting with week 7, new sports were introduced (e.g. Wii play and Sports Resort). In weeks 10 and 20, participants were enroled into Wii tournament. The active control intervention (N = 10) consisted of a healthy ageing education programme as a form of passive cognitive stimulation in which participants had to learn about and discuss age‐specific health‐related topics with professionals (e.g. safe driving, medication management, caregiving, healthy cooking, sleep hygiene). To offer similar motivation to the exergame group, participants at the end of each session took part into small group activities and in weeks 10 and 20 in 'Jeopardy' style tournament. Participants met for 90 min once per week for 24 weeks for a total of 36 hours.
Outcomes	Outcomes were recorded at baseline, end of treatment (24 weeks), and follow‐up (12 months). Outcomes included physical functioning (gait speed measured with a 6 m Walk Test); cognitive functioning (e.g. CAMCI and two tracking tasks (Saxton 2009)); ADL (TIADL, Owsley 2002).
Notes	Feasibility study; the study took place in the USA.

**Table:** Karssemeijer 2019

***Study characteristics***	
Methods	RCT with 1 experimental group (exergaming) and 2 control groups (alternative treatment ‐ aerobic training; active control ‐ relaxation and flexibility exercises)
Participants	115 participants with dementia were randomised; 101 participants completed the assessments at the end of treatment and 92 at follow‐up. Dementia diagnosis was made based on DSM‐IV criteria (vascular, Alzheimer's, or mixed subtype).
Interventions	Exergame intervention (N = 34) consisted of a combined cognitive–aerobic bicycle training developed by Bike Labyrinth (www.bikelabyrinth.com). Participants had to perform physical exercises (cycling on a stationary bike by following a route in VR) and simultaneously had to solve cognitive tasks (e.g. response inhibition). In the alternative treatment group, aerobic training (N = 33), participants were asked to cycle on a stationary bike that was not connected to a screen. In the active control group (N = 34), participants performed relaxation and flexibility exercises with similar levels of engagement as other groups. Participants in each group received 3 sessions per week for 12 weeks for approximately 30 minutes. Training sessions were administered individually.
Outcomes	Full assessments were recorded at baseline, end of treatment (12 weeks) and at follow‐up (24 weeks). Intermediate measurements were performed after 6 weeks of training. Outcomes consisted of physical functioning (e.g. TUG (Podsiadlo 1991; 10 m Walk Test (Wade 1992); SST (Bohannon 1995); FICSIT‐4 (Ory 1993)); cognitive functioning (e.g. WAIS‐III Digit Span (Wechsler 1997), and WMS‐III Spatial Span (Wechsler 1997b); Stroop Test (Jensen 1966); TMT (Reitan 1955); Letter Fluency (Schmand 2008); LLT (Bucks 2011); RSCT (Wilson 1997)); ADL (Katz index, Katz 1963); functional status (SPPBT, Guralnik 1994); frailty (EFIP, De Vries 2013).
Notes	We did not use the intermediate assessments in this review; the study was conducted in the Netherlands.

**Table:** Lee 2021

***Study characteristics***	
Methods	RCT with 2 groups: exergame intervention and usual care
Participants	40 geriatric participants with an MCI diagnosis based on the Petersen criteria. There were no dropouts at the end of treatment.
Interventions	Exergame intervention (N = 20) consisted of playing Wii Sports Resort games. Four sports (fencing, bowling, table tennis, and frisbee) were selected in line with the education level and the cultural characteristics of the participants. Participants received 3 sessions of 15 minutes per week for 12 weeks. The intervention comprised 36 sessions in total. The inactive control group continued their conventional elderly care (N = 20).
Outcomes	Measurements took place at baseline and end of treatment (12 weeks) with ADL (S‐IADL, Kang 2002) as main outcome.
Notes	The study took place in the Republic of Korea; no follow‐up assessments.

**Table:** Manser 2023

***Study characteristics***	
Methods	Pilot RCT with 12 groups: exergaming and usual care
Participants	16 participants with mNCD were randomised; 14 completed the study. The diagnosis was based on DSM 5, ICD 11, or adapted Petersen criteria.
Interventions	The exergame group (N = 8) trained at home using the “Senso Flex”, a home‐based version of the “Senso” platform (Dividad software). The training is based on Brain IT concept, a multi‐domain exergame‐based simultaneous motor–cognitive training with incorporated cognitive tasks combined with HRV‐guided resonance breathing. The inactive control group (N = 6) continued with usual care as provided by the (memory) clinics where participants were recruited. The exergame group trained for about 21 minutes 5 times a week for 12 weeks.
Outcomes	Outcomes were recorded at baseline and up to 2 weeks after the end of treatment (14 weeks) and included physical functioning (e.g. walk speed, stride duration and length (Bioengineering 2017)); cognitive functioning measures (e.g. QMCI (O’Caoimh 2017); WMS Logical Memory (Wechsler 2009); DS Forward and Backward (Croschere 2012; Mueller 2014); TAP alertness, Go‐NoGo, incompatibility (Zimmermann 2012); TMT A and B (Mueller 2014); HOTAP picture‐sorting test (Menzel‐Begemann 2009); mental rotation task (Mueller 2014)); and quality of life in Alzheimer's disease (QoL‐AD, Logsdon 1999).
Notes	Pilot study; primary outcomes were usability and feasibility; physical and cognitive functioning were secondary outcomes; the study was conducted in Switzerland; no follow‐up assessments.

**Table:** Park 2018

***Study characteristics***	
Methods	RCT with 2 groups: exergaming and computerised cognitive training
Participants	78 older people with amnestic MCI were randomised. There were no dropouts at the end of treatment. MCI diagnosis followed Petersen criteria.
Interventions	The exergaming group (N = 39) performed non‐specific computer training (NCT) consisting of games played via the Wii platform. Three games from Wii Sports Resorts were played (table tennis, sword play, and archery). Each game was played for 10 minutes. In the alternative treatment group (N = 39), participants completed the CoTras programme, a cognition‐specific computer training (CCT). This consisted of various training programmes for cognitive functions such as attention, memory, and visual spatial abilities, each being trained for 10 minutes during one session. Participants in each group performed a total of 30 sessions, 3 days per week for 10 weeks.
Outcomes	Outcomes were recorded at baseline and end of treatment (10 weeks). They consisted of cognitive functioning measures (e.g. Trail Making Test A and B (Reitan 1955); Digit Span and Block Design Test‐WAIS (Wechsler 2008); RAVLT and R‐OCFT (Strauss 2006); Stroop Test (Stroop 1935)); and health‐related quality of life (SF‐36 (Mchorney 1993)).
Notes	The study was conducted in the Republic of Korea; no follow‐up assessments.

**Table:** Ramnath 2021

***Study characteristics***	
Methods	Cluster‐RCT with 2 groups: exergaming and conventional multimodal exercise
Participants	45 participants diagnosed with MCI using adapted Petersen criteria. There were no dropouts at the end of treatment.
Interventions	The exergame group underwent interactive video gaming (N = 23) delivered using the X‐Box Kinect Sports videogaming software and consisted of six games: 10‐pin bowling, boxing, track and field, table tennis, beach volleyball, and soccer. Participants played the session in dyads and two pairs played during one session (30 minutes per dyad for physical exercise and 30 minutes resting; 15 minutes each). The alternative treatment group received conventional multimodal training (N = 22) and participated in group sessions of a low‐intensity combination of standing and seated exercises. Each session consisted of 10 minutes of warm up, 30 minutes of strength training, 10 minutes of proprioceptive exercises, and 10 minutes of cooling down. All sessions were supervised and lasted for 1 hour, 2 days per week for 12 weeks.
Outcomes	Outcomes were recorded at baseline and end of treatment (12 weeks). Outcomes included measures of physical functioning (e.g. 6‐Minute Walk Test, Guyatt 1985; TUG, Bischoff 2003; 10 m Test, Winograd 1994; FRT, Weiner 1992)) and cognitive functioning (e.g. MMSE (Folstein 1975); N‐back Task (Owen 2005); and the Stroop task (Stroop 1935)).
Notes	Data were analysed at participant not cluster level; the study was conducted in South Africa; no follow‐up assessments.

**Table:** Robert 2021

***Study characteristics***	
Methods	Cluster‐RCT with 2 groups: exergaming and usual care
Participants	91 participants met the DSM 5 diagnostic criteria for mMCI and MNCD. 19 people with mNCD and 50 people with MNCD completed the assessments at follow‐up.
Interventions	The exergame intervention (N mNCD = 8; N MNCD = 20) was delivered via X‐Torp game developed by the Az@GAME project. X‐Torp stimulates cognitive capacities, promotes physical activity, and helps maintain social ties (http://www.innovation-alzheimer.fr/azgame-en/). During the game, participants have to control a submarine with their body movements, and to stimulate cognitive functions, they have to meet several objectives, including playing mini‐games and orientation exercises. The inactive control group (N mNCD = 11; N MNCD = 30) received their usual care. The intervention group underwent 2 sessions per week, for 12 weeks. Each session lasted 15 minutes. Sessions were organised in groups or individually.
Outcomes	Outcomes were recorded at baseline, end of treatment (12 weeks), and follow‐up (24 weeks). Outcomes recorded at the end of treatment were not of interest for this review (Neuropsychiatric Inventory (Cummings 1994); the Apathy Inventory, clinician’s version (Robert 2002)). The MMSE (Folstein 1975) was recorded only at 24 weeks as a measure of global cognitive functioning. Because of this, for this study, we could only use data for follow‐up assessments.
Notes	Data were analysed at participant level and not cluster level. The study was conducted in France. It included only follow‐up assessments.

**Table:** Swinnen 2021

***Study characteristics***	
Methods	RCT with 2 groups: exergaming and an active control group who listened to favourite music
Participants	55 participants with MNCD according to DSM 5 criteria were randomised. 45 completed the assessments at the end of treatment.
Interventions	The exergame group (N = 23) performed physical exercises using the device “Dividat Senso” (Dividat, Schindellegi, Switzerland). This device consisted of a step training platform (1.13 m × 1.13 m) that was sensitive to pressure changes (strain gauges measuring at 50 Hz) and participants played various games that were controlled by lower limb/step movements. The games trained the following cognitive abilities: divided and selective attention, flexibility, postural control, and visuospatial working memory. In the active control condition (N = 22), participants watched and listened to their favourite music displayed on a TV screen. Both groups trained for 8 weeks, 3 times per week with a duration of 15 minutes per session. Usual care was added to both the exergame and the control music intervention groups; this consisted of pharmacotherapy and physiotherapy focusing on comfort care.
Outcomes	Participants were assessed at baseline and end of treatment (8 weeks) for outcomes such as physical functioning (e.g. gait speed); cognitive functioning (e.g. MoCA (Nasreddine 2005)); ADL (e.g. Katz Index, Katz 1963); and functional status (SPPBT, Guralnik 1994).
Notes	Pilot study conducted in Belgium; no follow‐up assessments.

**Table:** Swinnen 2023

***Study characteristics***	
Methods	RCT with 2 groups: exergaming and exercise therapy
Participants	23 people with a diagnosis of MNCD according to DSM 5 criteria. All had either vascular dementia, Alzheimer’s disease, mixed dementia, Parkinson’s disease, or Lewy body disease, and unspecified MNCD.
Interventions	The exergame group (N = 7) trained with the VITAAL exergame prototype, specially designed for the rehabilitation of older adults. They performed both physical exercise such as strength, balance, and cognitive training. Some of the games were inspired by Tai Chi such as squats; some focused on balance training (falling books, music, labyrinth, and mommy chicken); others targeted cognitive training (healthy food and pizza) or task switching (shopping) or short‐term memory training (shopping list). The alternative treatment group (N = 11) performed a 15‐min walk and 15 min of standardised squatting and stepping exercises, similar to the exercises that were performed in the VITAAL minigames. Both groups trained for 3 individual sessions per week for 12 weeks, with 30 minutes per session.
Outcomes	Outcomes were recorded at baseline and end of treatment (12 weeks) and consisted of physical functioning measures (e.g. 1‐minute STS (Bohannon 1995)); cognitive functioning measures (e.g. MMSE (Folstein 1975)); quality of life in dementia (DQoL, Brod 1999); and functional status (SPPB, Guralnik 1994).
Notes	Feasibility study conducted in Belgium; also included a qualitative data analysis section. No follow‐up assessments

**Table:** Torpil 2020

***Study characteristics***	
Methods	RCT with 2 groups: exergaming and cognitive rehabilitation
Participants	64 people with a diagnosis of MCI based on Petersen criteria were randomised. 61 participants completed the assessments at the end of treatment.
Interventions	The exergame group (N = 30) trained using Microsoft Kinect for PC where participants played 4 different games that can be played with Kinect and purchased commercially in the online system: boxing trainer, jet run, superkick, air challenge. The alternative treatment group (N = 31) received cognitive training delivered using the LOTCA‐G programme (Itzkovich 1996). The first 8 weeks focused on training the following cognitive functions separately: attention/concentration, memory, orientation, visual perception, spatial perception, visuomotor organisation, praxis, and thinking operation. The final 4 weeks consisted of interventions that targeted all cognitive functions. Both groups performed 45‐minute sessions twice a week for 12 weeks.
Outcomes	Outcomes were recorded at baseline and end of treatment (12 weeks), and included global cognitive functioning measures (LOTCA‐G, Itzkovich 1996).
Notes	The study was conducted in Turkey; no follow‐up assessments.

CAMCI: Computer Assessment of Memory and Cognitive Impairment; DQoL: Dementia Quality of Life Questionnaire; DSM: Diagnostic and Statistical Manual of Mental Disorders; DS: Digit Span Forward; EFIP: Evaluative Frailty Index for Physical Activity; FICSIT: Frailty and Injuries Cooperative Studies of Interventions Technique Subtest 4; FRT: Functional Reach Test); HOTAP: HOTAP–Handlungsorganisation und Tagesplanung; HRV: heart rate variability; ICD: International Classification of Diseases; LLT: Location Learning Test; LOTCA‐G: Loewenstein Occupational Therapy Cognitive Assessment‐Geriatric; m: metres; min: minutes; MCI: mild cognitive impairment; MMSE: Mini Mental State Examination; MoCA: Montreal Cognitive Assessment; MNCD: major neurocognitive disorder; mNCD: mild neurocognitive disorder; MYHAT: Monongahela‐Youghiogheny Healthy Ageing Team; N: number of participants; NIA‐ AA: National Institute on Aging and Alzheimer's Association; QMCI: Quick Mild Cognitive Impairment Screen; QoL‐AD: Quality of Life in Alzheimer's disease; RAVLT: Rey Auditory Verbal Learning Test; R‐OCFT: Rey–Osterrieth Complex Figure Test; RSCT: Rule Shift Cards Test; SF‐36: Short‐Form Health Survey; S‐IADL: Seoul‐Instrumental Activities of Daily Living; SST: Sit to Stand Test; SPPBT: Short Physical Performance Battery Test; TIADL: Timed Instrumental Activities of Daily Living Tasks; TMT: Trail Making Test; TUG: Timed Up and Go Test; VFT: Verbal Fluency Test; VR: virtual reality; WAIS: Wechsler Adult Intelligence Scale; WMS: Wechsler Memory Scale.

### Characteristics of excluded studies [ordered by study ID]

**Table:**

Study	Reason for exclusion
Amjad 2019	Diagnosis was not based on established criteria, e.g. Petersen
Anderson‐Hanley 2012	Wrong population
Anderson‐Hanley 2017	Wrong population
Anderson‐Hanley 2018a	Wrong intervention
Anderson‐Hanley 2018b	Diagnosis was not based on established criteria, e.g. Petersen
Bamidis 2015	Wrong intervention
Choi 2012	Wrong intervention
Choi 2019	Wrong intervention
Choi 2019b	Wrong intervention
Del Din 2020	Diagnosis was not based on established criteria, e.g. Petersen
Delbroek 2017	Diagnosis was not based on established criteria, e.g. Petersen
Embon‐Magal 2022	Diagnosis was not based on established criteria, e.g. Petersen
Jhaveri 2023	Diagnosis was not based on established criteria, e.g. Petersen
Karssemeijer 2017	Wrong study design
Kwan 2021	Diagnosis was not based on established criteria, e.g. Petersen
Liao 2019	Wrong population
Liao 2019b	Wrong intervention
Maidan 2017	Wrong population
Mirelman 2016	Diagnosis was not based on established criteria, e.g. Petersen
Mrakic‐Sposta 2018	Wrong intervention
NCT01732653	Diagnosis not based on established criteria, e.g. Petersen
NCT03069391	Diagnosis was not based on established criteria, e.g. Petersen
Padala 2012	Diagnosis was not based on established criteria, e.g. Petersen
Padala 2017a	Additional data sought from authors but not provided
Padala 2017b	Additional data sought from authors but not provided
Park 2018b	Wrong intervention
Uğur 2020	Diagnosis was not based on established criteria, e.g. Petersen
Van Santen 2020	Diagnosis was not based on established criteria, e.g. Petersen
Van Santen 2021	Wrong outcomes
Vermeylen 2016	Diagnosis was not based on established criteria, e.g. Petersen
Wiloth 2018	Wrong intervention
Wu 2023	Diagnosis did not meet our inclusion criteria
Zhao 2022	Diagnosis was not based on established criteria, e.g. Petersen
Zheng 2022	Diagnosis did not meet our inclusion criteria

### Characteristics of studies awaiting classification [ordered by study ID]

**Table:** Ho‐yin Lai 2020

Methods	RCT comparing the effects of VR‐based training vs home hazard identification training
Participants	100 community‐dwelling people with moderate dementia (CDR > 2)
Interventions	The exergame group VR‐based home safety alertness for near miss for fall prevention. The control group received reviews of home safety and fall prevention. Both groups reveived an initial 1‐month psychoeducation programme on home safety and fall prevention in their own home. After this they were randomised to one of the groups and underwent treatment for the next 11 months.
Outcomes	Falls
Notes	No mention of diagnostic criteria, e.g. ICD or DSM. No clear mention about other outcomes such as caregiver outcomes. No thorough description of interventions Conference abstract. Categorised as awaiting classification because we were unable to obtain (via thorough web search and after contacting the corresponding author) a published full‐text version of this study. It may be the case that it is ongoing work.

**Table:** NCT01000038

Methods	RCT comparing the effects of Wii Fit vs walking
Participants	22 participants with Alzheimer's dementia, mild (MMSE ≥ 18)
Interventions	The exergame group had Wii Fit excercises for 30 minutes daily and the control group had walking excercises. Participants in each group received 30‐min long daily sessions for 5 days a week for 8 weeks.
Outcomes	Outcomes were recorded at 8 weeks and included physical domains (e.g. gait, balance), cognitive domains (e.g. global cognition, executive functioning), and activties of daily living
Notes	Clinical trial registry. Categorised as awaiting classification because we were unable to obtain at this time (via thorough web search and after contacting the corresponding author) a published full‐text version of this study. No mention of diagnostic criteria, e.g. ICD or DSM

**Table:** NCT01002586

Methods	RCT comparing the effects of Wii Fit vs walking
Participants	22 participants with Alzheimer's dementia, mild (MMSE ≥ 18)
Interventions	The exergame group had Wii Fit excercises for 30 minutes daily and The control group had walking excercises. Participants in each group received 30‐min long daily sessions for 5 days a week for 8 weeks.
Outcomes	Outcomes recorded at 8 and 16 weels. Included physical domains (e.g. gait, balance), cognitive domains (e.g. global cognition, executive functioning), and activties of daily living
Notes	No mention about diagnostic criteria, e.g. ICD or DSM Clinical trial registry. Categorised as awaiting classification because we were unable to obtain at this time (via thorough web search and after contacting the corresponding author) a published full‐text version of this study.

**Table:** NCT01393353

Methods	Cognitive training in Parkinson's disease vs Nintendo Wii
Participants	Parkinson's disease and mild cognitive impairment (MCI) or mild dementia
Interventions	CogniPlus vs Nintendo Wii
Outcomes	Cognitive function measured with tests of episodic memory, working memory, executive functions, visio‐construction
Notes	No mention of diagnostic criteria, e.g. ICD or DSM. No thorough description of interventions Clinical trial registry. Categorised as awaiting classification because we were unable to obtain at this time (via thorough web search and after contacting the corresponding author) a published full‐text version of this study.

**Table:** NCT02214342

Methods	RCT comparing virtual reality based balance training vs no intervention
Participants	20 people with MCI
Interventions	The exergame group received balance training via VR and performed tasks such as "ankle reaching" and "obstacle crossing" using a virtual obstacle shown on a computer screen. VR balance training was administered individually 2 times per week for 4 weeks, and each session lasted for 30 to 45 minutes. The control group kept their normal activity without receiving any intervention.
Outcomes	Physical function (e.g. gait), cognitive function, and user experience were measured at 4 weeks.
Notes	No mention of diagnostic criteria, e.g. Petersen or DSM Clinical trial registry. Categorised as awaiting classification because we were unable to obtain at this time (via thorough web search and after contacting the corresponding author) a published full‐text version of this study. It may be the case that it is ongoing work.

**Table:** NCT02237560

Methods	RCT to compare the effects of cybercycling (virtual reality enhanced stationary cycling) with physical and mental exercise alone
Participants	111 persons at risk for and with MCI
Interventions	The first exergame group performed a combined aerobic and cognitive exercise called Cybercycle‐Game where participants exercised on a virtual‐reality enhanced stationary recumbent bicycle while playing interactive 3D video‐game for 6 months, 3 to 5 times a week. The second exergame group underwent aerobic excercises on a virtual‐reality enhanced stationary recumbent bicycle and pedal through interactive 3D scenic bike tours for 6 months, 3 to 5 times a week. The control group consisted of cognitive exercises performed while seated on a stationary recumbent seat play interactive 3D video‐game for 6 months, 3 to 5 times a week (no pedalling).
Outcomes	Primary outcome was executive functioning (e.g. Digit Span Backwards, Stroop, and Color Trails) and was measured at 6 months. Secondary outcomes were executive function at 3 months (e.g. Digit Span Backwards, Stroop, and Color Trails) and at 12‐month follow‐up. Other outcomes such as cognitive functioning measured with MoCA, ADAS‐Cog word recall, complex figures, and controlled oral word association test at 6 months.
Notes	No mention of diagnostic criteria e.g. Petersen or DSM Clinical trial registry. Categorised as awaiting classification because we were unable to obtain at this time (via thorough web search and after contacting the corresponding author) a published full‐text version of this study. It may be the case that it is ongoing work.

**Table:** NCT03383549

Methods	RCT to compare combined cognitive and physical excercises tele‐rehabilitation vs control
Participants	60 participants with cognitive dysfunction, mild cognitive impairment vascular cognitive impairment
Interventions	Tele‐rehabilitation group received home‐based rehabilitation made up of cognitive and physical exercises. Intervention duration consists of daily session between 30 to 45 minutes for 8 weeks. The control group received verbal instructions to train cognitive and physical conditions.
Outcomes	Primary outcome was general cognition (e.g. MoCA) measured at 8 weeks. Secondary outcomes were cognitive domains measures (e.g. Stroop, Verbal Fluency Task) and were measured at 8 weeks.
Notes	Clinical trial registry. Categorised as awaiting classification because we were unable to obtain at this time (via thorough web search and after contacting the corresponding author) a published full‐text version of this study. It may be the case that it is ongoing work.

**Table:** NCT03774225

Methods	Single‐blind RCT to compare balance exergame training vs conventional training
Participants	49 participants with Parkinson's disease
Interventions	The first exergame group performed balance rehabilitation with manual and verbal guidance of a physiotherapist using 4 games of X‐Box Kinect system. The second exergame group performed balance rehabilitation using X‐Box Kinect system alone. The control group was trained with conventional balance/physical therapy exercises.
Outcomes	Physical function (e.g. balance and gait) at 5 and 8 weeks
Notes	No mention of any cognitive impairment Clinical trial registry. Categorised as awaiting classification because we were unable to obtain at this time (via thorough web search and after contacting the corresponding author) a published full‐text version of this study. It may be the case that it is ongoing work.

**Table:** NCT03928405

Methods	RCT to compare the effects of balance exercises with Nintendo Wii vs control
Participants	32 participants with mild or moderate AD
Interventions	The exergame group performed exercises with Nintendo Wii. The games were selected from different categories such as balance and aerobic exercises. Intervention lasted for 6 weeks, one session of 30 min, 2 times a week. In the control group, no treatment was administered and routine medical treatment was continued
Outcomes	Primary outcome measures were measures of gait and balance and global cognition
Notes	Diagnosis made by a neurologist Clinical trial registry. Categorised as awaiting classification because we were unable to obtain at this time (via thorough web search and after contacting the corresponding author) a published full‐text version of this study. It may be the case that it is ongoing work.

**Table:** NCT04377191

Methods	Non‐RCT comparing exergaming with standard excercise
Participants	30 participants diagnosed with dementia
Interventions	The first exergame group perfromed standard exercises and exergame training using Microsoft Xbox 360 Kinect. The second exergame group received standard exercise and exergame training with ALDA balance gear. In the control group participants received standard exercise. Groups received training for 2 days per week for 6 weeks.
Outcomes	Balance and gait were measured at 6 weeks.
Notes	No mention about diagnostic criteria e.g. ICD or DSM Labelled as non‐RCT in the protocol registration, awaiting additional information to judge if it followed an RCT design or not Clinical trial registry. Categorised as awaiting classification because we were unable to obtain at this time (via thorough web search and after contacting the corresponding author) a published full‐text version of this study. It may be the case that it is ongoing work.

**Table:** NCT04819126

Methods	RCT to examine the effect of Nintendo Wii virtual reality application vs control
Participants	People with Alzheimer's dementia
Interventions	The exergame group received balance and aerobic exercises with Nintendo Wii 2 times a week for a period of 6 weeks, with 1 session lasting for 30 minutes. The control group received routine medical treatment.
Outcomes	Global cognition measured with the Mini‐Mental State Test at 6 weeks
Notes	Clinical trial registry. Categorised as awaiting classification because we were unable to obtain at this time (via thorough web search and after contacting the corresponding author) a published full‐text version of this study. It may be the case that it is ongoing work.

**Table:** Padala 2019

Methods	RCT to assess the effects of exergame vs waliking
Participants	22 people with mild Alzheimer's disease
Interventions	The exergame group performed exercise using Nintendo Wii‐Fit. The control group were in a walking programme. Both groups performed exercises for 30 minutes, 5 days per week for 8 weeks in the presence of research assistant.
Outcomes	Executive function was measured with Trails Making Test A & B and EXIT‐25 at eight weeks.
Notes	No mention about diagnostic criteria, e.g. ICD or DSM Conference abstract. Categorised as awaiting classification because we were unable to obtain at this time (via thorough web search and after contacting the corresponding author) a published full‐text version of this study. It may be the case that it is ongoing work.

**Table:** RBR‐3bq4rg

Methods	RCT to compare the effects of physical exercise with video game vs without video game
Participants	50 people with diagnoses of Unspecified Dementia, Mental Disorders, or Neurocognitive Disorders
Interventions	The exergame group perform exercises via the Nintendo Wii video game System, using Balance Board devices (force platform that captures pressure centre oscillations), and Wii Motion and Nunchuck (hand controls that via bluetooth capture the movements that the participant makes). The control group perform the same type of exercises as the exergame group without the exergame platform. The physical exercises will be performed twice a week, totaling 16 sessions. Each session will take around 30 to 45 minutes in total.
Outcomes	Physical (e.g. Berg Balance Scale) and cognitive (e.g. MMSE, digit span, Floor maze test, Stroop test, Clock test, Verbal fluency test, and MoCA)
Notes	Inclusion criteria: "having Clinica [sic] Dementia Rating 0.5; [sic] 1 and 2, with a minimum of three years of formal study" Clinical trial registry. Categorised as awaiting classification because we were unable to obtain at this time (via thorough web search and after contacting the corresponding author) a published full‐text version of this study. It may be the case that it is ongoing work.

**Table:** TCTR20181001001

Methods	Single‐blind RCT
Participants	People with MCI. MMSE score more than 24, MoCA score less than 26
Interventions	The exergame group used virtual reality for exercising. The control group performed exercises as strengthening exercise, aerobic exercise, and balance exercise without VR.
Outcomes	Cognitive functioning
Notes	No mention of diagnostic criteria, e.g. DSM or ICD, but mention of cut‐off scores: MMSE score more and 24, MoCA score less than 26 Clinical trial registry states study was completed in 2018. Categorised as awaiting classification because we were unable to obtain at this time (via thorough web search and after contacting the corresponding author) a published full‐text version of this study.

AD: Alzheimer's Disease; ADAS‐Cog: Alzheimer Disease Assessment Scale‐cognitive section; CDR: Clinical Dementia Rating; DSM: Diagnostic and Statistical Manual of Mental Disorders; ICD: International Classification of Diseases; MCI: mild cognitive impairement; MMSE: Mini Mental State Examination; MoCA: Montreal Cognitive Assessment; RCT: randomised controlled trial; VR: virtual reality; vs: versus

### Characteristics of ongoing studies [ordered by study ID]

**Table:** ACTRN12617000364370

Study name	The effect of cognitive training on cognitive function in older people with mild to moderate dementia: a single‐blind randomised control trial
Methods	Single‐blind RCT
Participants	Community‐dwelling participants with mild to moderate dementia
Interventions	The intervention group performed games based on popular arcade games such as: Dance Dance Revolution, Tetris, Snake, and Space Invaders. Participants were encouraged to train at least 3 times per week for 10 to 30 min a session (total recommended dose 90 min).
Outcomes	Cognitive functioning (e.g. executive functioning, global cognition measured with Digit Span, TMT A & B, Addenbrooke's Cognitive Examination III), physical functioning (e.g. gait, balance), quality of life, physical activity, measured at 6 and 12 months
Starting date	10 March 2017
Contact information	Dr Morag Taylor, m.taylor@neura.edu.au
Notes	Diagnosis made by specialist diagnosis or MMSE < 24 or Addenbrooke’s Cognitive Examination III score of < 83; no mention of control group

**Table:** ChiCTR2100043753

Study name	Construction and empirical study of community intervention program for patients with mild cognitive impairment based on immersive virtual reality cognitive training system
Methods	RCT to evaluate the intervention effect of VR cognitive intervention vs control
Participants	70 people with MCI
Interventions	The exergame group underwent immersive virtual reality cognitive training. The control group performed traditional pen and paper cognitive training.
Outcomes	Cognitive functioning (e.g. global cognition measured with MoCA, MMSE), ADL
Starting date	25 February 2021
Contact information	Li Hong, leehong99@126.com
Notes	Diagnostic based on established criteria; no detailed description of interventions

**Table:** ChiCTR2100048696

Study name	Construction and evaluation of executive function intervention program for patients with vascular mild cognitive impairment by immersive exercise and cognitive training
Methods	Interventional study with 2 groups
Participants	60 people with vascular mild cognitive impairment
Interventions	Experimental group will perform immersive exercise and cognitive training in hospital. Control group will perform traditional sports and cognitive training at home.
Outcomes	Cognitive functioning (e.g. executive functioning), physical functioning (e.g. balance)
Starting date	1 May 2021
Contact information	Tian Siying, School of Nursing, Capital Medical University, tiansiying1510918@163.com
Notes

**Table:** IRCT20211110053030N2

Study name	The effect of video games on cognitive performance and problem solving ability in the elderly
Methods	RCT
Participants	Older adults with MCI
Interventions	Intervention group will play three selected games for 12 weeks. Control group will not receive any educational intervention.
Outcomes	Cognitive functioning
Starting date	16 July 2023
Contact information	Alireza Vasiee, rezawest10@gmail.com
Notes

**Table:** IRCT20220120053773N1

Study name	Effect of virtual reality intervention on mild cognitive impairment
Methods	Double‐blind RCT
Participants	People with MCI
Interventions	Intervention group received VR basedrehabilitation including two games, "targeting" and "maze"
Outcomes	Cognitive and physical functioning
Starting date	22 May 2022
Contact information	Kimia Darmiani, kimia.darmiani80@gmail.com
Notes	Type of control group not specified

**Table:** ISRCTN85177142

Study name	Virtual reality‐based exercise in elderly people, people with Alzheimer's disease and people with schizophrenia
Methods	RCT
Participants	People with I or II stage Alzheimer's disease
Interventions	Participants in the experimental group will receive VR Kinect exercise.
Outcomes	Cognitive and physical functioning, ADL, quality of life
Starting date	27 March 2023
Contact information	Laura Piejko, l.piejko@awf.katowice.pl
Notes	Type of control group not specified

**Table:** KCT0008238

Study name	Effects of exercise games on improving cognitive function and health promotion in the elderly with dementia
Methods	Interventional study
Participants	52 individuals with dementia aged 60 or older
Interventions	Exergame group or stationary bike group
Outcomes	Physical and cognitive functioning
Starting date	4 January 2019
Contact information	Jung‐Jun Park, jjparkpnu@pusan.ac.kr
Notes	Retrospective registration

**Table:** NCT03911765

Study name	Effect of digital cognitive training on the functionality of older adults with mild cognitive impairment (MCI)
Methods	Cross‐over RCT
Participants	40 people with MCI
Interventions	Intervention group underwent executive function cognitive training for 20 hours. The control group played computer games available online, e.g. fishing game, pinball game, tetris, etc, followed by 10 hours of of digital cognitive training targeting executive function.
Outcomes	Cognitive functioning (e.g. memory, attention, global cognition measured with MoCA), ADL
Starting date	11 April 2019
Contact information	Dr Rogério Panizzutti, rogerio.panizzutti@ipub.ufrj.br
Notes	No details concerning diagnostic criteria, e.g. Petersen or DSM; not enough detail to judge if intervention fits our criteria

**Table:** NCT04271943

Study name	The effect of aerobic and game based exercises on cognitive functions in dementia
Methods	Single‐blind RCT
Participants	Older adults with dementia
Interventions	The intervention group will perform computer‐based exercises using the See‐ME Virtual Rehabilitation system. The control group will perform aerobic exercises.
Outcomes	Cognitive functioning
Starting date	20 April 2019
Contact information	Istanbul Medipol University Hospital
Notes	Not enough information to assess if the intervention fits our eligibility criteria

**Table:** NCT04415775

Study name	Changes in metabolic activity, and gait function by dual‐task cognitive game‐based treadmill intervention in Parkinson's disease (CMAGDT)
Methods	RCT
Participants	67 people with Parkinson's disease
Interventions	The exergame group consisted of game‐based treadmill programme where participants played several cognitive computer games while standing on a sponge pad. The control group underwent conventional gait rehabilitation via treadmill walking.
Outcomes	Gait
Starting date	4 June 2020
Contact information	Dr. Tony Szturm, tony.szturm@umanitoba.ca
Notes	No mention of cognitive impairment

**Table:** NCT04730817

Study name	VR motor‐cognitive training for older people with cognitive frailty
Methods	Single‐blind RCT
Participants	400 people with cognitive frailty
Interventions	The exergame group performed VR simultaneous motor‐cognitive training in 30 minutes session, 2 times a week for 8 weeks. The control group did not receive any treatment.
Outcomes	Cognitive functioning such as global cognitive function (e.g. MoCA), attention and executive functioning (e.g. Stroop, TMT A & B), short‐term memory (e.g. Digit Span), lower limb function (e.g. TUG) and upper limb function (e.g. hand grip strength by dynamometer), frailty (e.g. FFP) after 8 weeks
Starting date	1 March 2021
Contact information	Dr Rick Kwan, rickkwan@twc.edu.hk
Notes

**Table:** NCT04732182

Study name	Telerehabilitation Alzheimer's Disease Feasibility (TADF)
Methods	Single‐blind RCT
Participants	14 participants with a diagnosis of early Alzheimer's (MoCA score of 19 to 25)
Interventions	The exergame group received standard of care and 8 weeks of experimental computer‐based therapy on the device at home (BrightGo). The control is waitlist group with standard of care.
Outcomes	Cognitive functioning (e.g. attention, executive functioning), quality of life, physical functioning (upper limb)
Starting date	16 February 2022
Contact information	Dr Grigore C Burdea, diplomatru@yahoo.com
Notes	No mention od the criteria for dementia diagnosis, e.g. DSM or ICD

**Table:** NCT04959383

Study name	Exergame balance training for patients with mild cognitive impairment
Methods	Double‐blind RCT
Participants	90 participants with MCI
Interventions	Three exergame groups (low, moderate, high) underwent exergame balance training. Control group underwent Wii‐Fit‐based exergame training on a stable surface.
Outcomes	Cognitive functioning (e.g. attention and executive functioning, global cognition measured with MoCA), physical functioning (e.g. gait and balance)
Starting date	23 September 2021
Contact information	Dr Anjad, imran.amjad@riphah.edu.pk
Notes	No mention about the criteria for dementia diagnosis, e.g. DSM or Petersen

**Table:** NCT05009524

Study name	The Interactive Physical and Cognitive Exercise System (v3) for mild cognitive impairment (MCI) (iPACES)
Methods	RCT
Participants	120 participants with MCI, Alzheimer's disease, dementia
Interventions	The exergame group was trained with the iPACES (interactive Physical and Cognitive Exercise System) ‐ a "pedal‐to‐play" neuro‐exergame in which physical and mental exercise are combined in an interactive way. Participants will pedal to control forward motion in a tablet‐based game, such as when pedalling along a virtual path and steering to different assigned errand locations. The control group will undergo the non‐interactive iPACES.
Outcomes	Executive functioning measured at 6 months
Starting date	23 August 2021
Contact information	andersoc@union.edu
Notes	Not enough information concerning the intervention and control to assess eligibility

**Table:** NCT05158595

Study name	Exer‐game balance training on dementia
Methods	Single‐blind RCT with 4 arms
Participants	People with MCI
Interventions	Experimental group 1: low‐intensity group trained using wobble‐board‐based exergame balance training, low game intensity Experimental group 2: moderate‐intensity group received wobble‐board‐based exergame balance training Experimental group 3: high‐intensity group received wobble‐board‐based exergame balance training, with high game intensity Control group 1: exergame balance training with Wii Fit balance games
Outcomes	Lower limb functioning (e.g. balance)
Starting date	15 August 2021
Contact information	Dr. Arshad Nawaz Malik, arshad.nawaz@riphah.edu
Notes

**Table:** NCT05227495

Study name	Effects of the fully immersive leisure‐based virtual reality cognitive training for community‐dwelling elderly
Methods	Double‐blind RCT
Participants	60 community‐dwelling elderly people
Interventions	Intervention group will receive an immersive leisure‐based VR cognitive training for 40 min per day, 2 days per week for 16 sessions. The control group will receive a traditional cognitive training programme for 60 min per day, 2 days per week for 16 sessions.
Outcomes	Cognitive functioning (e.g. memory, attention, executive functioning, global cognition measured with MoCA), balance lower and upper limb (Grip strength and Timed up and go), ADL
Starting date	11 April 2021
Contact information	Chang Gung Memorial Hospital
Notes	No details concerning diagnostic criteria

**Table:** NCT05235113

Study name	Remote game‐based exercise program for cognitive and motor function Improvement
Methods	Double‐blind RCT
Participants	100 participants with MCI and dementia
Interventions	The intervention group performed excercises via a tele‐exergaming platform. The control group performed excercises without the tele‐exergaming platform. Both groups performed exercises twice a week.
Outcomes	Physical functioning (e.g. balance and gait), cognitive functioning, quality of life
Starting date	22 December 2021
Contact information	Dr Bijan Najaf, bijan.najafi@bcm.edu
Notes	No mention about the criteria for dementia diagnosis, e.g. DSM or Petersen

**Table:** NCT05387057

Study name	Effectiveness of a novel exergame‐based training concept for older adults with mild neurocognitive disorder
Methods	Single‐blind RCT
Participants	41 older adults with mild neurocognitive disorder (mNCD)
Interventions	Intervention group trained using an exergame‐based motor‐cognitive training concept (called 'Brain‐IT'). Control group continued with their usual care
Outcomes	Cognitive functioning (e.g. memory, attention, global cognition measured with Quick Mild Cognitive Impairment Screen), gait, ADL
Starting date	22 June 2022
Contact information	Eling D de Bruin, PhD, ETH Zurich, eling.debruin@hest.ethz.ch
Notes

**Table:** NCT05402423

Study name	An integrated intervention of computerized cognitive training and physical exercise in virtual reality for people with Alzheimer's disease (JDome)
Methods	RCT
Participants	Older adults with a pre‐existing diagnosis of Alzheimer's, in mild phase, according to the 2011 criteria of the National Institute on Aging‐Alzheimer's Association (NIA‐AA)
Interventions	The intervention group will take part in computerised cognitive training with the Brainer web platform and aerobic training with the JDOME system (combination of an exercise bike, a dome‐shaped projector, and Google Street View technology). The control group will receive computerised cognitive training with the Brainer web platform and aerobic training with a standard exercise bike.
Outcomes	Cognitive functioning (e.g. global cognition), physical functioning, quality of life.
Starting date	27 June 2022
Contact information	Giuseppe Pelliccioni, MD,IRCCS INRCA, Ancona, Italy, g.pelliccioni@inrca.it
Notes

**Table:** NCT05603533

Study name	Adherence by Music to Exercise in Dementia: group therapy (AMUSED)
Methods	Single‐blind RCT
Participants	Older adults with moderate dementia
Interventions	Intervention group perform regular physical therapy; participants in the intervention group receive a music and video‐based group exercise therapy in small groups of 5. Control group receive no additional therapy.
Outcomes	Physical functioning (upper and lower limb), cognitive functioning (e.g. general cognition), quality of life
Starting date	9 January 2023
Contact information	Hasselt University
Notes

**Table:** NCT05730790

Study name	Immersive Virtual Reality for dual‐task training in older adults with mild cognitive impairment: a randomized controlled trial
Methods	RCT
Participants	Older adults with MCI
Interventions	Intervention group 1 receives cognitive‐motor dual task training (DTT) using a VR system while walking on a treadmill Control group 2 receives cognitive single task training (CSTT) using a VR system Control group 1 receives motor single task training (MSTT) walking on a treadmill
Outcomes	Physical functioning (e.g. gait and balance), cognitive functioning (e.g. executive functioning)
Starting date	31 March 2021
Contact information	Dr. Kwee Yong Joyce Yap, Tan Tock Seng Hospital
Notes

**Table:** NCT05810454

Study name	iPACES v3 MCI NIA protocol copied for iPACES v4 PD NINDS (iPACES‐PD)
Methods	RCT
Participants	Peoples aged 50 years or older with MCI
Interventions	Intervention group will train with iPACES (interactive Physical and Cognitive Exercise System) involving a "pedal‐to‐play" neuro‐exergame in which physical and mental exercise are combined in an interactive way. Control group will train using the PACE (Physical and Cognitive Exercise) involving a "pedal‐while‐play" experience in which physical and mental exercise are combined in a simultaneous, but not fully interactive way.
Outcomes	Cognitive functioning, ADL
Starting date	17 October 2023
Contact information	Cay Anderson‐Hanley, PhD, iPACES LLC, andersoc@union.edu
Notes	Registration entry does not state what the acronyms NIA and NINDS stand for.

**Table:** NCT06079411

Study name	Virtual therapeutics for MCI (VR‐MCI)
Methods	RCT
Participants	Older adults with MCI
Interventions	Experimental group 1 will perform VR embodied spatial training. Experiemental group 2 will perform non‐embodied spatial training. The control group will receive treatment as usual.
Outcomes	Primary outcomes are the long‐term visuospatial memory scores from Corsi supra span and the spatial rotation score from the Manikin Test.
Starting date	October 2023
Contact information	Cosimo Tuena, c.tuena@auxologico.it
Notes

**Table:** NCT06129630

Study name	Effects of Nintendo Switch on rehabilitation programs for elderly people with cognitive impairment
Methods	Single‐blind RCT
Participants	Older adults aged 65 years or older living in a nursing home
Interventions	Experimental group received therapy with Nintendo Switch, plus their conventional occupational therapy sessions Control group received their usual sessions of conventional occupational therapy
Outcomes	Physical functioning
Starting date	1 March 2023
Contact information	Cristina García‐Bravo, Universidad Rey Juan Carlos
Notes

**Table:** RBR‐4kdqcbt 2022

Study name	Effect of a two‐task exercise in Virtual Reality on the physical and mental point of view in the elderly: a randomized clinical trial
Methods	RCT
Participants	30 older adults with cognitive modifications on the MMSE
Interventions	Experimental group will participate in a dual‐task training programme using Immersive Virtual Reality; examples of games are: ball game for mobility and reasoning, gait training with obstacle avoidance. Control group will participate in a conventional dual‐task training programme, carried out through games and activities for cognitive‐motor stimulation.
Outcomes	Cognitive functioning, physical functioning, ADL
Starting date	3 August 2022
Contact information	Felipe Lima Rebêlo, feliperebelo_fisio@yahoo.com.br
Notes	Not enough information to judge if the diagnosis of MCI follows established criteria, e.g. Petersen criteria

**Table:** TCTR20210530003

Study name	Effects of exergaming based Tai Chi on cognition and dual task gait in older adults with mild cognitive Impairment: a randomized control trial
Methods	RCT
Participants	50 people with a diagnosis of MCI according to Petersen criteria
Interventions	The intervention group performed exergaming‐based Tai Chi using Kinect.
Outcomes	Physical (e.g. gait) and cognitive performance
Starting date	30 May 2021
Contact information	Yingyi Liao, ianliao1209@gmail.com
Notes	No details about the control group

**Table:** University of Missouri‐Columbia

Study name	Cognitive Training in Mild Cognitive Impairment (TRAIN‐MCI)
Methods	Single‐blind RCT
Participants	50 participants with insomnia and MCI
Interventions	The intervention focused on a video game training. The computerised training exercises and brain games help stimulate cognitive functions while providing real‐time monitoring on the evolution of participants' skills. The control group underwent a computerised trivia training where participants answer general questions related to pop culture, science, geography, etc. Both groups played the games for 45 minutes 3 times per week.
Outcomes	Cognitive functioning measured at 10 weeks
Starting date	28 July 2021
Contact information	Ashley Curtis, curtisas@health.missouri.edu
Notes	No details concerning MCI criteria

ADL: activities of daily living; CDR: Clinical Dementia Rating; FFP: Fried Frailty Phenotype; iPACES: interactive Physical and Cognitive Exercise System; MCI: mild cognitive impairment; min: minutes; MMSE: Mini‐Mental State Examination; MoCA: Montreal Cognitive Assessment; PD: Parkinson's Disease; RCT: randomised controlled trial; TMT A & B: Trail Making Test part A & B; TUG: Timed Up and Go Test; VR: virtual reality; vs: versus

## Differences between protocol and review

In our protocol, we had not said we would include cluster‐RCTs, but we included two in the review. For these, we used the appropriate risk of bias tool (RoB2) for cluster‐RCTs.

For all comparisons at follow‐up time points, we had planned to assess RoB and perform GRADE assessment, and to present the results in summary of findings tables. Due to the small number of studies available for inclusion and low power, we decided against this. We prioritised the comparisons, and we produced summary of findings tables for primary outcomes at the end of treatment only, to ensure that the most important information was highlighted in the summary sections of the review.

In our protocol, we had planned to conduct the following subgroup analyses to investigate potential sources of heterogeneity: severity of dementia, intervention characteristics, type of control intervention, type of exergame platform, type of technology, length of intervention, and length of follow‐up. We were unable to test any of these moderators as we did not have enough studies to perform subgroup analyses.

We had planned some sensitivity analyses to test the robustness of our results. For example, we had planned to compare our results with those we would have found if we had not included studies at higher risk of bias. However, because none of the studies were at low risk of bias overall, we did not conduct these sensitivity analyses. Due to limited data, we decided not to run planned sensitivity analyses excluding studies that were non‐dose‐matched or studies with low statistical power. In our protocol, for people with MCI, we had planned to record the definitions used by study authors (e.g. National Institute on Aging‐Alzheimer’s Association, International Working Group on Mild Cognitive Impairment or MCI Working Group of the European Consortium on Alzheimer’s Disease), and consider these as a possible source of heterogeneity, performing sensitivity analysis, if necessary. This was not possible as studies used Petersen 1999 or an adapted version of Petersen criteria, thus eliminating heterogeneity amongst the studies. We had initially planned to conduct a sensitivity analysis to compare the robustness of our analysis using change scores versus post‐intervention scores only. However, in light of recent Cochrane reviews (e.g. Kudlicka 2023), which used a similar methodology by computing change‐from‐baseline scores and including them in their analyses without performing any sensitivity analysis, we decided not to proceed with this sensitivity analysis.

We aimed to investigate reporting bias using funnel plots and Duval 2000’s trim‐and‐fill procedure. However, we only included a maximum of four studies per comparison, which did not reach the recommended threshold of 10 studies to carry out such a procedure.
